# Aromatic thiol-mediated cleavage of N–O bonds enables chemical ubiquitylation of folded proteins

**DOI:** 10.1038/ncomms12979

**Published:** 2016-09-29

**Authors:** Caroline E. Weller, Abhinav Dhall, Feizhi Ding, Edlaine Linares, Samuel D. Whedon, Nicholas A. Senger, Elizabeth L. Tyson, John D. Bagert, Xiaosong Li, Ohara Augusto, Champak Chatterjee

**Affiliations:** 1 Department of Chemistry, University of Washington, Seattle, Washington 98195, USA; 2 Departamento de Bioquímica, Instituto de Química-Universidade de São Paulo, São Paulo 05513-970, Brazil; 3 Department of Chemistry, Princeton University, Princeton, New Jersey 08544, USA

## Abstract

Access to protein substrates homogenously modified by ubiquitin (Ub) is critical for biophysical and biochemical investigations aimed at deconvoluting the myriad biological roles for Ub. Current chemical strategies for protein ubiquitylation, however, employ temporary ligation auxiliaries that are removed under harsh denaturing conditions and have limited applicability. We report an unprecedented aromatic thiol-mediated N–O bond cleavage and its application towards native chemical ubiquitylation with the ligation auxiliary 2-aminooxyethanethiol. Our interrogation of the reaction mechanism suggests a disulfide radical anion as the active species capable of cleaving the N–O bond. The successful semisynthesis of full-length histone H2B modified by the small ubiquitin-like modifier-3 (SUMO-3) protein further demonstrates the generalizability and compatibility of our strategy with folded proteins.

## Abstract (web summary)

Chemical approaches to site-specifically ubiquitylate a target protein allow investigation of the biochemical effects of this modification, but they often destabilize the protein. Here, the authors report on a synthetic conjugation strategy that leads to protein ubiquitylation in non-denaturing conditions.

## Graphical abstract

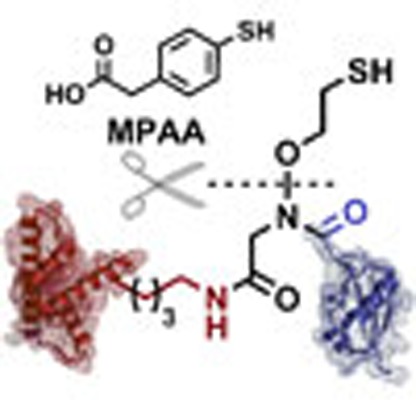

The reversible conjugation of proteins with ubiquitin and ubiquitin-like proteins is a post-translational modification conserved in all eukaryotic organisms^1^. The ubiquitin family consists of about 25 proteins, a majority of which can be conjugated with protein targets, either at specific Lys side-chain amines or at the protein N terminus^2^. For ubiquitin alone, there exist over 600 ligases that attach it to protein substrates in humans^3^. The many roles for protein modification by ubiquitin, termed ubiquitylation, include protein degradation, organelle-specific localization and the regulation of protein function^4^. Ubiquitylation is a dynamic modification and only a small fraction of proteins are ubiquitylated at any given time^5^, which complicates their isolation and hinders subsequent biochemical or biophysical studies aimed at unravelling the specific roles for ubiquitin.

In the last decade, a handful of chemical strategies have emerged that enable access to site-specifically ubiquitylated proteins or close analogs thereof^6^,^7^. These approaches, however, rely on challenging multi-step synthetic strategies, harsh denaturing conditions and/or a desulfurization of the final ubiquitylated product that is incompatible with cysteine residues^8^,^9^,^10^,^11^. In an attempt to overcome some of these limitations, we recently reported peptide ubiquitylation with the temporary ligation auxiliary, 2-aminooxyethanethiol, which employed reduction with metallic Zn in the terminal step^12^,^13^. Unfortunately, we found that efficient N–O bond reduction required both harsh denaturants and strongly acidic conditions. Therefore, our approach was ultimately limited to peptides or proteins amenable to refolding from the denatured state.

Herein we report the discovery of an unprecedented aromatic thiol-mediated N–O bond cleavage reaction that is compatible with folded proteins at physiological pH and that overcomes limitations of current strategies for chemical ubiquitylation. Mechanistic investigation of this new reaction implicates a disulfide radical anion as the reductive species that cleaves N–O bonds. The semisynthesis of full-length human histone H2B modified by the small ubiquitin-like modifier protein, SUMO-3, demonstrates the complete compatibility of this reaction with thiol side-chains in folded proteins and significantly expands the practical scope of chemical ubiquitylation.

## Results

### 4-Mercaptophenylacetic acid-mediated N–O bond cleavage

**Figure 1:**
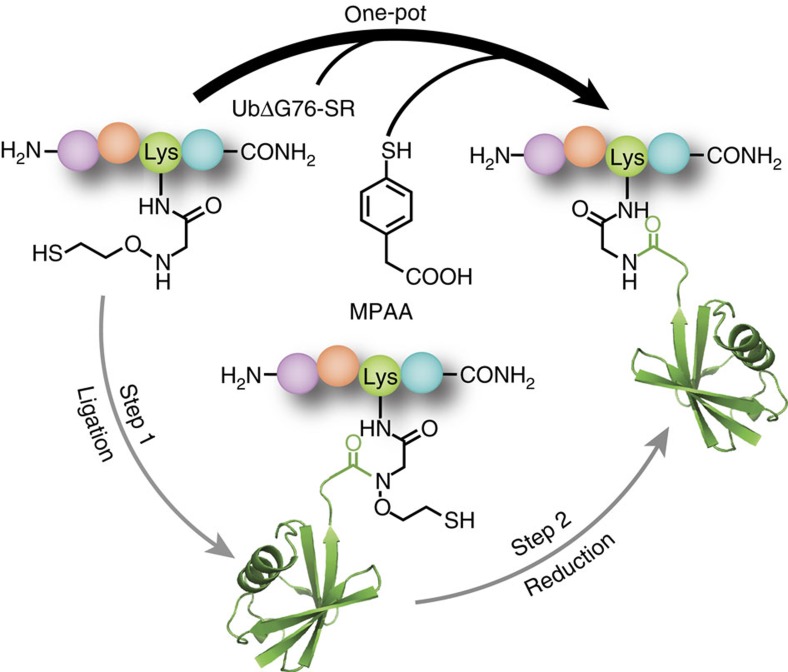
Aromatic thiol-mediated one-pot traceless native chemical ubiquitylation. MPAA, 4-mercaptophenylacetic acid; UbΔG76-SR, ubiquitin(1–75)-α-thioester with 2-mercaptoethanesulfonic acid. PDB code 1UBQ (ubiquitin).

We previously reported the successful application of the auxiliary 2-aminooxyethanethiol towards peptide ubiquitylation^13^. The utility of this auxiliary group lies in its high-yielding 3-step synthesis and easy incorporation in various peptide substrates. However, two challenges in removing the auxiliary and producing a wild-type amide linkage were the requirement for pH 3, and the necessity of chaotropes such as 6 M guanidinium chloride that unfold ubiquitin and allow reduction of the N–O bond by metallic Zinc (Fig. 1). Although such a strategy is compatible with proteins that may be refolded from the denatured state, its broad utility is limited. Moreover, the electrophilic character of the nascent disubstituted amide bond in the ligation product led to a small amount of hydrolysis over time, which was exacerbated at the low pH required for efficient N–O bond reduction.

**Figure 2:**
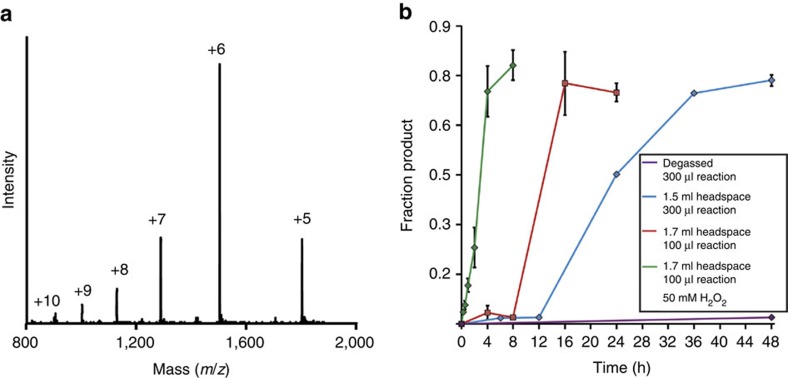
N–O bond cleavage in the native chemical ligation product KAK^Ub(aux)^I. (**a**) ESI-MS spectrum of the final ligation product of ubiquitin(1–75)-α-thioester with KAK^aux^I. Calculated for KAK^Ub(aux)^I, 9,081.1 Da. Observed KAK^Ub^I, 9,004.8±2.7 Da. (**b**) Time-course of N–O bond cleavage and KAK^Ub^I formation from the auxiliary-containing test substrate KAK^Ub(aux)^I in a buffer consisting of 200 mM MPAA, 100 mM NaH_2_PO_4_ at pH 7.3 under the indicated conditions. Error bars represent the s.d. from three independent measurements.

In an effort to reduce the amount of hydrolysed ubiquitin(1–75)-COOH side-product and to increase the rate of transthioesterification between auxiliary-bearing peptides and the ubiquitin(1–75)-α-thioester, we tested the aromatic thiol 4-mercaptophenylacetic acid (MPAA) as a ligation additive (Fig. 1). The excellent leaving group ability of MPAA renders its protein thioesters more reactive towards transthioesterification, the first and rate-limiting step in native chemical ligation^14^,^15^. To our surprise, in a typical ligation reaction with 0.5 mM ubiquitin(1–75)-α-thioester and 5 mM of auxiliary-bearing peptide (KAK^aux^I) in a buffer consisting of 50 mM Tris, 150 mM NaCl and 200 mM MPAA at pH 7.3, we observed the final ligation product to be altogether missing the ligation auxiliary (Fig. 2a and Supplementary Figs 1–4). This unexpected result was consistently reproducible, although the slow kinetics of product formation necessitated up to 48 h to achieve 50–70% yields (Fig. 2b). Additional controls revealed MPAA to be the critical component required for N–O bond cleavage, and re-purification of the commercial compound by high-performance liquid chromatography (HPLC) did not inhibit the reaction (Supplementary Table 1, entries 1–8 and Supplementary Fig. 5). The necessity of a free –SH group in MPAA was seen from the fact that pure disulfide-linked MPAA dimer did not undertake N–O bond cleavage (Supplementary Fig. 6 and Supplementary Table 1, entry 9).

### N–O bond reduction by aromatic and aliphatic thiols

In order to ascertain the generalizability of this unprecedented N–O bond cleavage reaction, we tested a range of aromatic and aliphatic thiols with the ubiquitylated ligation product KAK^Ub(aux)^I, bearing the auxiliary at the site of ligation (Supplementary Fig. 7). With the exception of 4-hydroxythiophenol, all aromatic thiols tested undertook N–O bond cleavage (Table 1, entries 1–5). Interestingly, 4-hydroxythiophenol inhibited MPAA-mediated N–O bond cleavage when equal amounts of both were present in the reaction mixture. In contrast, none of the aliphatic thiols led to N–O bond reduction under identical buffer conditions (Table 1, entries 6–10). This suggests that near a neutral pH the N–O bond is compatible with aliphatic thiol additives commonly employed in native chemical ligations, such as 2-mercaptoethanesulfonic acid (MESNa), and that it is also stable to the biological reducing agent glutathione.

One key difference between the two compound classes tested is that the aromatic thiols are >50% deprotonated at pH 7.3, while the aliphatic thiols are largely protonated. The importance of a deprotonated thiolate species was suggested by the fact that N–O bond cleavage by MPAA was dramatically reduced at pH 6.0 (Supplementary Table 1, entries 10–12).

### Mechanistic investigation of the N–O bond cleavage reaction

Examples of bioactive compounds with chemically labile N–O bonds include pro-drug forms of the duocarmycin and CC-1065 class of antitumor agents^16^. These were proposed to undergo conversion to a biologically active form upon cleavage of the N–O bond by nucleophilic thiols within the tumor microenvironment. Since we observed an increase in N–O bond cleavage with increasing pH, a nucleophilic mechanism may in principle also be invoked for the aromatic thiols. However, freeze-thaw degassing the reaction mixtures under an Argon atmosphere sufficed to inhibit the reaction with MPAA at pH 7.3–8.5 (Fig. 2b and Supplementary Table 1, entry 13–14), ruling out a purely nucleophilic mechanism and suggesting a key role for dissolved oxygen. In support of the latter, increasing the reaction headspace, and thereby the ratio of molecular oxygen to thiol, resulted in halving the reaction time to 24 h (Fig. 2b).

Molecular oxygen may act as a terminal electron acceptor and favour the formation of aromatic thiyl radicals from aromatic thiolates^17^. One indication that thiyl radicals may be present under the reaction conditions was our observation of the oxidized MPAA disulfide species. To test the possibility that aromatic thiyl radicals are spontaneously formed in buffered aqueous solutions at pH 7.3, we incubated a range of aromatic thiols with the co-factor nicotinamide adenine dinucleotide (NADH) (Supplementary Fig. 8). Thiyl radicals react with NADH to yield the NAD^·^ radical and the consumption of NADH is readily detected by a decrease in absorbance at 340 nm (ref. 18). In buffers that favoured N–O bond reduction, we also observed a dramatic decrease in NADH concentration. Importantly, and consistent with their inability to reduce the N–O bond, we did not observe similar oxidation of NADH in reactions with aliphatic thiols 6–10 in Table 1 over a 6 h time-course. The disparity in reducing nature of aromatic and aliphatic thiols was further seen by their reaction with methyl viologen (MV^2+^). A rapid increase in absorption at 605 nm characteristic of the single-electron transfer reduction product MV^+^ was observed only with aromatic thiols, suggesting the formation of a strongly reducing species in solution^19^.

**Figure 3:**
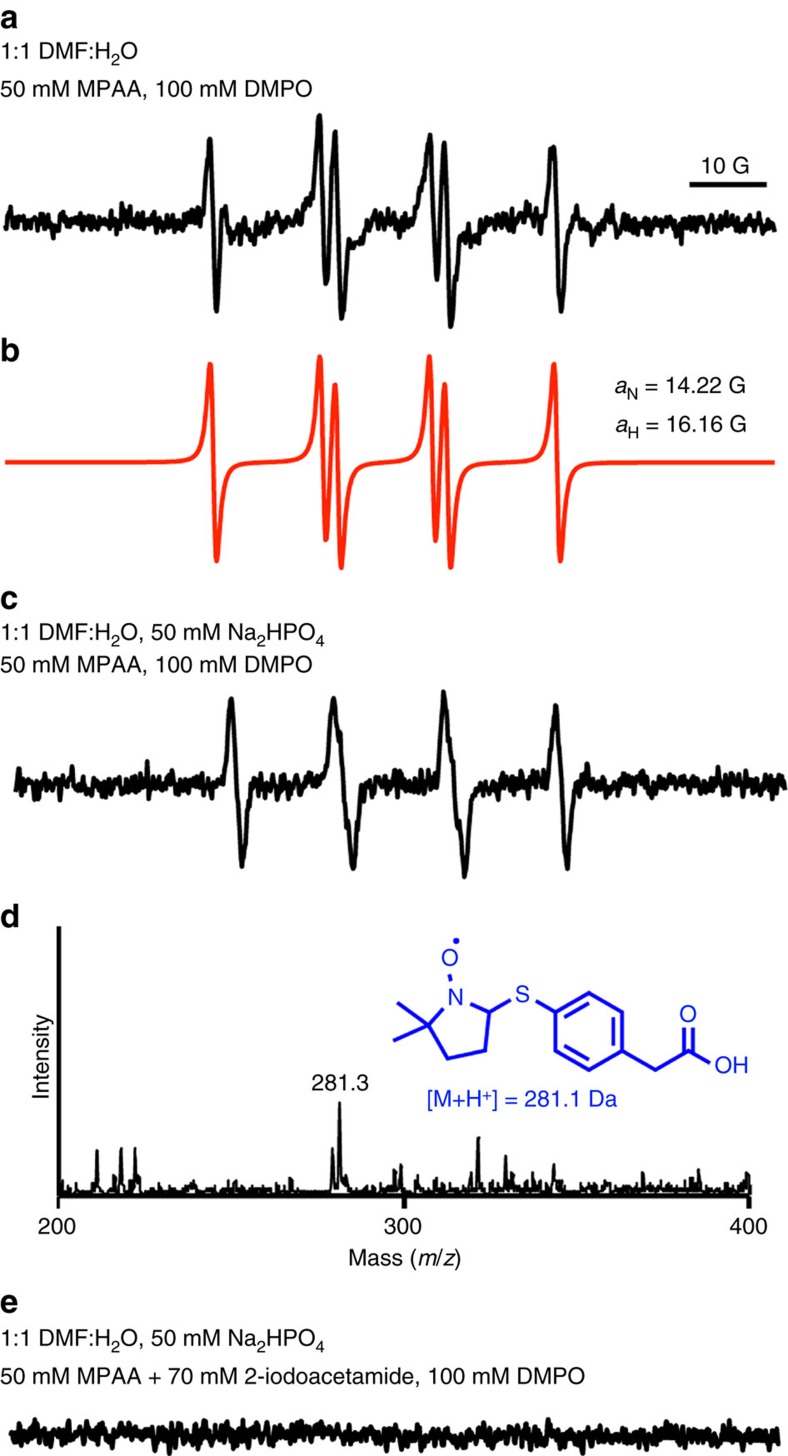
EPR spectra of DMPO/·S-Ar adduct. (**a**) Spectrum obtained upon incubating 50 mM MPAA and 100 mM DMPO in a 1:1 water-DMF mixture at 25 °C. (**b**) Computer simulation of the spectrum observed in **a** with hyperfine splitting constants *a*_N_=14.22 G and *a*_H_=16.16 G^23^. (**c**) Spectrum obtained upon incubating 50 mM MPAA and 100 mM DMPO in 50 mM Na_2_HPO_4_ at pH 7.5, in a 1:1 water-DMF mixture at 25 °C. (**d**) ESI-MS spectrum obtained by LC-ESI-MS analysis of the reaction components in **c**. Inset shows the proposed radical adduct. (**e**) EPR spectrum obtained upon pre-incubating 50 mM MPAA with 70 mM 2-iodoacetamide for 1.5 h followed by 100 mM DMPO in 50 mM Na_2_HPO_4_ at pH 7.5, in a 1:1 water-DMF mixture at 25 °C. Incubation of 50 mM MPAA and 100 mM DMPO in 50 mM Na_2_HPO_4_ at pH 7.5, in a 1:1 water-DMF mixture at 25 °C for 1.5 h without the addition of 2-iodoacetamide resulted in a spectrum similar to that seen in **c**. Spectrometer settings: microwave power, 20 mW; modulation amplitude, 1.0 G; time constant, 163 ms; scan rate, 0.6 G/s.

Electron paramagnetic resonance (EPR) is a widely employed technique to detect species with unpaired electrons. However, thiyl radicals are generally not directly detectable by EPR in solution due to the large spin-orbit coupling constant of sulfur, which leads to fast relaxation of the electron spin^20^. Hence we attempted indirect EPR detection of the MPAA radical in solution at room temperature by spin-trapping with the compound 5,5-dimethyl-1-pyrroline-*N*-oxide (DMPO)^21^. To our delight, we observed the formation of nitroxide radicals in a solution containing 50 mM MPAA and 100 mM DMPO dissolved in 50% (v/v) aqueous *N*,*N*-dimethylformamide (DMF) (Fig. 3a). This was attributed to the addition of a thiyl radical into DMPO. The spectrum exhibited six lines characteristic of DMPO-trapped thiyl radicals^22^, which could be simulated with *a*_N_=14.22 G and *a*_H_=16.16 G (Fig. 3b), giving *a*_N_<*a*_H_ as expected for DMPO-thiyl radical adducts in aqueous solution^23^,^24^. The inclusion of 50 mM Na_2_HPO_4_, pH 7.5 in the reaction altered the appearance of the EPR spectrum (Fig. 3c), but controls lacking MPAA did not show EPR signal under these conditions (Supplementary Fig. 9). Furthermore, trapping of the MPAA thiyl radical was confirmed by electrospray ionisation mass spectrometry (ESI-MS) (Fig. 3d and Supplementary Fig. 10). Importantly, alkylation of MPAA *in situ* with 70 mM 2-iodoacetamide for 1.5 h precluded the appearance of an EPR signal, providing further evidence for a thiyl radical as the reactive species (Fig. 3e). In support of MPAA thiyl radical formation in the presence of 50 mM Na_2_HPO_4_, we recapitulated the spectrum observed in Fig. 3c by adding a small volume of 600 mM Na_2_HPO_4_ to a final concentration of 50 mM in the 50% (v/v) aqueous DMF mixture (Supplementary Fig. 11). Next, we included 50 mM H_2_O_2_ in the solution containing 50 mM MPAA and 100 mM DMPO dissolved in 50% (v/v) aqueous DMF. We observed a spectrum identical to that without H_2_O_2_ present, yet with much greater signal intensity, and again observed the mass of the DMPO-MPAA adduct (Supplementary Fig. 12a). However, we did not see any significant nitroxide radicals forming in the absence of MPAA, and any DMPO-OH adduct expected to arise from H_2_O_2_ alone was only observable by ESI-MS (Supplementary Fig. 12b). Finally, if the MPAA radical is crucial for N–O bond cleavage, we expected its trapping by DMPO to inhibit the reaction. Indeed, we found that the addition of 1 M DMPO to a reaction containing 100 mM MPAA inhibited product formation over the course of 24 h.

One potential pathway for radical-mediated N–O bond cleavage is by formation of a thiyl radical in the auxiliary (Supplementary Fig. 13). A 1,3-sigmatropic rearrangement of the thiyl radical would result in a carbon-centered radical adjacent to the low energy N–O bond, which favours its homolysis^25^. In order to test this mechanism we alkylated the auxiliary thiol in the ligation product with *N*-(2-chloroethyl)-*N,N*-dimethylammonium chloride (Supplementary Fig. 14), thereby precluding formation of a thiyl radical. Upon treatment with 200 mM MPAA at pH 7.3 we still observed efficient N–O bond cleavage in the *S*-alkylated product, indicating that a substrate-derived thiyl radical is not essential for the reaction to proceed. An alternative pathway for N–O bond cleavage is direct reduction by a reducing agent. In thinking of reducing species that are generated by a combination of aromatic thiolates and thiyl radicals, we considered the possibility of a disulfide radical anion. The formation of this high-energy species has been observed with small molecule thiols such as cysteine and glutathione^26^, and in the active site of the enzyme ribonucleotide reductase^27^. The presence of an unpaired electron in an antibonding σ* orbital renders the disulfide radical anion a strongly reducing yet transient species, in equilibrium with the dissociated radical and thiolate forms^28^.

We surmised that slow formation of the thiyl radical by molecular oxygen in the absence of added radical initiators along with the transient nature of the disulfide radical anion together contribute to the slow kinetics of N–O bond cleavage. An initial attempt to increase the rate of MPAA-mediated N–O bond cleavage by including the water-soluble radical initiator, 2,2'-azobis[2-(2-imidazolin-2-yl)propane]dihydrochloride (VA-044) in our reactions proved unfruitful. This is likely due to the rapid quenching of the carbon-centered radical before the formation of significant amounts of the disulfide radical anion and is consistent with the reported inhibition of radical-mediated desulfurization of cysteine residues by MPAA, which also employs VA-044 (ref. 29).

We also considered if superoxide, formed en route to the thiyl radical, may act as a reducing agent^30^. To test this possibility, we utilized the well established superoxide generating system consisting of xanthine oxidase and its substrate hypoxanthine^31^. xanthine oxidase catalyses the conversion of hypoxanthine first to Xanthine then to uric acid, and superoxide is released at each step. We performed this reaction in the presence of KAK^Ub(aux)^I, with hypoxanthine at 4-fold excess relative to the auxiliary containing test substrate. By monitoring the appearance of uric acid at 290 nm, we observed that all of the hypoxanthine was converted to uric acid within the first minute of reaction, producing a burst of superoxide (Supplementary Fig. 15). However, even after 24 h no cleavage of the auxiliary was observed, strengthening our hypothesis that a disulfide radical anion is the likely reductant (Supplementary Table 2, entry 1).

**Figure 4:**
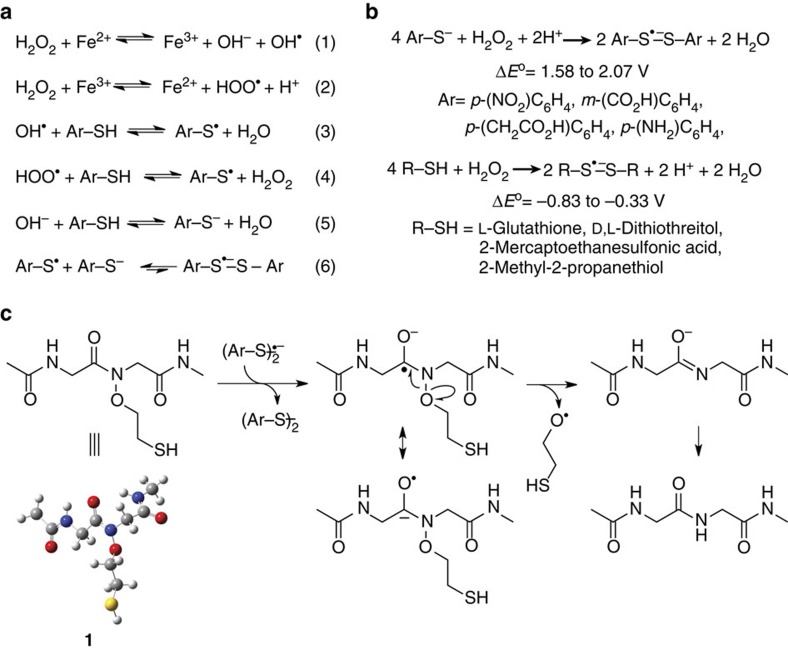
Formation of disulfide radical anions and their role in N–O bond cleavage. (**a**) Production of aromatic thiyl radicals mediated by trace-metal-catalysed Fenton chemistry (1–4) and their combination with aromatic thiolates to form disulfide radical anions (5–6). (**b**) Net chemical equations for the formation of disulfide radical anions from aromatic thiolates and aliphatic thiols at pH 7.3. The calculated range of standard redox potentials is indicated for compounds from each class of molecules. (**c**) Proposed mechanism for disulfide radical anion-mediated N–O bond cleavage in the model compound **1**.

Known methods to generate disulfide radical anions from thiols or disulfides in solution include flash photolysis^28^, pulse radiolysis^32^ or cyclic voltammetry^33^, all of which are technically challenging in the presence of folded protein substrates. Therefore, we wondered if a mild oxidant, such as hydrogen peroxide (H_2_O_2_), would facilitate the formation of thiyl radicals by Fenton chemistry (Fig. 4a)^34^,^35^. Indeed, the inclusion of 50 mM H_2_O_2_ with 200 mM MPAA led to a significant increase in the rate of product formation, requiring only 4 h to attain maximal conversion with no detectable amounts of undesired protein oxidation (Fig. 2b and Supplementary Table 2, entry 2). The chelation of free metal ions with 50 mM EDTA effectively inhibited the reaction, indicating the key role for trace metal ions in generating thiyl radicals (Supplementary Table 2, entry 3). Since the concentration of trace metal ions in reaction components may vary, we demonstrated that 1 mM FeCl_2_ may be added to facilitate the reaction with no deleterious effect on reaction yield (Supplementary Table 2, entry 4). Importantly, 50 mM H_2_O_2_ alone or mixed with 1 mM FeCl_2_ did not yield detectable product in the absence of MPAA, proving that Fenton chemistry alone cannot undertake N–O bond cleavage (Supplementary Table 2, entries 5–6). Finally, freeze-thaw degassing a solution of 50 mM H_2_O_2_ and 200 mM MPAA failed to prevent reductive chemistry, which confirmed our hypothesis that H_2_O_2_ can favour thiyl radical formation even in the absence of molecular oxygen (Fig. 4a and Supplementary Table 2, entry 7).

### Computational studies of N–O bond cleavage

We next undertook *ab initio* quantum chemistry calculations to interrogate the feasibility of disulfide radical anion formation and its reactivity towards the N–O bond. The relative redox potentials (Δ*E*°) for (1) electron transfer between various thiols and hydrogen peroxide (Fig. 4b), and (2) subsequent electron transfer between the disulfide radical anion and a model diglycine compound, **1** (Fig. 4c), were computed. Δ*E*° values for the first step were obtained by calculating the free energy changes of the redox reactions using the Gaussian 09 program package^36^. Equilibrium geometries of all species were located via geometry optimization and the thermal corrections were evaluated at the B3LYP/6-31G* level of theory, with the solvent effect modelled using the polarizable continuum model. The electronic energies at the equilibrium geometries were computed at the B3LYP/6-311++G** level of theory and the results are summarized in Table 2.

Our calculations revealed that protonated aliphatic thiols do not favour disulfide radical anion formation (Table 2, entries 5–8). In contrast, deprotonated aromatic thiolates can be oxidized by hydrogen peroxide to form disulfide radical anions (Table 2, entries 1–4). For MPAA, the calculated Δ*E*° of 2.07 V (Table 2, entry 3) indicates that disulfide radical anion formation is thermodynamically favoured. However, the rate of formation is not predictable by computation and disulfide radical anions are known to exist in an equilibrium that favours the dissociated radical and thiolate species^27^, which likely underlies the slow kinetics of reduction. Next, we focused on electron transfer from the MPAA disulfide radical anion to **1** (Fig. 4c). Based on the calculated electron densities in the highest occupied molecular orbital of the disulfide radical anion and the lowest unoccupied molecular orbital in the diglycine peptide (Supplementary Fig. 16) two possible pathways exist. We found both pathways to be thermodynamically permissible, with Δ*E*° of 0.53 V for the production of a β-mercaptoethyloxyl radical, and Δ*E*° of 0.04 V for the production of the β-mercaptoethanolate anion. When up to nine explicit H_2_O molecules in varying combinations were included in these calculations, most instances within the test set favoured the β-mercaptoethanolate anion (Supplementary Table 4). Finally, we also examined the thermodynamics of direct electron transfer from the MPAA thiolate to **1** and found this process to be energetically unfavourable, which suggests that the thiolate form alone cannot act as a reductant.

### Mechanistic studies with a model diglycine compound

In order to unambiguously identify the β-mercaptoethanol predicted by our proposed mechanism (Fig. 4c), we synthesized the *S*-trityl-protected form of the model dipeptide, compound **2** (Supplementary Figs 17 and 18). Compound **2** was sparingly soluble in water and hence subjected to reduction with 200 mM MPAA in a buffer consisting of 100 mM Na_2_HPO_4_, pH 7.3 in 50% (v/v) aqueous DMF. Consistent with results obtained from protein substrates, and the detection of a MPAA radical under these conditions, we observed cleavage of the N–O bond over 24 h. The *S*-trityl-protected β-mercaptoethanol was isolated and confirmed by NMR (Supplementary Fig. 19). Surprisingly, in the presence of 50 mM H_2_O_2_ and 200 mM MPAA, complete N–O bond cleavage in 20 mM of **2** was observed in 10 min. This may reflect the greater accessibility of the labile bond in **2** than in ubiquitylated peptides, or more productive electron transfer arising from a smaller number of competing amide bonds than in ubiquitin. That trityl protection of the auxiliary thiol did not prevent N–O bond cleavage underscores the fact that electron transfer to the N–O bond occurs directly from the reducing species. As expected, sequestration of trace metal ions by treatment of the buffer with a metal-chelating resin also inhibited N–O bond cleavage in the model compound (Supplementary Table 3).

### One-pot strategy for native chemical ubiquitylation

Current chemical ubiquitylation methodologies are not optimal for native folded proteins^29^,^37^. Therefore, an efficient one-pot method to perform native ubiquitylation is highly desirable. With this in mind, we sought to improve the yield of one-pot auxiliary-mediated ubiquitylation by limiting the formation of ubiquitin(1–75)-COOH. Slow hydrolysis of both the ubiquitin(1–75)-α-thioester and the disubstituted amide in the ligation product contribute to this undesired side-product, which can in principle be alleviated by enhancing the kinetics of both ligation and N–O bond reduction. Surprisingly, we found that conducting the ligation reaction without MPAA significantly avoided ubiquitin(1–75)-COOH formation by preventing premature auxiliary removal in the starting materials. The subsequent addition of 200 mM MPAA to the crude ligation mixture and incubation for an additional 24 h at 25 °C generated the final reduced ubiquitylated peptide in 82% overall yield. This represents a 20% higher yield over reactions where MPAA was added at the start of ligation.

### Synthesis of full-length sumoylated histone H4

As an initial test of the auxiliary's utility in the context of folded proteins, we incorporated non-denaturing MPAA-mediated auxiliary removal into the semisynthesis of full-length sumoylated human histone H4 (suH4). Although histone sumoylation was first reported over a decade ago, very little is known regarding its functional role in human chromatin^38^. Access to quantities of H4 site-specifically conjugated with the C terminus of SUMO-3 at Lys12 is crucial for biochemical investigations of the role for sumoylation in regulating chromatin structure and function^39^. We devised a synthetic strategy for suH4 (Supplementary Fig. 20 and Supplementary Methods). The H4 (1–14) peptidyl hydrazide was synthesized using Fmoc chemistry on the solid phase, with Gly92 of SUMO-3 and the ligation auxiliary attached to Lys12 (Supplementary Fig. 21). Following release from the solid-phase and global deprotection, the peptide was ligated to the SUMO-3(2–91)C47S-α-thioester to yield the sumoylated peptide (Supplementary Figs 22 and 23). One key challenge we anticipated was conversion of the hydrazide to a thioester without cleaving the N–O bond. However, we found the auxiliary to be completely stable to both diazotization and thioester formation, which employed NaNO_2_ at pH 3.0 followed by displacement of the resulting azide with 100 mM MPAA^40^. Complete retention of the auxiliary through these steps highlights its utility in diverse native chemical ligation strategies. The sumoylated H4 peptide α-thioester was then reacted with a truncated H4(15–102) protein containing the A15C mutation at its N terminus to facilitate native chemical ligation (Supplementary Fig. 24). Ligation proceeded over 24 h to afford 2.1 mg of the ligated product, retaining the ligation auxiliary, in 66% purified yield (Supplementary Fig. 25). The ligation product was then dissolved in a buffer consisting of 100 mM Na_2_HPO_4_, 200 mM MPAA, pH 7.3 and N–O bond cleavage allowed at 25 °C over 24 h to yield the reduced compound (Supplementary Fig. 26). Importantly, Cys15 in H4 was unaffected by MPAA-mediated auxiliary removal, demonstrating the compatibility of this reaction with folded proteins containing Cys residues. In the terminal step, the full-length sumoylated histone H4 A15C mutant was desulfurized to yield the desired suH4 in 41% yield over the last two steps (Supplementary Fig. 27).

### Synthesis of full-length sumoylated histone H2B

The ultimate goal for our chemical strategy is complete compatibility with native folded proteins containing Cys residues. We envision future applications wherein a suitably protected ligation auxiliary is directly incorporated in target proteins by employing an amber suppression strategy and purified from producer strains before native ubiquitylation/sumoylation^41^. Therefore, having demonstrated that MPAA can mediate auxiliary removal from sumoylated histone H4 under non-denaturing conditions, we sought to perform (1) auxiliary deprotection, (2) sumoylation and (3) the auxiliary removal step on an additional protein target without intermediate denaturation and purification steps. We were particularly attracted to histone H2B as it is sumoylated at its C-terminal Lys120 (suH2B; ref. 42) and genetic experiments suggest that sumoylation recapitulates the genomic occupancy of H2B Lys120 ubiquitylation^43^. However, similar to suH4, the role of suH2B in chromatin regulation awaits *in vitro* biochemical investigation.

**Figure 5:**
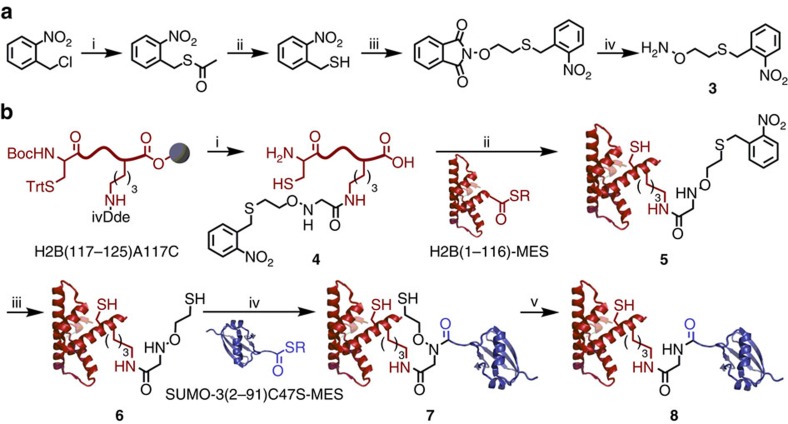
Semisynthesis of full-length sumoylated histone H2B(A117C). (**a**) Synthesis of photoprotected auxiliary **3**. (i) CH_3_C(O)SH, K_2_CO_3_, THF, 8 h, 25 °C. (ii) HCl, CH_3_OH, 6 h, 60 °C, 75% (2 steps). (iii) *N*-(2-bromoethoxy)phthalimide, Et_3_N, DMSO, 4 h, 25 °C, 74%. (iv) H_2_NNH_2_, CHCl_3_, 1 h, 25 °C, 98%. (**b**) (i) Site-specific coupling of **3** to H2B(117–125)A117C Lys120 followed by acidolytic release of the unprotected peptide, **4**, from the solid-phase. (ii) Expressed protein ligation of **4** with H2B(1–116)-α-thioester to generate full-length H2B(A117C) with protected auxiliary at Lys120, **5**. (iii) photolytic removal of the auxiliary protecting group to give H2B(A117C) with unprotected auxiliary at Lys120, **6**. (iv) Expressed protein ligation of **6** with SUMO-3(2–91)C47S-α-thioester to generate sumoylated H2B(A117C) **7**, with retention of the ligation auxiliary. (v) Selective removal of the ligation auxiliary with 150 mM MPAA under non-denaturing conditions to yield sumoylated H2B(A117C) **8**. ivDde=1-(4,4-Dimethyl-2,6-dioxocyclohexylidene)-3-methylbutyl group. PDB codes, 1KX5 (H2B) and 1U4A (SUMO-3).

**Figure 6:**
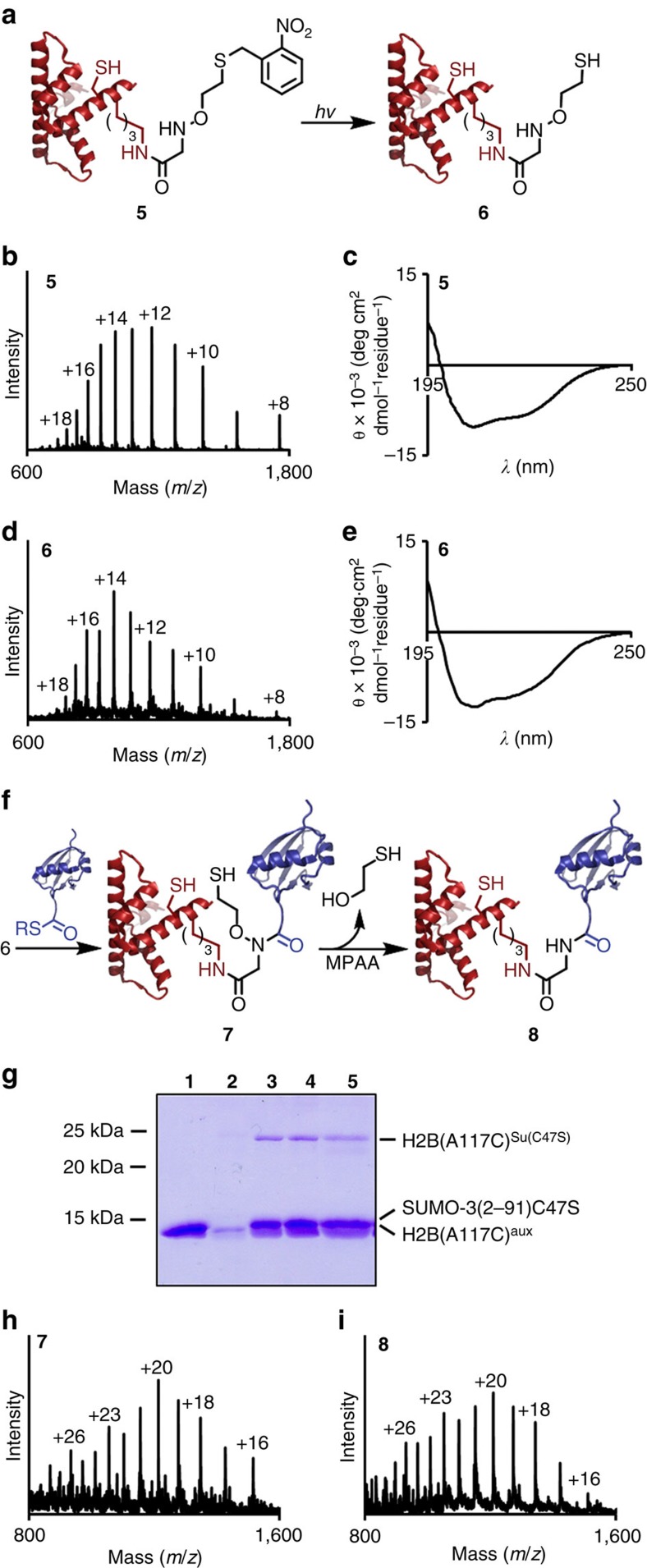
Photodeprotection and sumoylation of folded histone H2B. (**a**) Scheme depicting photolytic cleavage of the auxiliary protecting group from H2B(A117C)^photoaux^ (**5**) to generate H2B(A117C)^aux^ (**6**). (**b**) ESI-MS spectrum of **5**. Calculated for **5**, 14,059.2 Da. Observed for **5**, 14,062.7±2.8 Da. (**c**) Circular dichroism spectrum of **5** in 50 mM Na_2_HPO_4_, pH 7.5. (**d**) ESI-MS spectrum of **6**. Calculated for **6**, 13,924.1 Da. Observed for **6**, 13,925.8±2.6 Da. (**e**) Circular dichroism spectrum of **6** in 50 mM Na_2_HPO_4_, pH 7.5. (**f**) Scheme depicting ligation of **6** to SUMO-3(2–91)C47S-α-thioester to yield H2B(A117C)^Su(C47S)aux^ (**7**) and subsequent MPAA-mediated auxiliary removal to yield H2B(A117C)^Su(C47S)^ (**8**) under folded conditions. (**g**) Coomassie-stained 15% SDS–polyacrylamide gel electrophoresis gel of ligation between H2B(A117C)^aux^ (**6**) and SUMO3(2–91)C47S-α-thioester under non-denaturing conditions. Lane 1=SUMO-3(2–91)C47S-MES, 2=H2B(A117C)^aux^, 3=24 h ligation, 4=48 h ligation, 5=24 h MPAA incubation. (**h**) ESI-MS spectrum of the ligation product, H2B(A117C)^Su(C47S)aux^ (**7**). Calculated for **7**, 24,225.6 Da. Observed for **7**, 24,230.9±4.0 Da. (**i**) ESI-MS spectrum of the final product, H2B(A117C)^Su(C47S)^ (**8**). Calculated for **8**, 24,149.6 Da. Observed for **8**, 24,153.1±3.2 Da.

Towards the semisynthesis of suH2B, we first generated full-length histone H2B bearing a protected ligation auxiliary as the entry point for testing our methodology. To ensure that the auxiliary protecting group could be removed under native conditions, we synthesized a photoprotected form (**3**) by starting from 2-nitrobenzyl chloride and *N*-(2-bromoethoxy)phthalimide (Fig. 5a and Supplementary Figs 28–30)^13^. The auxiliary **3** was incorporated at Lys120 of the H2B(117–125) C-terminal peptide with an Ala to Cys mutation at position 117 (Fig. 5b). After acidolytic cleavage from the solid phase, the peptide **4** was ligated via its N-terminal Cys to an H2B(1–116)-α-thioester to generate full-length H2B(A117C), **5** (Fig. 6a,b and Supplementary Figs 31–33). The product **5** was folded by dialysis into 50 mM Na_2_HPO_4_, pH 7.5 (Fig. 6c), and every subsequent step was performed under folded conditions. First, complete deprotection of the auxiliary thiol was achieved by irradiation with 365 nm light for 3.5 h in the presence of ascorbic acid, semicarbazide and dithiothreitol. The integrity and folded state of the deprotected protein were confirmed by ESI-MS and circular dichroism (Fig. 6d,e). The deprotected H2B(A117C)^aux^, **6**, was then ligated under non-denaturing conditions to the SUMO-3(2–91)C47S-α-thioester over 48 h to yield the ligation product H2B(A117C)^Su(C47S)aux^, **7** (Fig. 6f–h and Supplementary Fig. 34). The sumoylated product **7** was subjected to MPAA-mediated auxiliary removal for 24 h, yielding the ligation product lacking the ligation auxiliary, H2B(A117C)^Su(C47S)^, **8**, in 15–30% yield over two steps (Fig. 6i). Importantly, we observed no precipitation of the H2B species throughout these manipulations, and the folded state of the reduced ligation product **8** was confirmed by size exclusion chromatography and circular dichroism (Supplementary Fig. 35). As a functional test of the correct folded state of SUMO-3 in **8**, we undertook its desumoylation with the SUMO-specific protease sentrin specific peptidase 1 (SENP1). Congruent with our observations that MPAA-mediated N–O bond cleavage did not lead to the denaturation or aggregation of **8**, we observed efficient hydrolysis by SENP1 and the appearance of lower molecular weight species corresponding to the hydrolysed SUMO-3 and H2B(A117C) (Supplementary Fig. 36).

## Discussion

The N–O bond is frequently encountered in organic chemistry and several methods exist for the cleavage of this moiety, including TiCl_3_ (ref. 44), catalytic hydrogenation^45^, Na/Hg amalgams^46^ and SmI_2_ (ref. 47). More recently, neutral organic super-electron donors were demonstrated to reduce N–O bonds in Weinreb amides^48^. However, the application of any of these reagents to folded proteins in aqueous buffers is extremely challenging. Our discovery of an unprecedented N–O bond reductive chemistry sets the stage for new applications that would benefit from the controlled reversal of this low-energy bond. The straightforward synthesis of the 2-aminooxyethanethiol auxiliary and its photoprotected form, their facile incorporation into peptides, and cleavage by a subset of readily available water-soluble aromatic thiols is particularly appealing for applications in protein semisynthesis. Both experimental and computational investigations support our hypothesis that N–O bond cleavage may involve the formation of a transient disulfide radical anion species. Multiple observations towards this include the requirement for an oxidant, the necessity for trace metal ions, and the detection of aromatic thiyl radicals. Importantly, our ability to readily control the timing of N–O bond cleavage is particularly appealing as demonstrated by the one-pot synthesis of the ubiquitylated peptide, KAK^Ub^I. As highlighted in our syntheses of the full-length sumoylated human histones H4 and H2B, the extremely mild reductive strategy may also be applied towards the sumoylation, and by extension ubiquitylation, of native folded proteins in aqueous buffers. Indeed, the semisynthesis of suH4 and suH2B will, for the first time, permit detailed biochemical studies of these poorly understood modifications. Finally, having established the stability of the ligation auxiliary to the intracellular reductant glutathione, our immediate future efforts are focused on an amber-codon-suppression strategy to incorporate the auxiliary into natively folded proteins that are inaccessible by fragment-based semisynthetic approaches.

## Methods

### Solid-phase peptide synthesis

All peptides were synthesized by standard 9-fluorenylmethoxycarbonyl (Fmoc)-based solid-phase peptide synthesis on a Liberty Blue Automated Microwave Peptide Synthesizer (CEM, Matthews, NC). The auxiliary containing peptides KAK^aux^I, H4(1–14)^aux^-C(O)NHNH_2_, and H2B(117–125, A117C)^photoaux^-C(O)OH were synthesized on Rink-amide, 2-chlorotrityl chloride, and Wang resin, respectively. The ligation auxiliary was incorporated by coupling bromoacetic acid at the desired Lys ɛ-amine followed by on-resin displacement of the bromide with 0.25 M protected auxiliary, *O*-(2-(tritylthio)ethyl)hydroxylamine or *O*-(2-((2-nitrobenzyl)thio)ethyl)hydroxylamine, in DMSO.

### One-pot ligation and auxiliary removal

The ubiquitin(1–75)-α-thioester (1 eq.) and KAK^aux^I (10 eq.) were dissolved in a reaction buffer containing 50 mM tris, 150 mM NaCl, 10 mM tris(2-carboxyethyl)phosphine (TCEP) pH 7.3, and incubated at 25 °C for 24 h. Following ligation, MPAA was added to a final concentration of 200 mM, and the reaction subsequently incubated at 25 °C for a further 24 h.

### General method for N–O bond cleavage

Auxiliary-containing protein substrates were dissolved in 200 mM MPAA, 100 mM Na_2_HPO_4_, pH 7.3 at a concentration of 0.1 mM. Reactions were either performed in a reaction vessel with headspace at least 15 times that of the reaction volume at 25 °C for 24 h, or supplemented with 50 mM H_2_O_2_ and incubated at 25 °C for 4 h.

### Analysis of auxiliary removal from protein substrates

Reaction mixtures were treated with 50 mM TCEP, pH 7.3, at 4 °C for 30 min, then acidified to pH ∼3 with formic acid. Samples were extracted once with diethyl ether to remove a majority of the aromatic thiol and then analysed by C18 liquid chromatography-electrospray ionization-tandem mass spectrometry (LC-ESI-MS) employing a gradient of 5–100% acetonitrile over 40 min.

### Synthesis of sumyolated H4

Purified SUMO-3(2–91)C47S-MESNa thioester (1 eq.) and H4(1–14)^aux^-C(O)NHNH_2_ (6 eq.) were reacted in a buffer containing 6 M Gn-HCl, 100 mM Na_2_HPO_4_, and 10 mM TCEP, pH 7.3. Ligation proceeded with gentle shaking at 25 °C for 24 h. The ligation product, H4(1–14)^Su(C47S)aux^-C(O)NHNH_2_, was purified by C18 preparative reversed-phase (RP)-HPLC, then converted to an acyl azide by reaction with 15 eq. of NaNO_2_ in 200 mM Na_2_HPO_4_, 6 M Gn-HCl, pH 3.0, at −20 °C for 15 min. A solution of H4(15–102)A15C (2 eq.) in 200 mM Na_2_HPO_4_, 6 M Gn-HCl, 200 mM MPAA, pH 6.5, was then added to the thioester and the mixture was allowed to warm up to room temperature. The pH was adjusted to 6.8–7.0 and ligation allowed at 25 °C for 24 h. The ligation product was purified by C4 semi-preparative RP-HPLC, dissolved in 200 mM MPAA, 100 mM Na_2_HPO_4_, pH 7.3, and incubated at 25 °C for 24 h to remove the auxiliary. Product lacking the auxiliary group was further purified by C4 semi-preparative RP-HPLC and subjected to desulfurization in 280 mM 2-methyl-2-propanethiol, 10 mM VA-044, 100 mM Na_2_HPO_4_, 6 M Gn-HCl, 500 mM TCEP, 100 mM MESNa, pH 7.5. The reaction proceeded at 37 °C for 24 h, and the final desired product was purified by C4 analytical RP-HPLC.

### Synthesis of sumoylated H2B

Purified H2B(1–116)-MESNa thioester (1 eq.) and H2B(117–125, A117C)^photoaux^-C(O)OH (10 eq.) were reacted in a buffer containing 6 M Gn-HCl, 100 mM Na_2_HPO_4_, 10 mM EDTA and 5 mM TCEP, pH 7.5. Ligation proceeded with gentle shaking at 25 °C for 6 h. The ligation product, H2B(A117C)^photoaux^, was purified by C4 preparative RP-HPLC, then folded at 0.25 mg ml^−1^ by dialysis at 4 °C from 6 M Gn-HCl, 100 mM Na_2_HPO_4_, pH 7.5 into 50 mM Na_2_HPO_4_, pH 7.5. Unmasking of the auxiliary thiol was accomplished by adjusting the buffer composition to 50 mM Na_2_HPO_4_, 4 mM semicarbazide, 5 mM ascorbic acid, 0.5 mM dithiothreitol, pH 6–7, and irradiating with 365 nm light for 3.5 h. Deprotected H2B(A117C)^aux^ was dialysed back into 50 mM Na_2_HPO_4_, pH 7.5, and to this solution was added SUMO-3(2–91)C47S-MESNa thioester (3 eq.) dissolved in 50 mM Na_2_HPO_4_, pH 7.5. A solution containing 200 mM TCEP, 20 mM MPAA, 50 mM Na_2_HPO_4_, pH 7.5 was added to the reaction to attain final concentrations of 2 mM TCEP and 0.2 mM MPAA. The ligation proceeded at 22 °C for 48 h. Ligation was confirmed by LC-ESI-MS and SDS–polyacrylamide gel electrophoresis analysis. MPAA was then added to the reaction to a final concentration of 150 mM and the reaction transferred to a container with headspace filled with air equal to 10 times the liquid volume of the reaction. MPAA-mediated auxiliary removal proceeded for 24 h at 22 °C, and the final product, H2B(A117C)^Su(C47S)^, was analysed by LC-ESI-MS, size exclusion chromatography and circular dichroism.

### Thiyl radical detection by oxidation of NADH

To investigate the formation of thiyl radicals under auxiliary removal conditions, reduced NADH was dissolved at a final concentration of 40 mM in solutions containing 200 mM of each thiol and 100 mM Na_2_HPO_4_, pH 7.3. The solutions were incubated at 25 °C, protected from light. Absorbance at 340 nm was measured at various time points. A decrease in absorbance, due to oxidation of NADH, suggested the presence of thiyl radicals^18^. To further confirm the ability of aromatic thiols to perform single-electron transfer reactions, solutions were prepared containing 200 mM of each thiol and 100 mM Na_2_HPO_4_, pH 7.3, and these solutions were added to dry aliquots of the radical indicator, methyl viologen (MV^2+^), for a final concentration of 20 mM MV^2+^. Both MV^2+^ and its two-electron reduction product (MV°) have absorbance maxima less than 400 nm. The single-electron reduction product MV^+^, however, has a strong characteristic absorbance at 605–610 nm (ref. 49). The resulting deep purple colour was observed immediately upon mixing methyl viologen with the aromatic thiol solutions, but no colour change occurred with aliphatic thiols, even after 24 h.

### EPR experiments

A 200 mM MPAA stock solution was prepared in DMF. Aliquots from the stock solution were diluted with DMF and 50 mM Na_2_HPO_4_ at pH 7.5 to a final 1:1 water-DMF mixture. Then, 100 mM DMPO was added, the samples were vortexed, transferred to a flat cell and EPR spectra were recorded at room temperature on a Bruker EMX spectrometer equipped with a high sensitivity cavity and operating at 9.65 GHz and 100 KHz field modulation. MPAA alkylation was performed by incubating 50 mM MPAA with 70 mM 2-iodoacetamide in 50 mM Na_2_HPO_4_ at pH 7.5, in 1:1 water-DMF for 1.5 h at 25 °C before DMPO addition. Parallel controls were also performed by pre-incubating 50 mM MPAA alone in 50 mM Na_2_HPO_4_ at pH 7.5, in a 1:1 water-DMF for 1.5 h at 25 °C before adding DMPO. Computer simulation was performed using the Winsim program from P.E.S.T.^23^.

### SENP1 hydrolysis assay

Size-exclusion-purified H2B(A117C)^Su(C47S)^ (**8**) was assayed with the catalytic domain of sentrin-specific protease 1 (SENP1, Boston Biochem). SENP1 (0.05 nmol) was pre-activated in 10 μl buffer containing 50 mM tris, 150 mM NaCl, 12 mM dithiothreitol, pH 8 for 20 min at 25 °C. To the reduced SENP1 was then added 10 μl of a solution containing 0.5 nmol of **8** in 50 mM tris, 150 mM NaCl, 1 mM dithiothreitol, pH 7.5. The resulting mixture was incubated for 24 h at 37 °C. The assay was quenched by the addition of 6 × Laemmli buffer containing 300 mM dithiothreitol and boiled for 5 min, then run on an 18% SDS–polyacrylamide gel electrophoresis gel at 200 V for 1.5 h and stained with Coomassie brilliant blue.

### Data availability

The data that support the findings of this study are available with the article and its Supplementary Information Files, and from the corresponding author upon reasonable request.

## Additional information

**How to cite this article:** Weller, C. E. *et al*. Aromatic thiol-mediated cleavage of N–O bonds enables chemical ubiquitylation of folded proteins. *Nat. Commun.* **7**,12979 doi: 10.1038/ncomms12979 (2016).

## Supplementary Material

Supplementary Information Supplementary Figures 1-37, Supplementary Tables 1-5. Supplementary Methods and Supplementary References

## Figures and Tables

**Table 1:** Aromatic and aliphatic thiol pKa values and corresponding yields in N–O bond cleavage assays.

**Entry**	**Thiol**	**pKa**^14^,^29^	**Yield (%)**
1	4-Nitrothiophenol	4.5	59
2	3-Mercaptobenzoic acid	5.8	81
3	4-Mercaptophenylacetic acid	6.6	73
4	4-Aminothiophenol	6.9	73
5	4-Hydroxythiophenol	7.0	n.d.
6	2,2,2-Trifluoroethanethiol	7.6	n.d.
7	L-Glutathione	9.1	n.d.
8	D,L-Dithiothreitol	9.2, 10.1	n.d.
9	2-Mercaptoethanesulfonic acid	9.2	n.d.
10	2-Methyl-2-propanethiol	11.2	n.d.

n.d.=no detectable N–O bond cleavage.

**Table 2:** Calculated redox potentials for disulfide radical anion formation from aliphatic and aromatic thiols at pH 7.3.

**Entry**	**Thiol**	**Δ*****E*****° (V)**
1	4-Nitrothiophenol	1.58
2	3-Mercaptobenzoic acid	1.98
3	4-Mercaptophenylacetic acid	2.07
4	4-Aminothiophenol	2.03
5	L-Glutathione	−0.40
6	D,L-Dithiothreitol	−0.33
7	2-Mercaptoethanesulfonic acid	−0.43
8	2-Methyl-2-propanethiol	−0.83
