# Supplementary material for: Aromatic thiol-mediated cleavage of N–O bonds enables chemical ubiquitylation of folded proteins
Source: Nat Commun. 2016 Sep 29;7:12979. doi: 10.1038/ncomms12979 (PMC5056422; doi:10.1038/ncomms12979)
Supplement: Supplementary Information — Supplementary Figures 1-37, Supplementary Tables 1-5. Supplementary Methods and Supplementary References [file ncomms12979-s1.pdf]

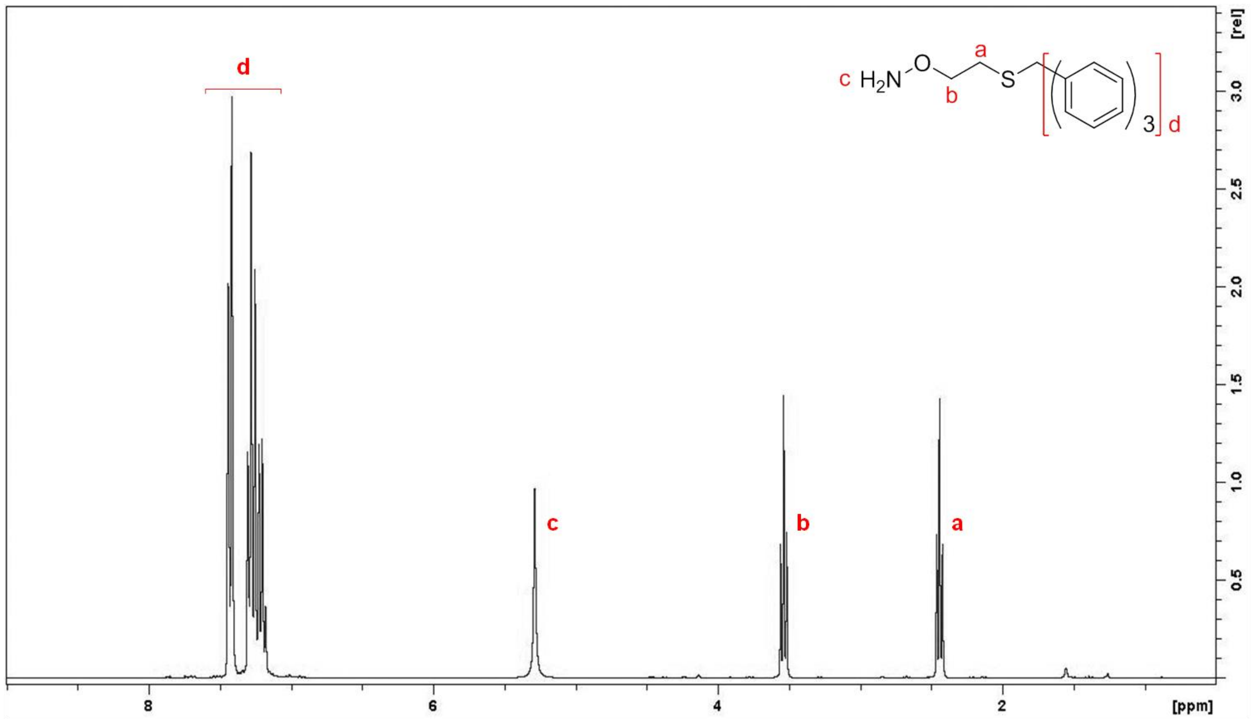

Supplementary Figure 1a. ^{1}H-NMR of *O*-(2-(tritylthio)ethyl)hydroxylamine.

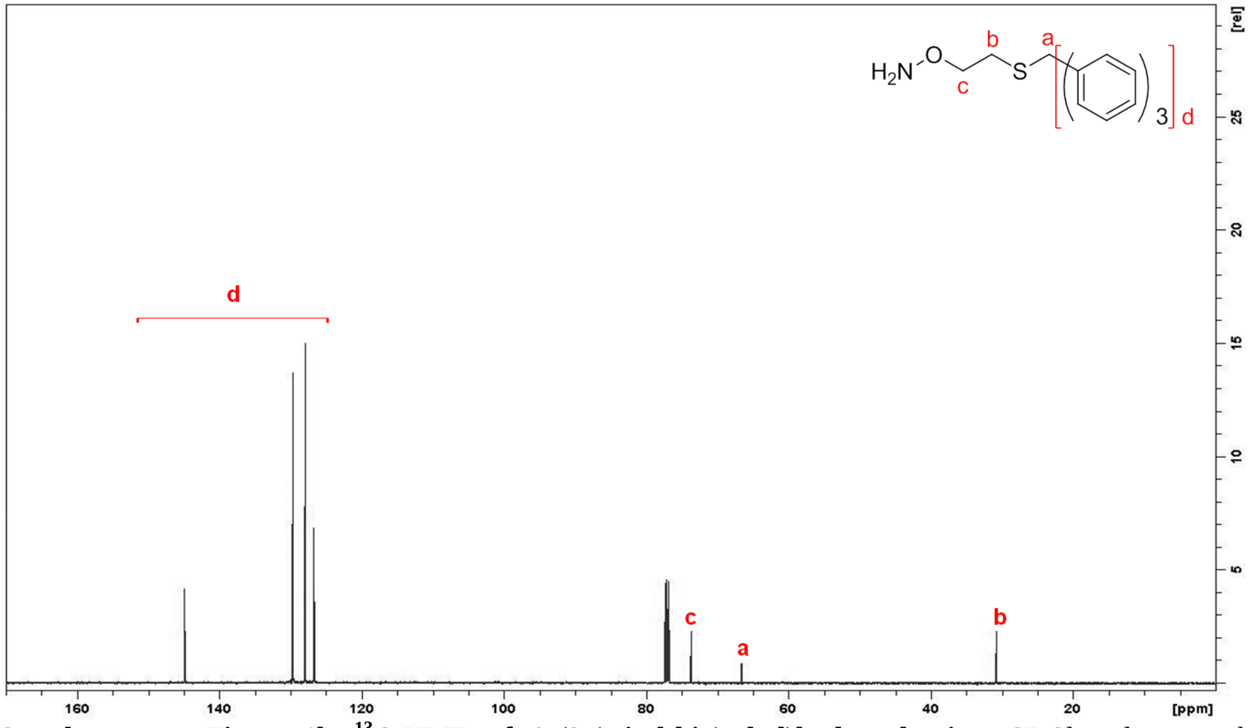

Supplementary Figure 1b. ^{13}C-NMR of *O*-(2-(tritylthio)ethyl)hydroxylamine. $CDCl_3$ solvent peak identified at 77.09 ppm (t).

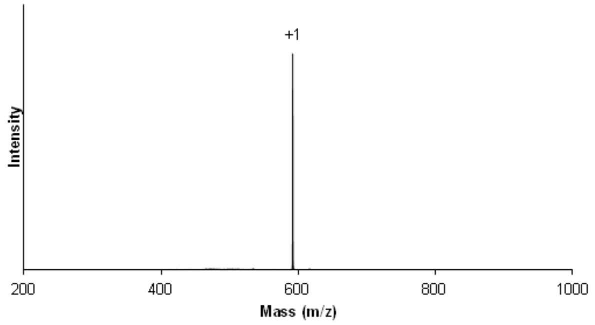

Supplemenatry Figure 2. ESI-MS of purified of KAK^{aux}I.

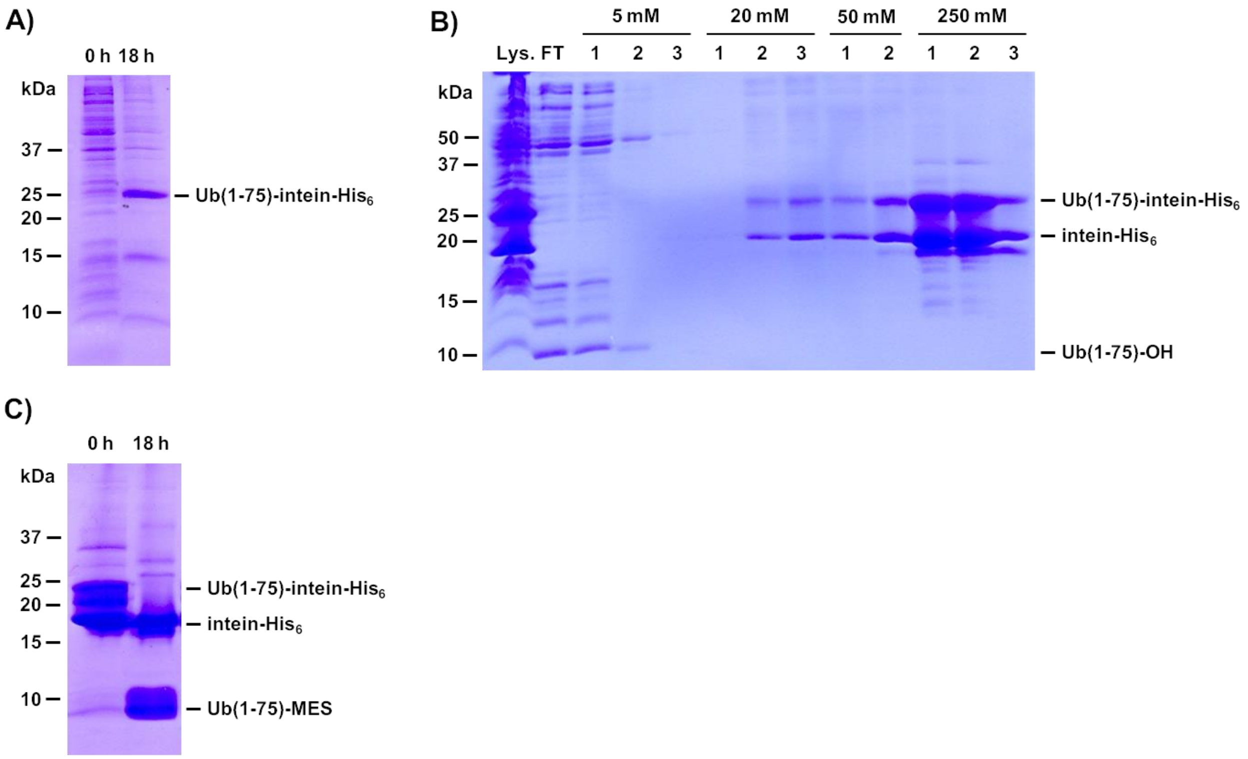

Supplementary Figure 3. Overexpression and purification of Ub(1-75)-MES. Coomassie stained 15% SDS-PAGE gel of (a) IPTG-induced Ub(1-75)-intein-His$_6$ overexpression; (b) Ub(1-75)-intein-His$_6$ purification by Ni^{2+} column; and (c) Thiolysis of pooled fractions containing Ub(1-75)-intein-His$_6$. Lys = cell lysate. FT = column flow-through after protein binding.

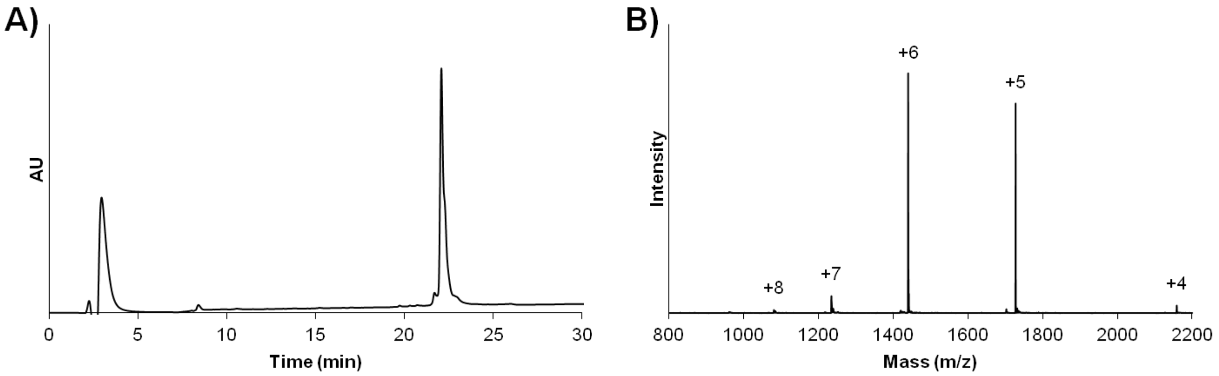

Supplementary Figure 4. Purification of Ub(1-75)-MES. (a) C18 analytical RP-HPLC chromatogram of purified Ub(1-75)-MES, gradient of 0-73% B, 30 min. (b) ESI-MS of purified Ub(1-75)-MES.

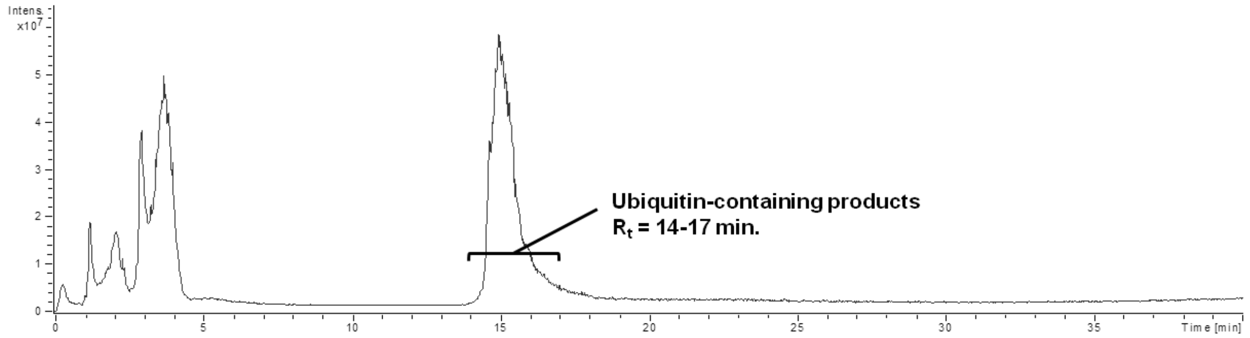

Supplementary Figure 5. Sample LC-ESI-MS trace of an N-O bond cleavage test. Typical total ion chromatogram of an N-O bond cleavage test on $KAK^{Ub(aux)}I$ substrate. The sample is injected on a gradient of 0-100% D over 40 min. Ubiquitin-containing species co-elute between 14 and 17 min. ESI-MS signal is averaged over 14-17 min, and this spectrum used to quantify yield.

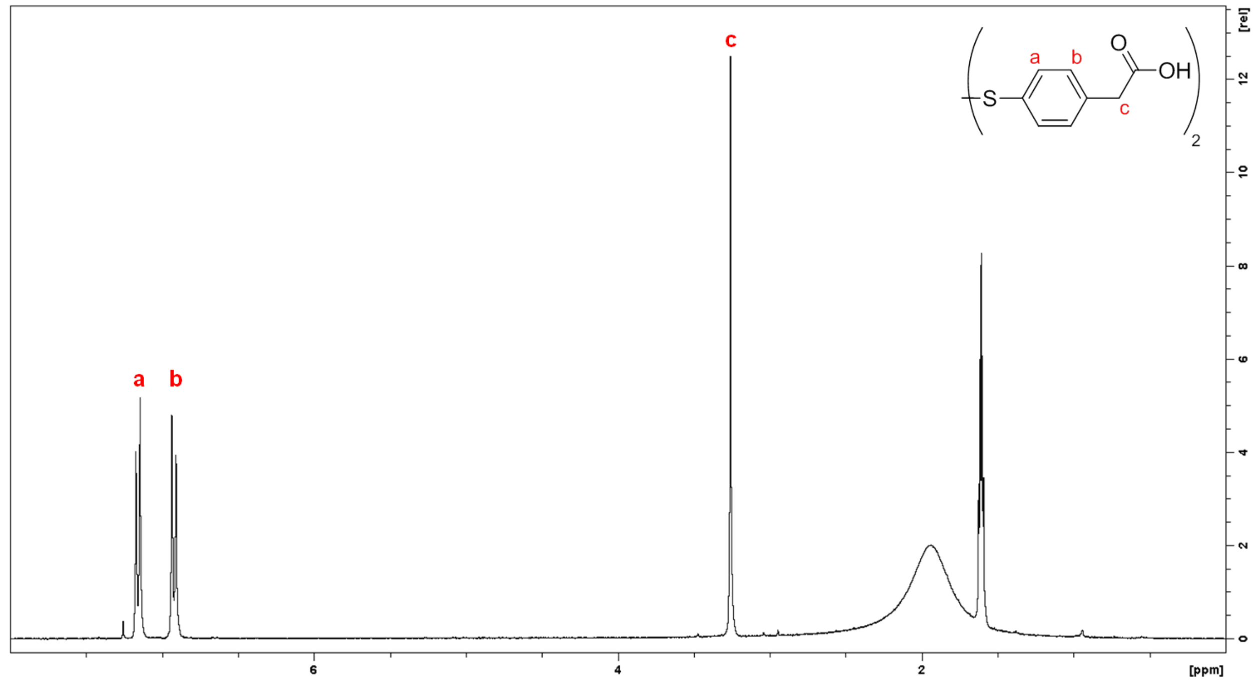

Supplementary Figure 6. ^{1}H-NMR of the disulfide-linked MPAA dimer 2,2'-(disulfanediylbis(4,1-phenylene))diacetic acid. Peak at 1.95 ppm (s) identified as H_2O. CD_3CN solvent peak observed at 1.61 ppm (m).

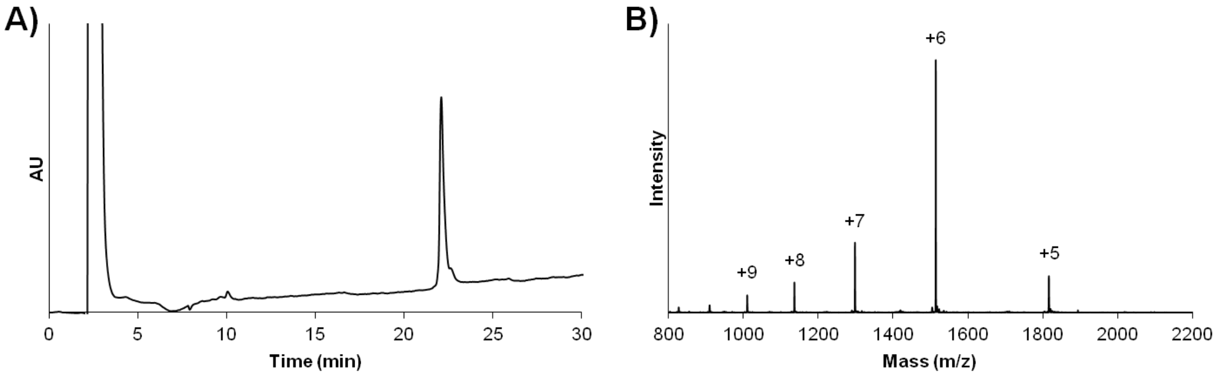

Supplementary Figure 7. Purification of KAK$^{Ub(aux)}$I. (a) C18 analytical RP-HPLC chromatogram of purified KAK$^{Ub(aux)}$I, gradient of 0-73% B, 30 min. (b) ESI-MS of purified KAK$^{Ub(aux)}$I.

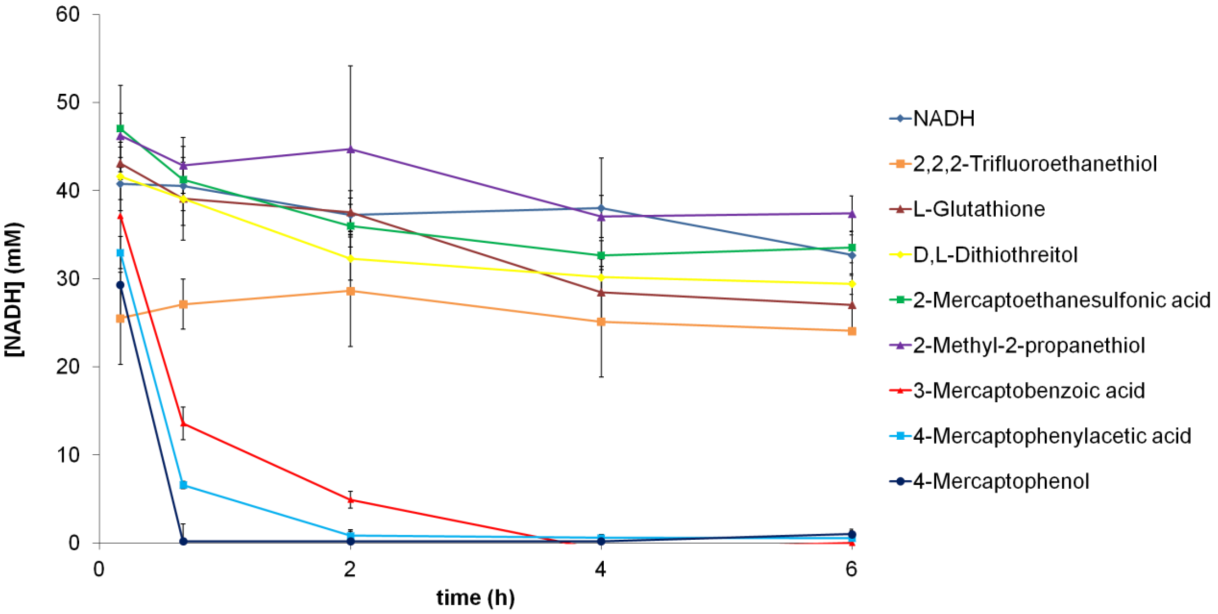

Supplemenatry Figure 8. Time course of NADH oxidation by aromatic and aliphatic thiols. NADH was dissolved at 40 mM in a solution of 100 mM Na_2HPO_4, pH 7.3, containing 200 mM of the indicated thiol. NADH was detected by absorbance at 340 nm. NADH concentration at each time point was calculated from a standard curve of NADH in buffer.

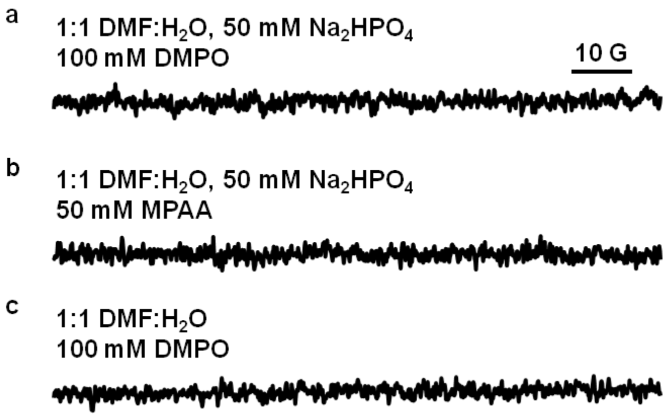

Supplementary Figure 9. Control EPR spectra for DMPO/•S-Ar adduct detection. (a) Spectrum obtained upon incubating 100 mM DMPO in 50 mM Na_2HPO_4 at pH 7.5, in a 1:1 water-DMF mixture at 25 ^{o}C. (b) Spectrum obtained upon incubating 50 mM MPAA in 50 mM Na_2HPO_4 at pH 7.5, in a 1:1 water-DMF mixture at 25 ^{o}C. (c) Spectrum obtained upon incubating 100 mM DMPO in a 1:1 water-DMF mixture at 25 ^{o}C. Spectrometer settings: microwave power, 20 mW; modulation amplitude, 1.0 G; time constant, 163 ms; scan rate, 0.6 G/s.

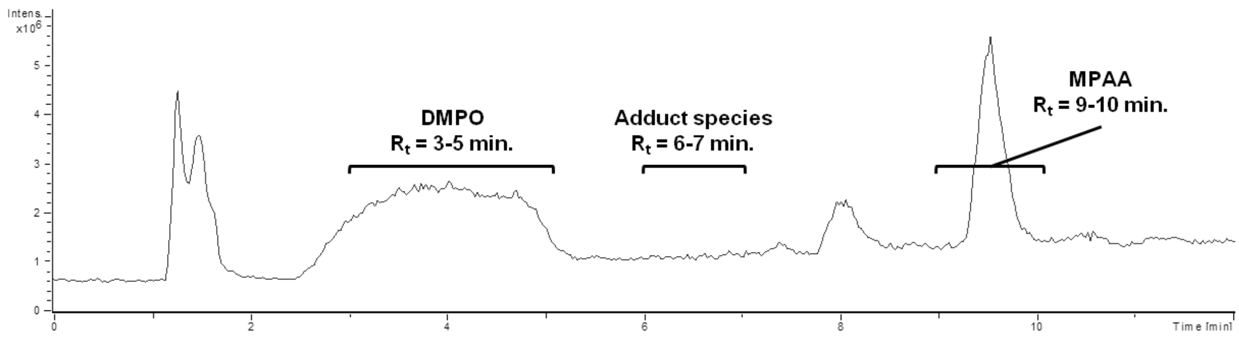

Supplementary Figure 10. Sample LC-ESI-MS trace of a DMPO reaction. Typical total ion chromatogram of a DMPO reaction containing MPAA. The sample is injected on a gradient of 30-100% D over 10 min. R_t = 3-5 min for DMPO; R_t = 6-7 min for the DMPO-OH and DMPO-MPAA adducts; R_t = 9-10 min for MPAA.

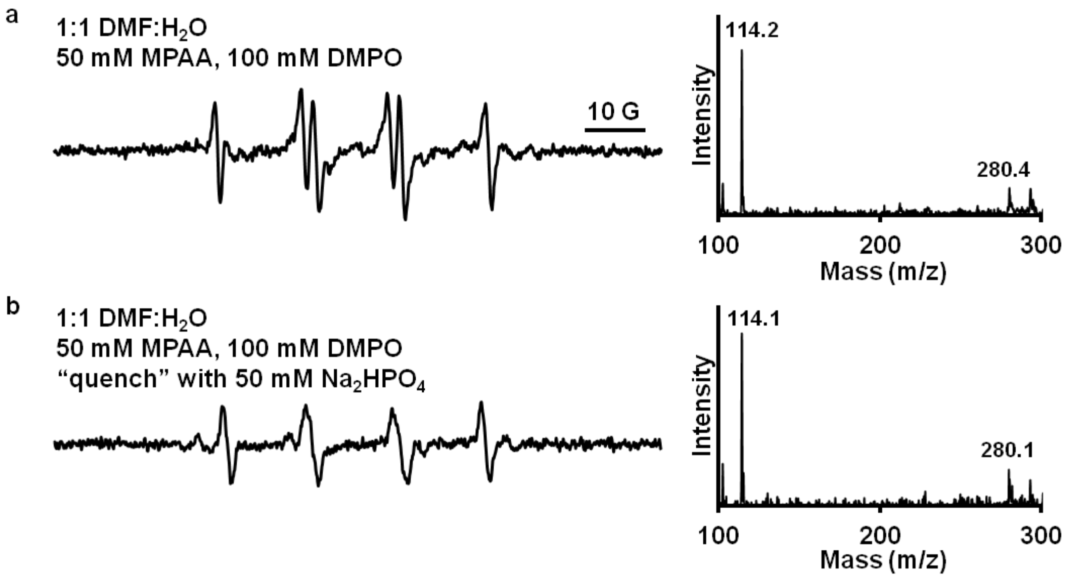

Supplementary Figure 11. EPR spectra of DMPO/•S-Ar adduct in buffered and unbuffered conditions. (a) EPR spectrum obtained upon incubating 100 mM DMPO in a 1:1 water-DMF mixture at 25 ^oC, and corresponding spectrum obtained from LC-ESI-MS analysis of the reaction mixture. (b) EPR spectrum obtained upon incubating 100 mM DMPO in a 1:1 water-DMF mixture at 25 ^oC followed by "quenching" with 600 mM Na_2HPO_4, pH 7.5, for a final concentration of 50 mM Na_2HPO_4, and corresponding spectrum obtained from LC-ESI-MS analysis of the reaction mixture. Mass at 114 m/z identified as unreacted DMPO. Spectrometer settings: microwave power, 20 mW; modulation amplitude, 1.0 G; time constant, 163 ms; scan rate, 0.6 G/s. LC-ESI-MS analysis employed a gradient of 30-100% D over 10 min. Spectra are averages of the ESI-MS signal between R_t = 6-7 min.

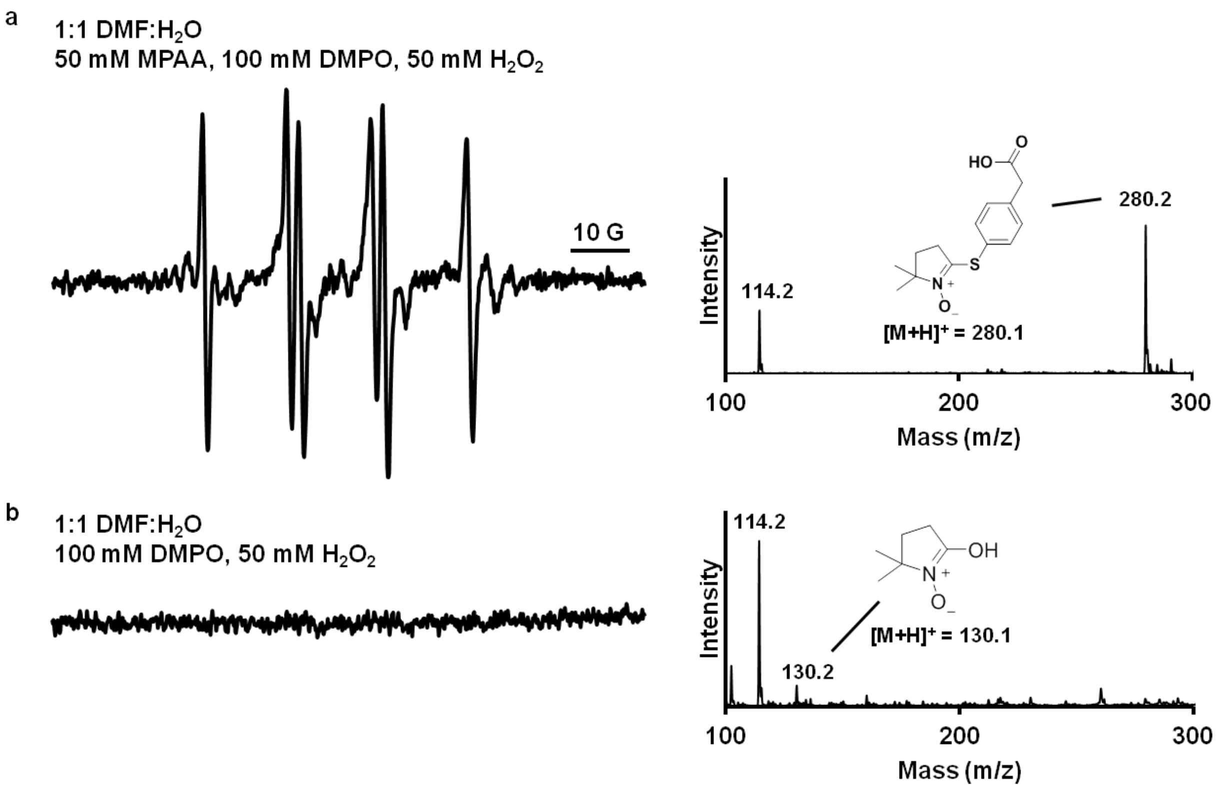

Supplementary Figure 12. EPR spectra of DMPO/•S-Ar adduct in the presence of H_2O_2. (a) EPR spectrum obtained upon incubating 50 mM MPAA, 100 mM DMPO, and 50 mM H_2O_2 in a 1:1 water-DMF mixture at 25 ^{o}C, and corresponding spectrum obtained from LC-ESI-MS analysis of the reaction mixture. (b) EPR spectrum obtained upon incubating 100 mM DMPO and 50 mM H_2O_2 in a 1:1 water-DMF mixture at 25 ^{o}C, and corresponding spectrum obtained from LC-ESI-MS analysis of the reaction mixture. Mass at 114 m/z identified as unreacted DMPO. Spectrometer settings: microwave power, 20 mW; modulation amplitude, 1.0 G; time constant, 163 ms; scan rate, 0.6 G/s. LC-ESI-MS analysis employed a gradient of 30-100% D over 10 min. Spectra are averages of the ESI-MS signal between R_t = 6-7 min.

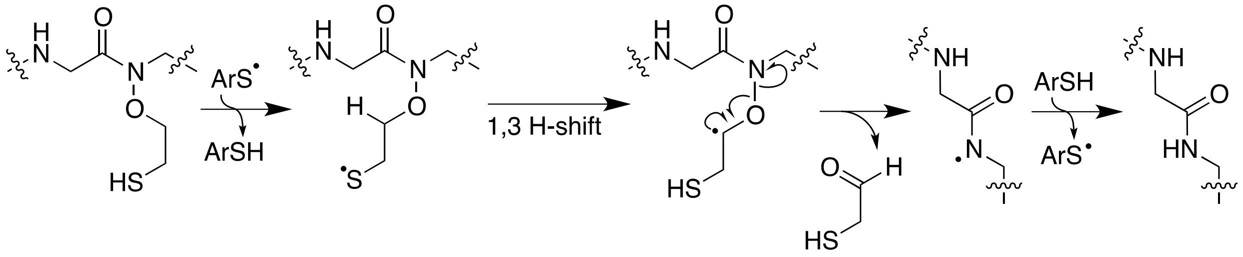

Supplementary Figure 13. Proposed mechanism of N-O bond cleavage from an auxiliary-based thiyl radical.

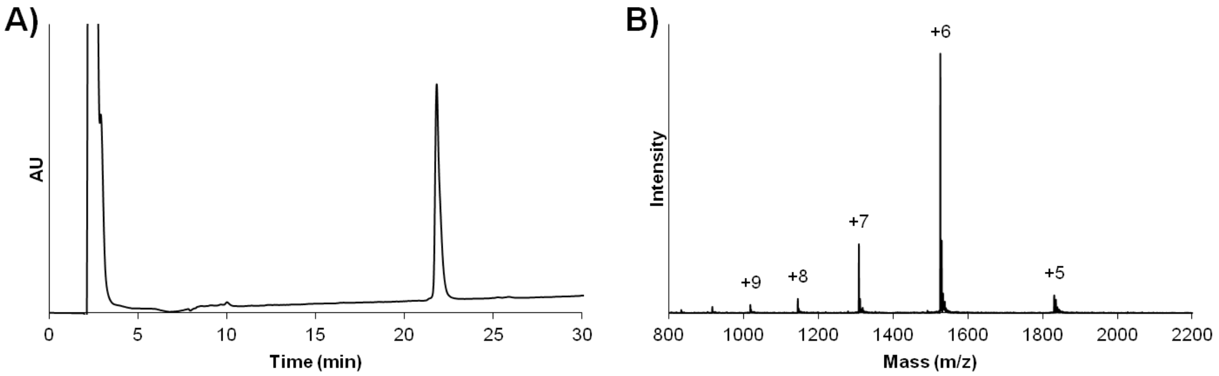

Supplementary Figure 14. Purification of *S*-alkylated KAK$^{Ub(aux)}$I. (a) C18 analytical RP-HPLC chromatogram of purified *S*-alkylated KAK$^{Ub(aux)}$I, gradient of 0-73% B, 30 min. (b) ESI-MS of purified *S*-alkylated KAK$^{Ub(aux)}$I.

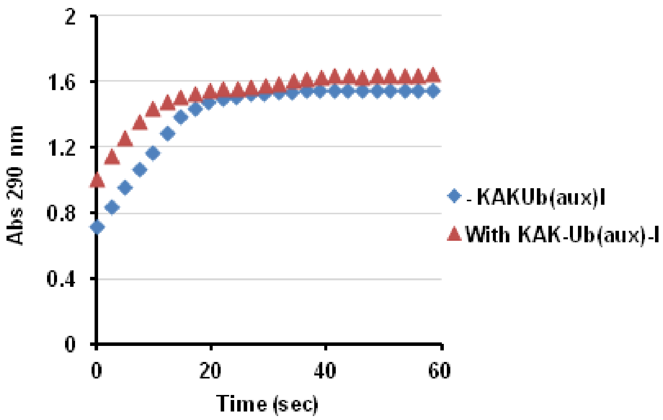

Supplementary Figure 15. Formation of uric acid from the xanthine oxidase/hypoxanthine reaction in the presence or absence of auxiliary-containing substrate. Increase in absorbance at 290 nm over time in a reaction containing 0.16 mM hypoxanthine and 0.14 u/mL xanthine oxidase, with (red triangle) or without (blue diamond) 0.04 mM KAK$^{Ub(aux)}$I.

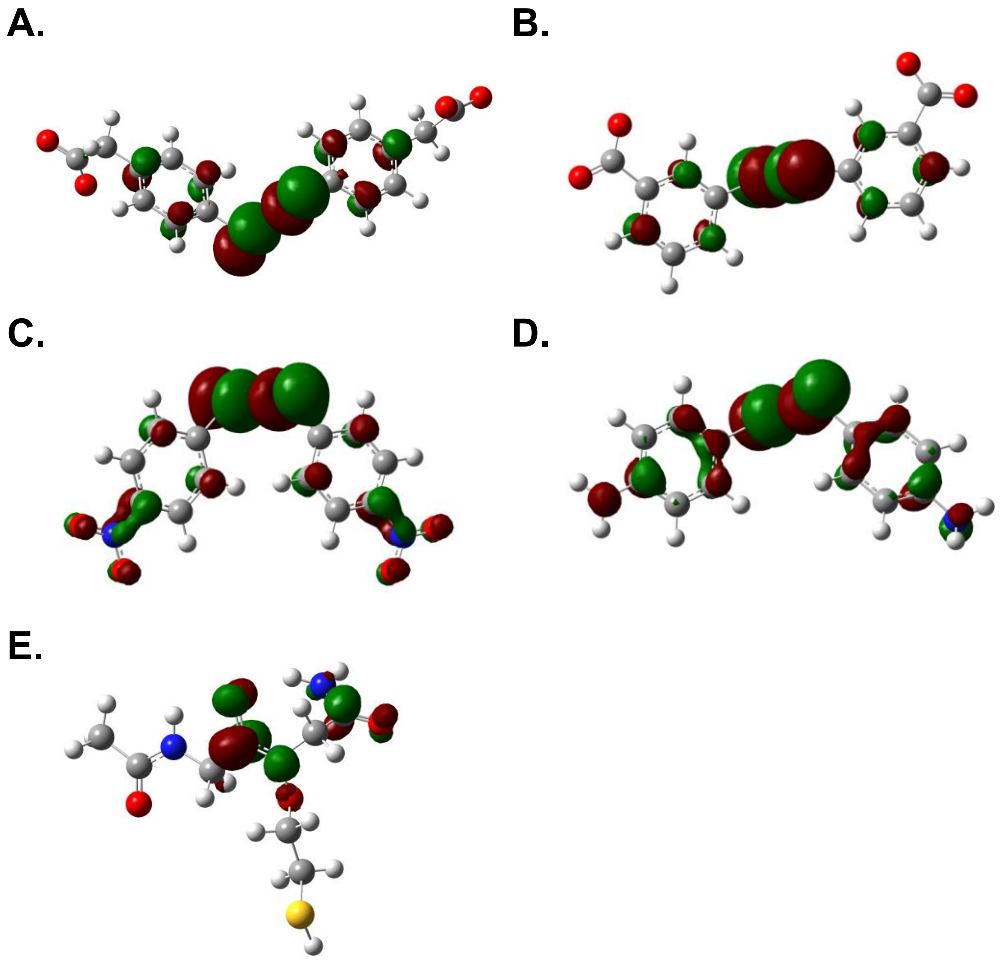

Supplementary Figure 16. Calculated HOMOs of reducing agents and LUMO of the model peptide substrate. (a) HOMO of MPAA disulfide radical anion; (b) HOMO of 3-Mercaptobenzoic acid disulfide radical anion; (c) HOMO of *p*-Nitrothiophenol disulfide radical anion; (d) HOMO of p-Aminothiophenol radical anion; (e) LUMO of model diglycine peptide **2**.

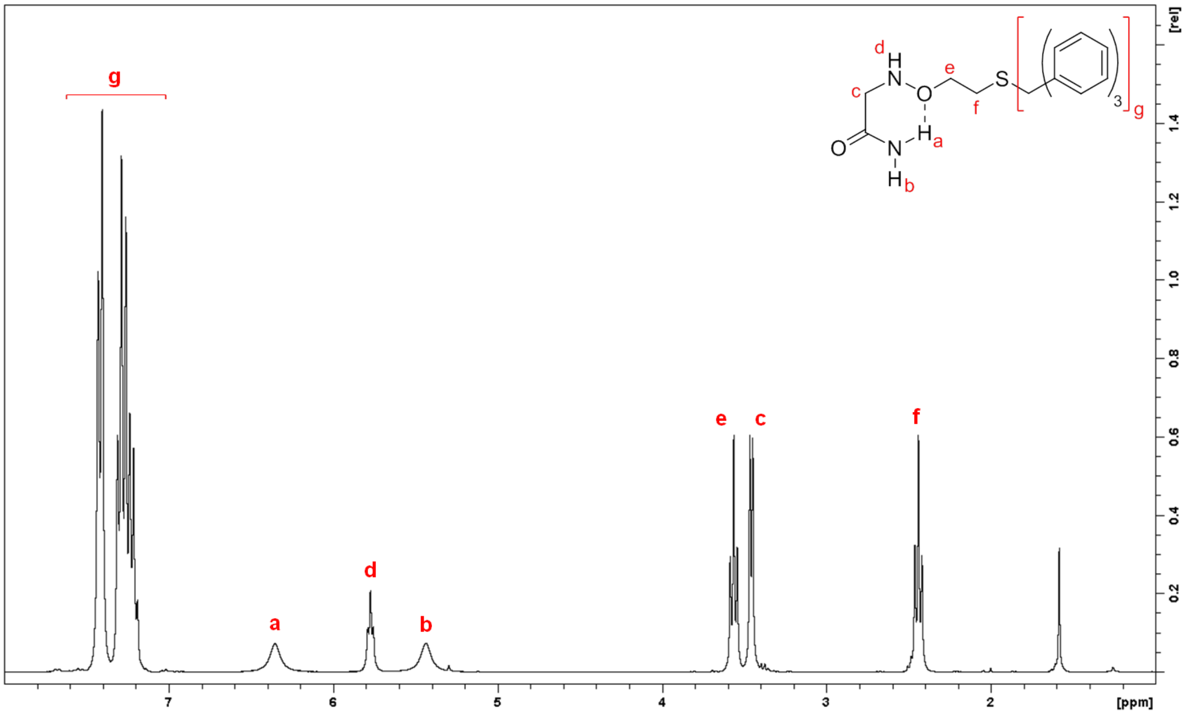

Supplementary Figure 17a. ^{1}H-NMR of 2-((2-(tritylthio)ethoxy)amino)acetamide. Peak at 1.58 ppm (s) identified as H_2O.

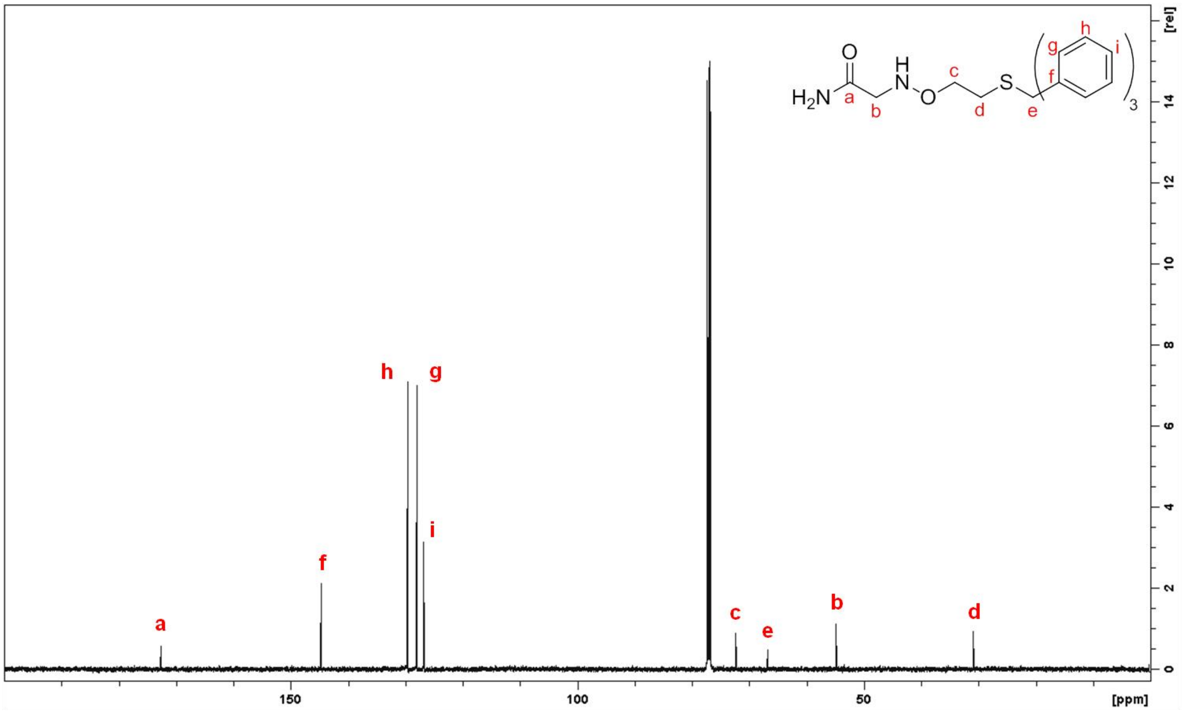

Supplementary Figure 17b. ^{13}C-NMR of 2-((2-(tritylthio)ethoxy)amino)acetamide. $CDCl_3$ solvent peak identified at 77.05 ppm (t).

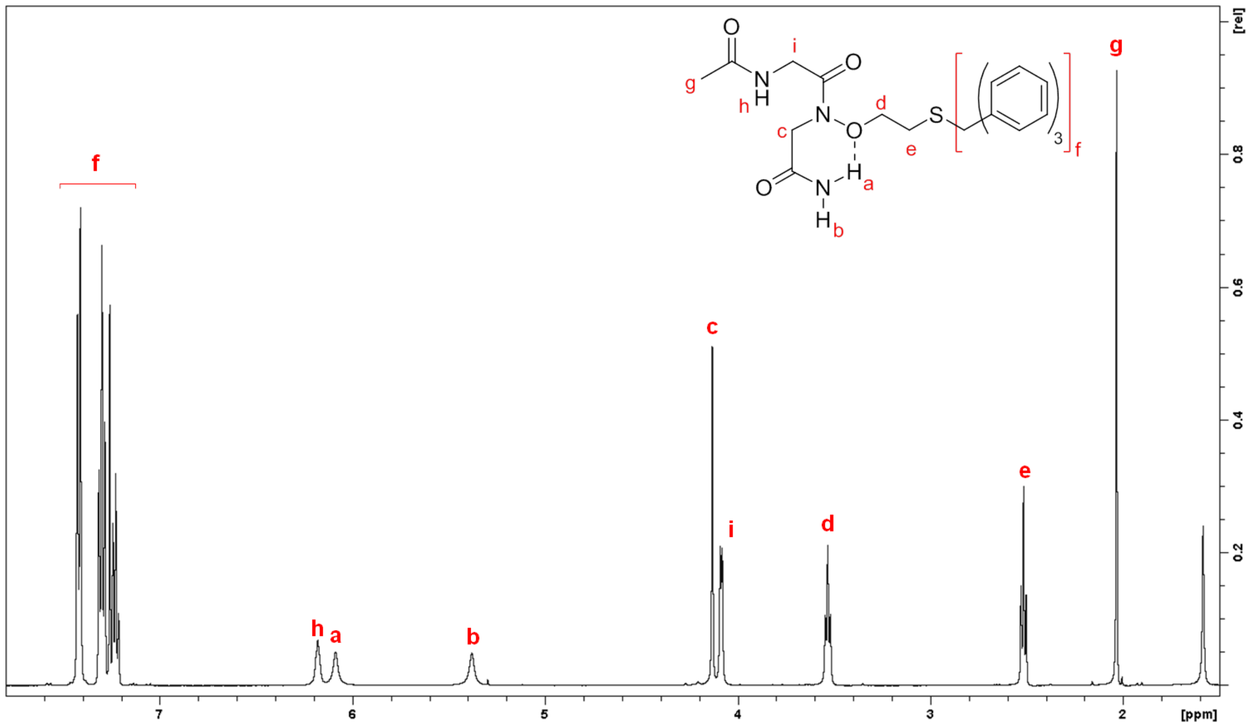

Supplementary Figure 18a. ^{1}H-NMR of model dipeptide 2, 2-acetamido-*N*-(2-amino-2-oxoethyl)-*N*-(2-(tritylthio)ethoxy)acetamide. Peak at 1.58 ppm (s) identified as H_2O.

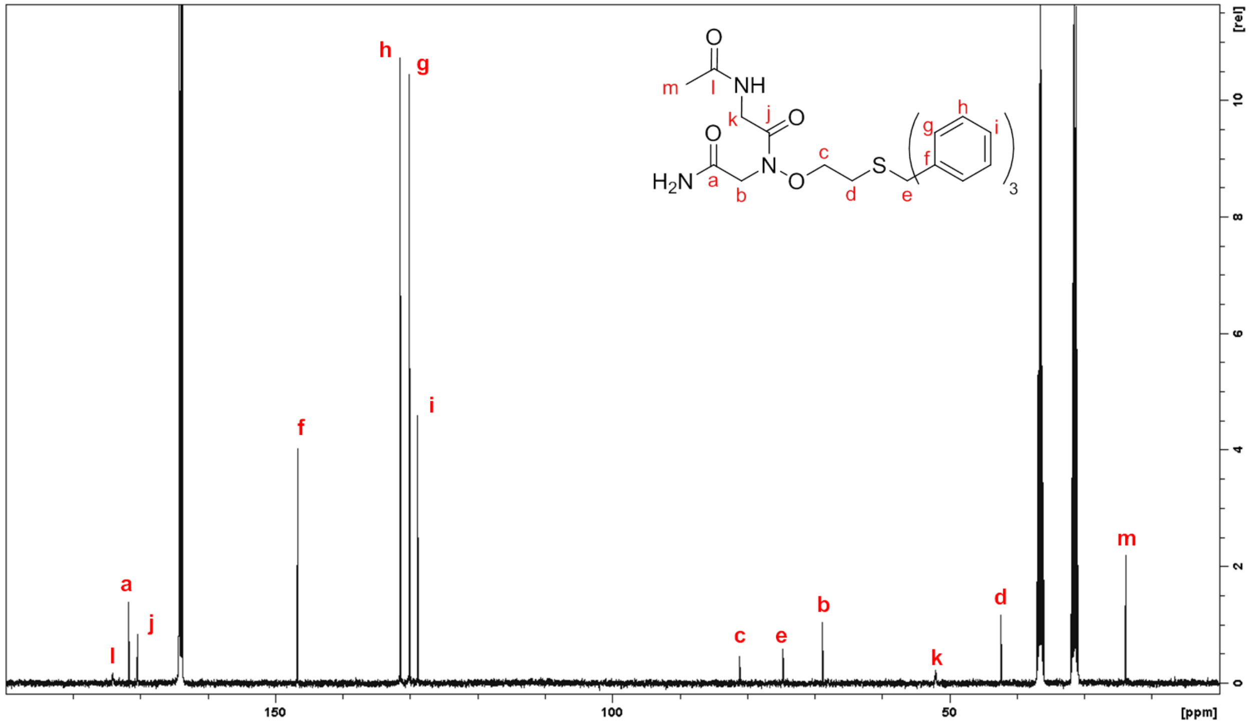

Supplementary Figure 18b. ^{13}C-NMR of model dipeptide 2, 2-acetamido-*N*-(2-amino-2-oxoethyl)-*N*-(2-(tritylthio)ethoxy)acetamide. DMF-D_7 solvent peaks observed at 164.04 (t), 36.55 (m), and 31.44 (m) ppm.

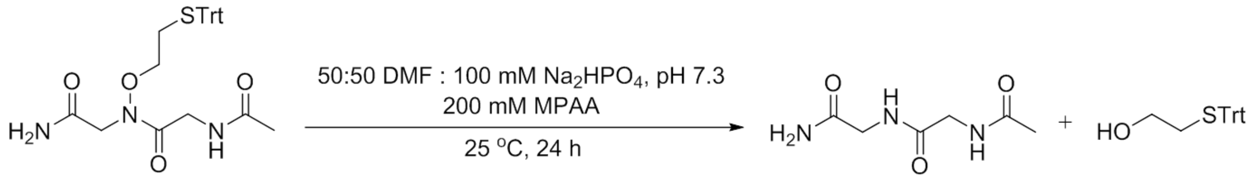

Supplementary Figure 19a. Formation of 2-(tritylthio)ethanol from N-O bond reduction in model dipeptide 2.

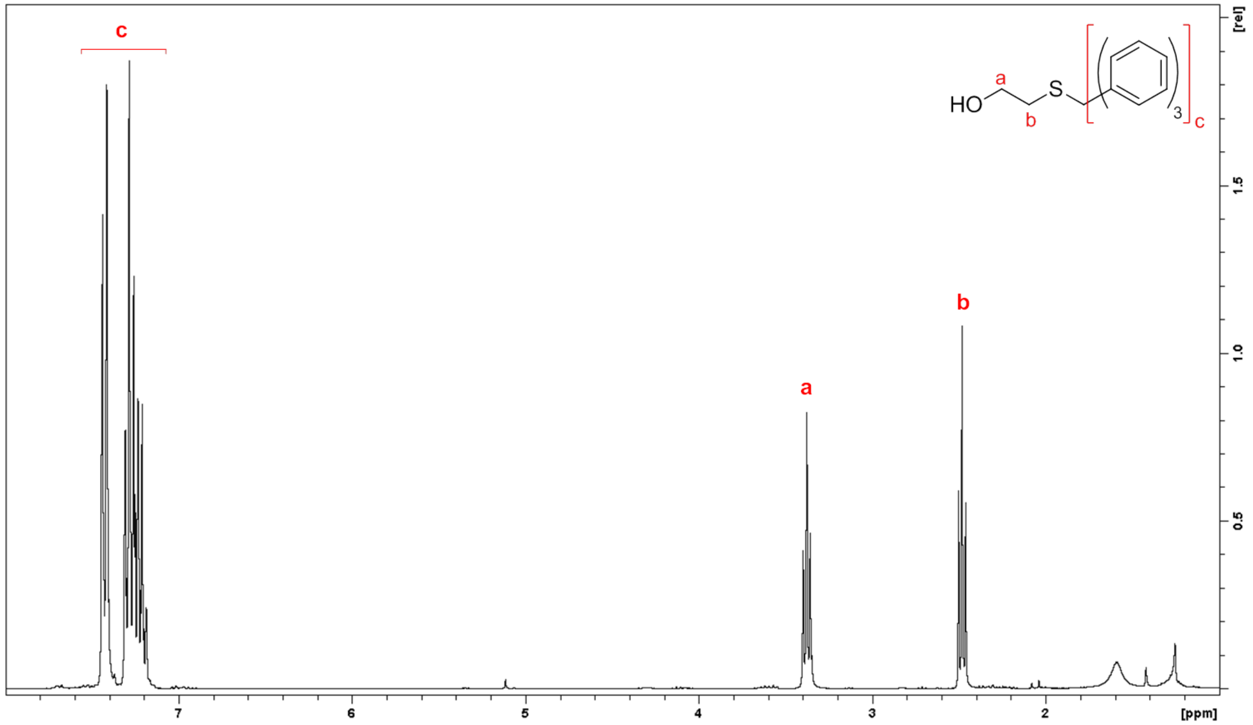

Supplementary Figure 19b. ^{1}H-NMR of 2-(tritylthio)ethanol. Peak at 1.59 ppm (s) identified as H_2O.

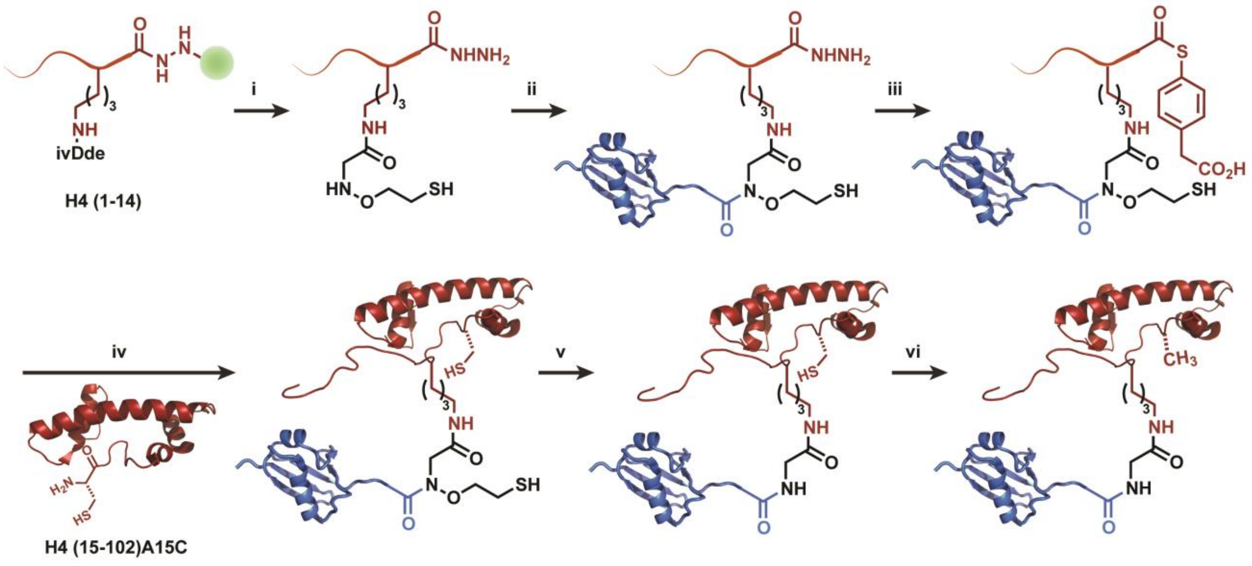

Supplementary Figure 20. Semisynthesis of full-length sumoylated histone H4. i, Site-specific coupling of the ligation auxiliary to H4(1-14) Lys12 followed by acidolytic release of the fully unprotected peptidyl hydrazide from the solid-phase. **ii,** Expressed protein ligation of H4(1-14)^{aux}-C(O)NHNH$_2$ with SUMO-3(2-91)C47S-α-thioester to generate sumoylated peptide hydrazide, H4(1-14)$^{Su(C47S)(aux)}$-C(O)NHNH$_2$. **iii,** Conversion of H4(1-14)$^{Su(C47S)(aux)}$-C(O)NHNH$_2$ to the C-terminal MPAA α-thioester. **iv,** Native chemical ligation of the H4(1-14)$^{Su(C47S)(aux)}$-C(O)SR MPAA thioester with the H4(15-102)A15C truncant protein to yield full-length sumoylated H4(A15C), with retention of the ligation auxiliary. **v,** Selective removal of the ligation auxiliary with 200 mM MPAA under non-denaturing conditions to yield sumoylated H4(A15C). **vi,** Radical mediated desulfurization of Cys15 in H4 to generate full-length wild-type sumoylated histone H4. ivDde= 1-(4,4-Dimethyl-2,6-dioxocyclohexylidene)-3-methylbutyl group. PDB code for SUMO-3, 1U4A. PDB code for H4, 1KX5.

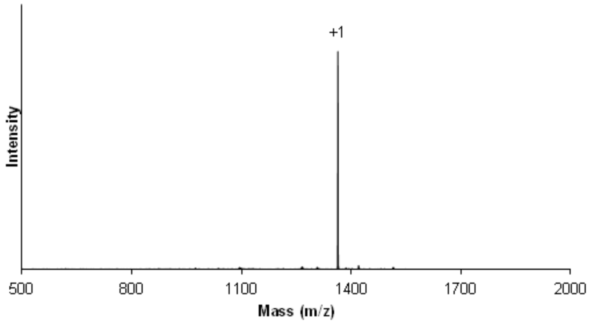

Supplementary Figure 21. ESI-MS of purified H4(1-14)^{aux}-C(O)NHNH$_2$.

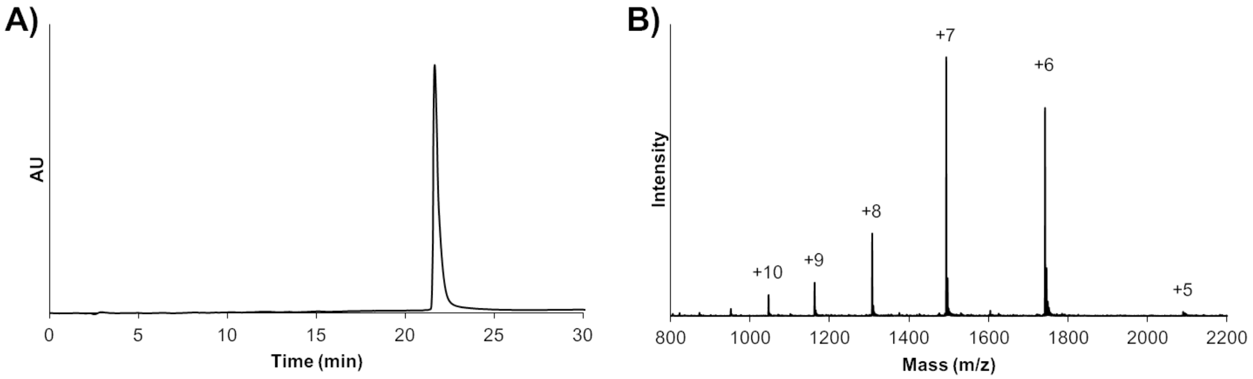

Supplementary Figure 22. Purification of SUMO-3(2-91)C47S-MES. (a) C18 analytical RP-HPLC chromatogram of purified SUMO-3(2-91)C47S-MES, gradient of 0-73% B, 30 min. (b) ESI-MS of purified SUMO-3(2-91)C47S-MES.

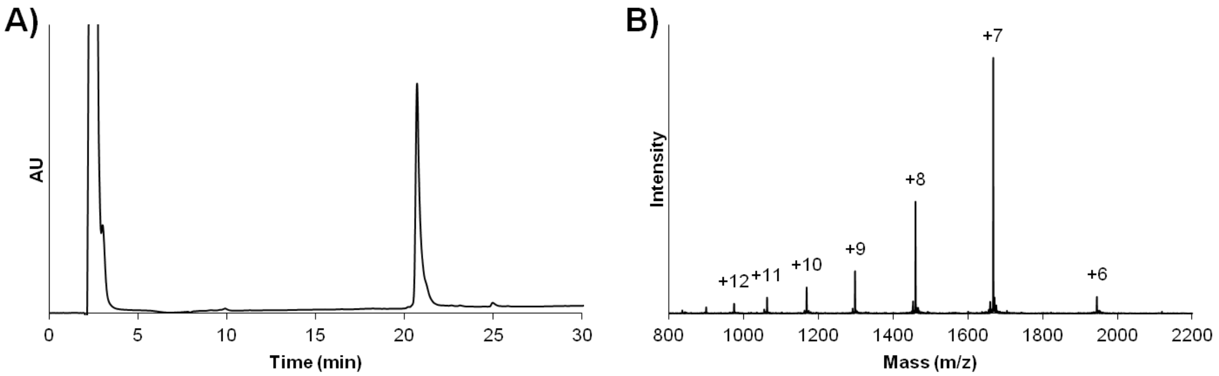

Supplementary Figure 23. Purification of H4(1-14)$^{Su(C47S)(aux)}$-C(O)NHNH$_2$. (a) C18 analytical RP-HPLC chromatogram of purified H4(1-14)$^{Su(C47S)(aux)}$-C(O)NHNH$_2$, gradient of 0-73% B, 30 min. (b) ESI-MS of purified H4(1-14)$^{Su(C47S)(aux)}$-C(O)NHNH$_2$.

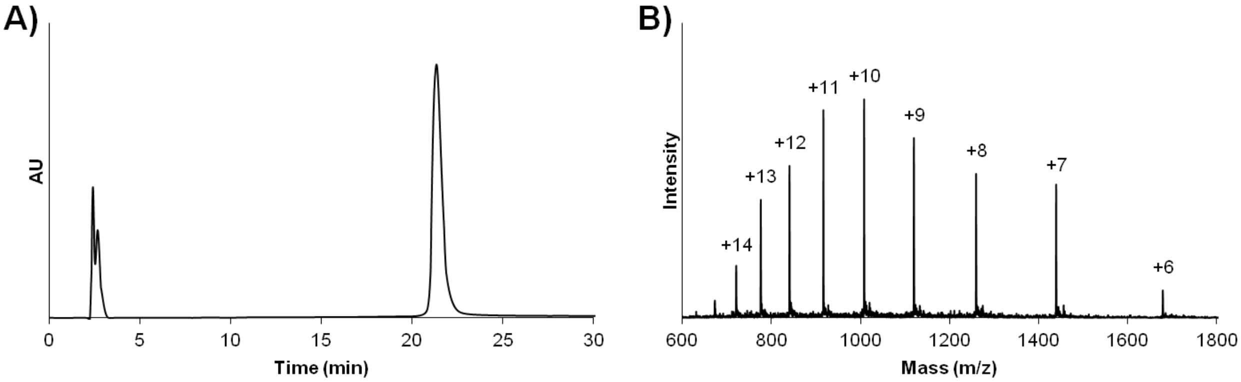

Supplementary Figure 24. Purification of H4(15-102)A15C. (a) C4 analytical RP-HPLC chromatogram of purified H4(15-102)A15C, gradient of 0-100% B, 30 min. (b) ESI-MS of purified H4(15-102)A15C.

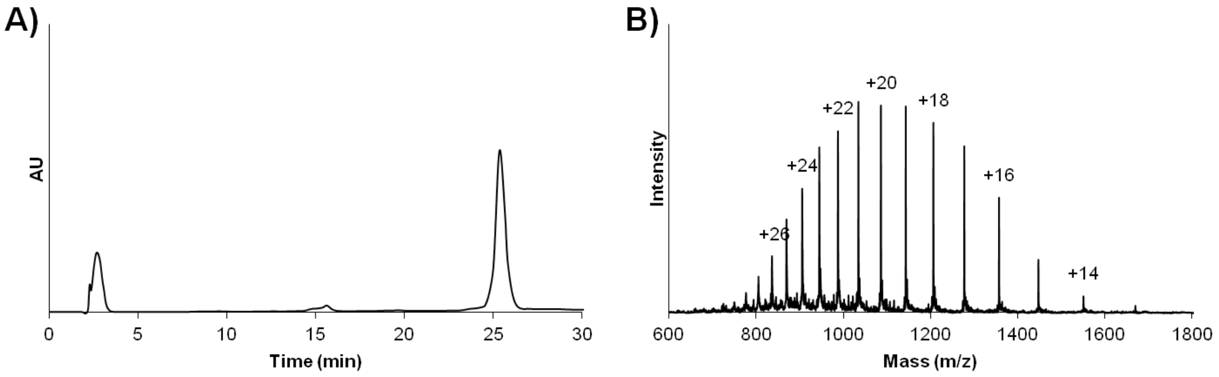

Supplementary Figure 25. Purification of H4(A15C)$^{\mathbf{Su(C47S)(aux)}}$. (a) C4 analytical RP-HPLC chromatogram of purified H4(A15C)$^{Su(C47S)(aux)}$, gradient of 0-73% B, 30 min. (b) ESI-MS of purified H4(A15C)$^{Su(C47S)(aux)}$.

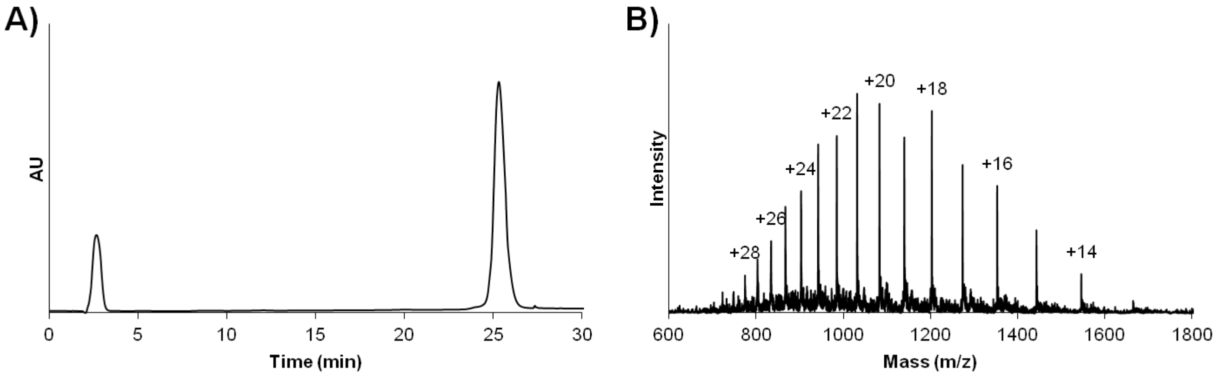

Supplementary Figure 26. Purification of H4(A15C)$^{\mathbf{Su(C47S)}}$. (a) C4 analytical RP-HPLC chromatogram of purified H4(A15C)$^{Su(C47S)}$, gradient of 0-73% B, 30 min. (b) ESI-MS of purified H4(A15C)$^{Su(C47S)}$.

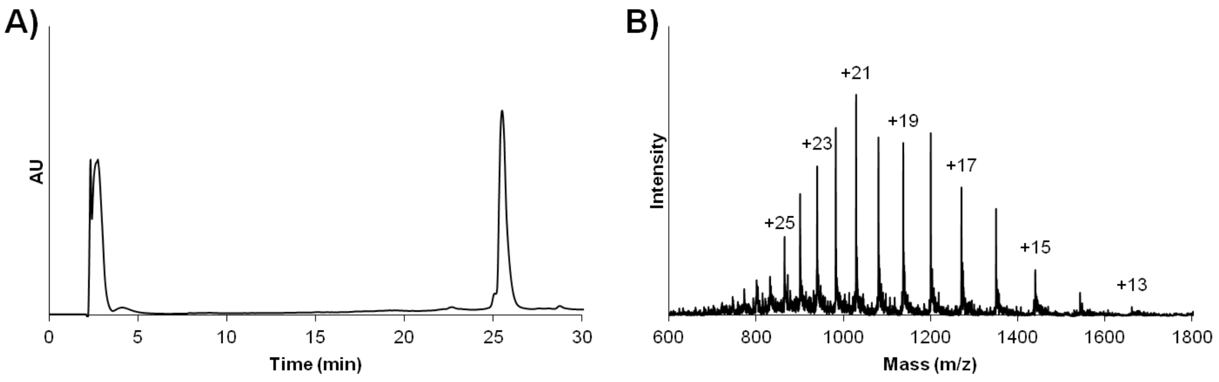

Supplementary Figure 27. Purification of suH4. (a) C4 analytical RP-HPLC chromatogram of purified suH4, gradient of 0-73% B, 30 min. (b) ESI-MS of purified suH4.

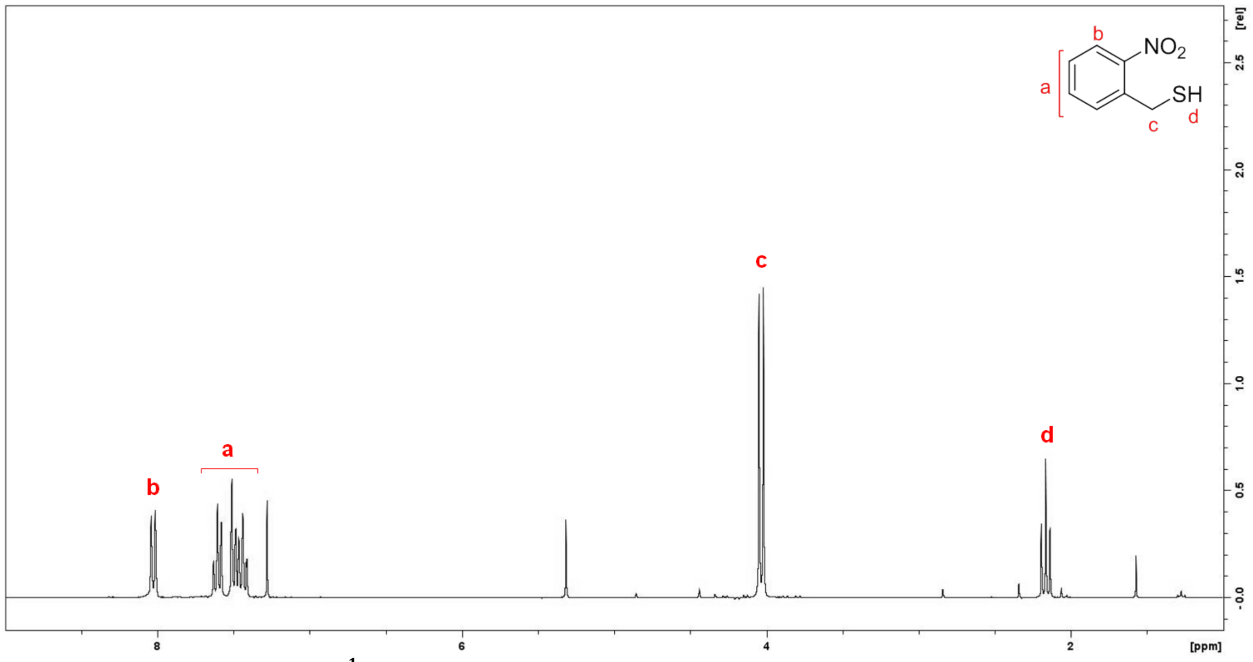

Supplementary Figure 28a. ^{1}H-NMR of (2-nitrobenzyl)thiol.

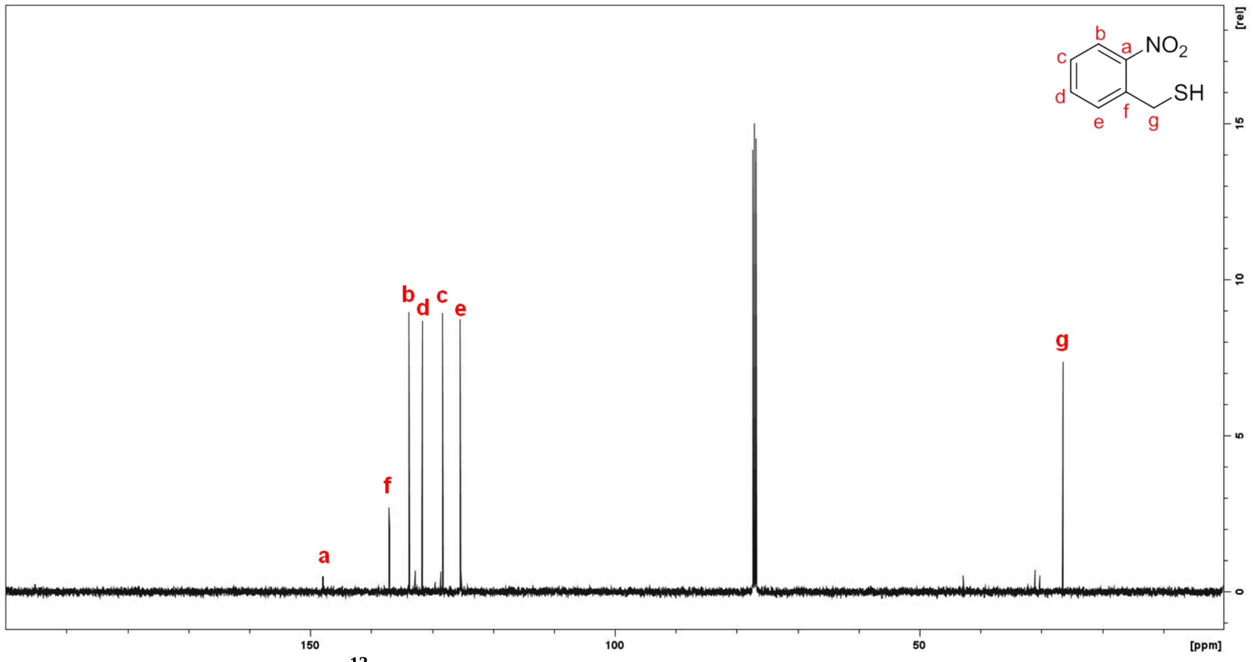

Supplementary Figure 28b. ^{13}C-NMR (2-nitrobenzyl)thiol.

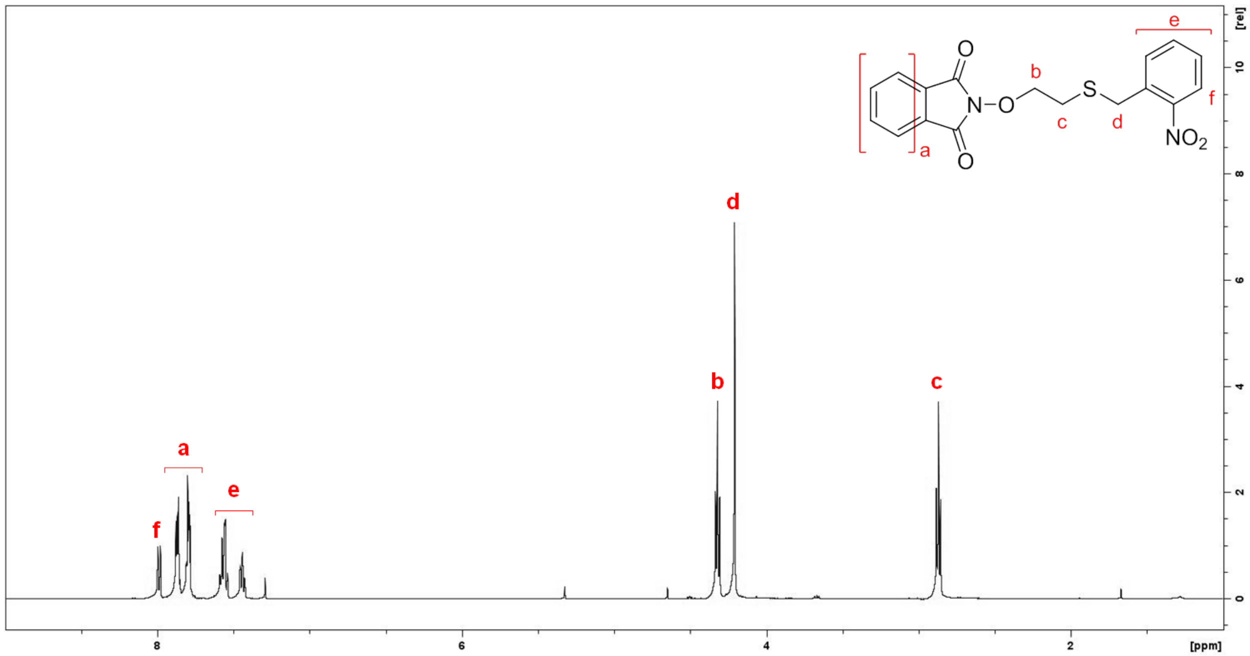

Supplementary Figure 29a. **^{1}H-NMR of *N*-(2-((2-nitrobenzyl)thio)ethoxy)phthalimide.**

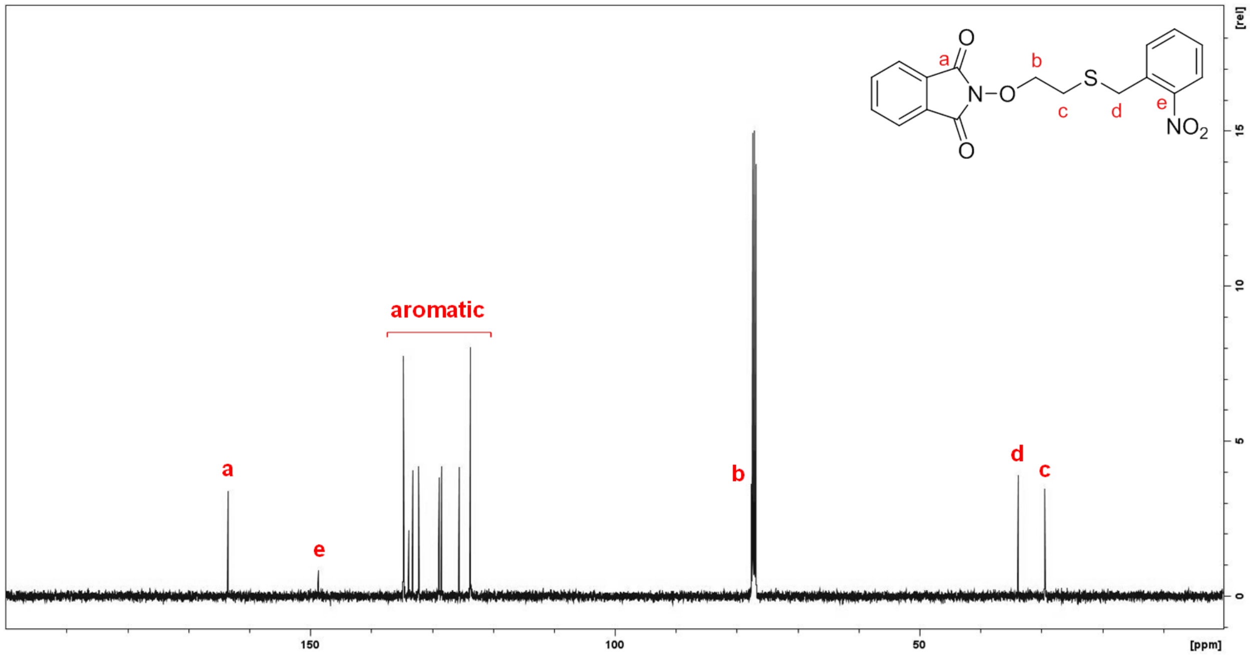

Supplementary Figure 29b. **^{13}C-NMR of *N*-(2-((2-nitrobenzyl)thio)ethoxy)phthalimide.**

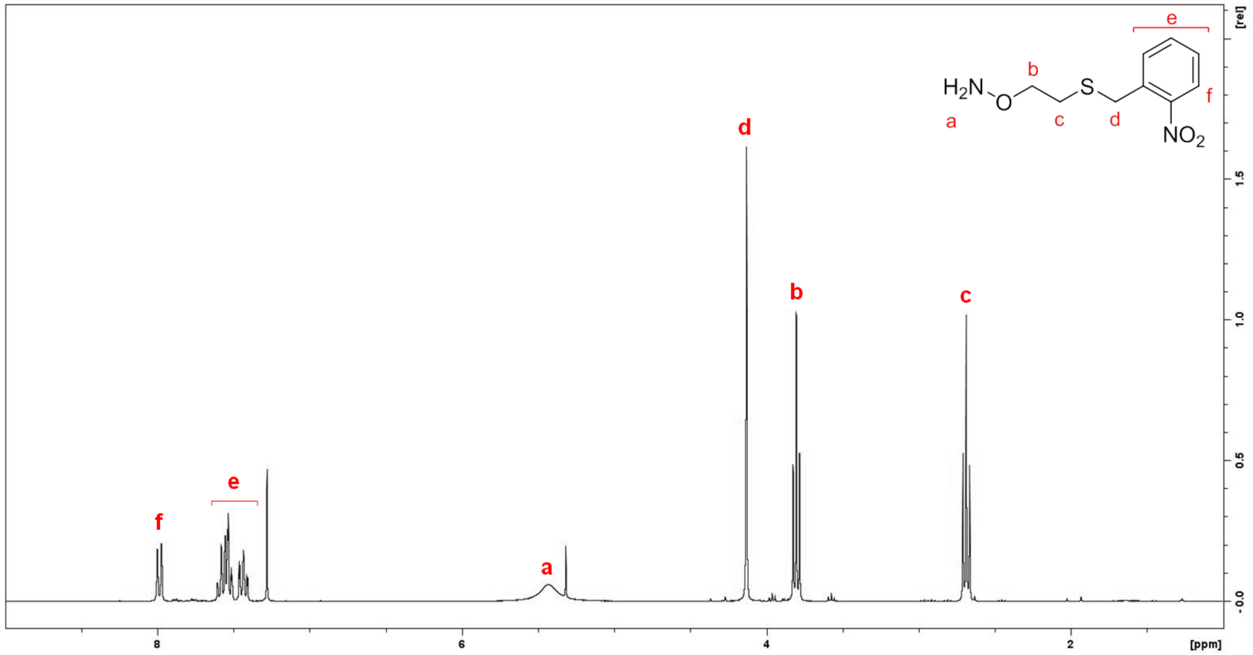

Supplementary Figure 30a. ^{1}**H-NMR of *O*-(2-((2-nitrobenzyl)thio)ethyl)hydroxylamine (3).**

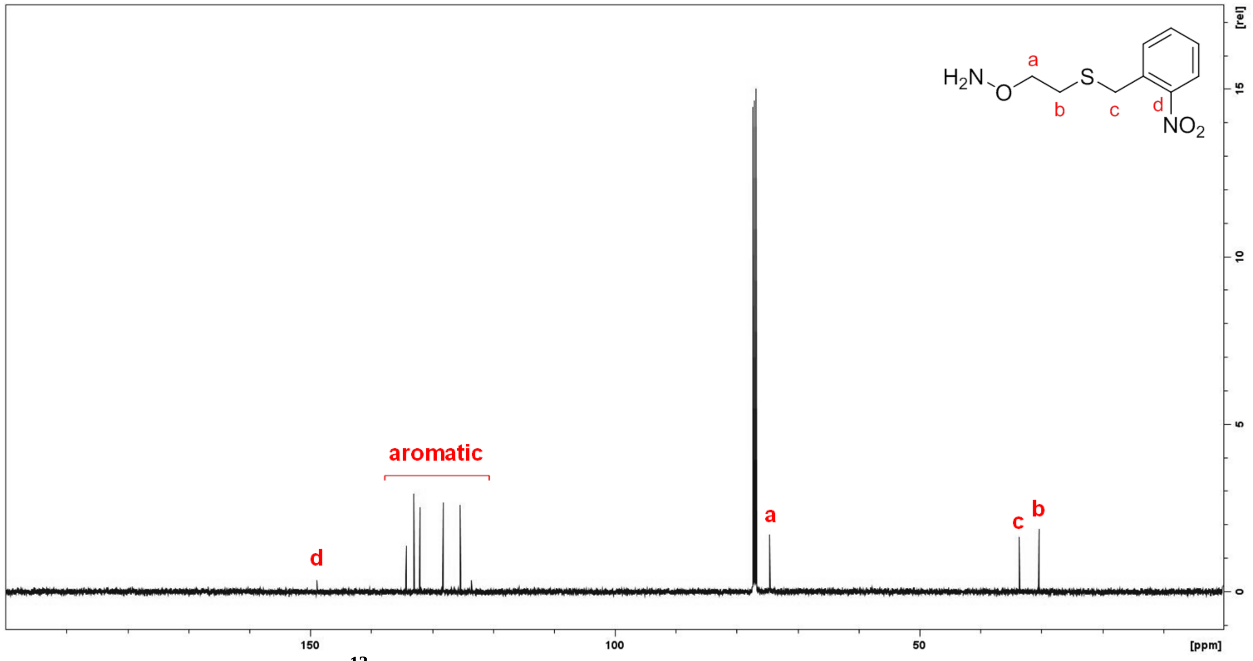

Supplementary Figure 30b. ^{13}**C-NMR of *O*-(2-((2-nitrobenzyl)thio)ethyl)hydroxylamine (3).**

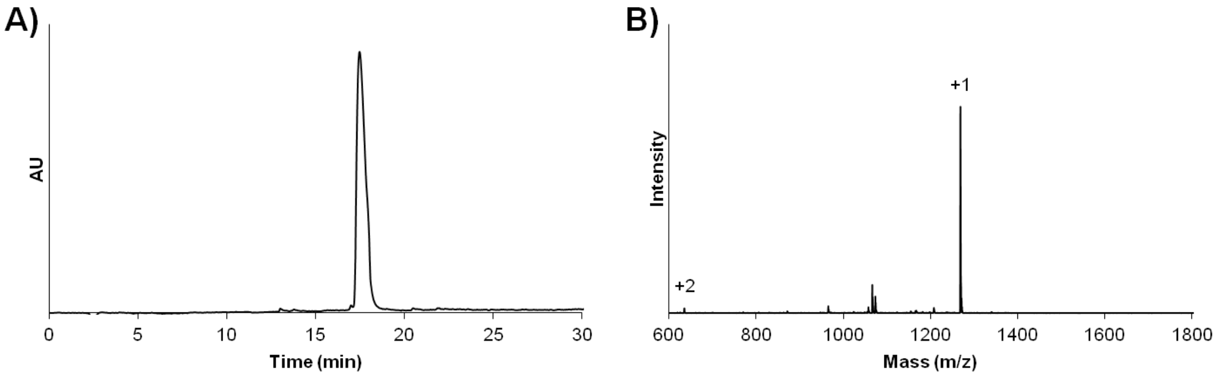

Supplementary Figure 31. Purification of H2B(117-125, A117C)^{photoaux} (4). (a) C18 analytical RP-HPLC chromatogram of purified H2B(117-125, A117C)^{photoaux}, gradient of 0-50% B, 30 min. (b) ESI-MS of purified H2B(117-125, A117C)^{photoaux}.

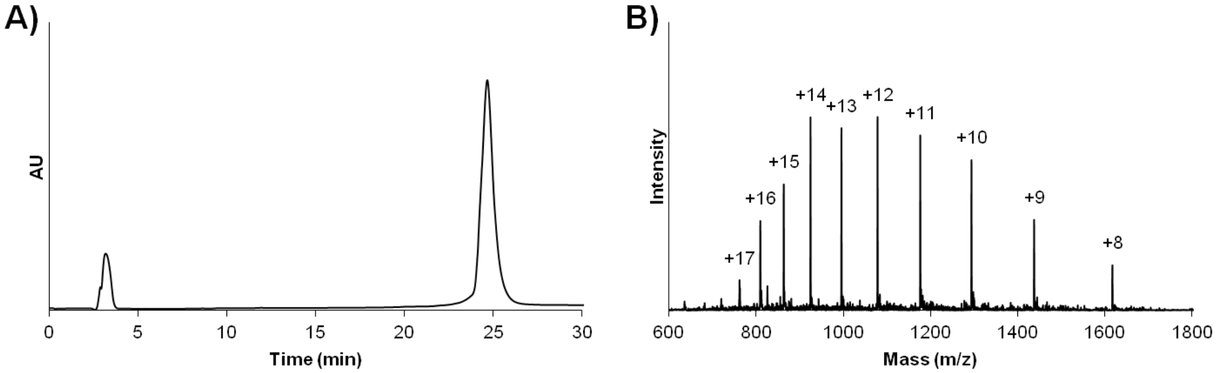

Supplementary Figure 32. Purification of H2B(1-116)-MES. (a) C4 analytical RP-HPLC chromatogram of purified of H2B(1-116)-MES, gradient of 0-73% B, 30 min. (b) ESI-MS of purified of H2B(1-116)-MES.

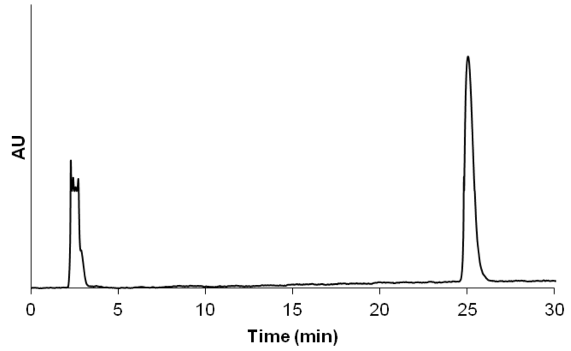

Supplementary Figure 33. Purification of H2B(A117C)^{photoaux} (5). C4 analytical RP-HPLC chromatogram of purified of H2B(A117C)^{photoaux}, gradient of 0-73% B, 30 min.

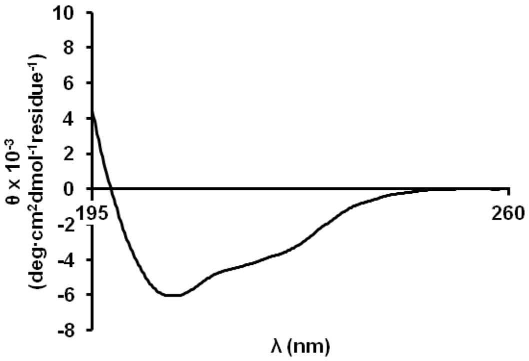

Supplementary Figure 34. Refolded SUMO-3(2-91)C47S-MES. Circular Dichroism spectrum of SUMO-3(2-91)C47S-MES at 0.3 mg/mL in 50 mM Na_2HPO_4, pH 7.5.

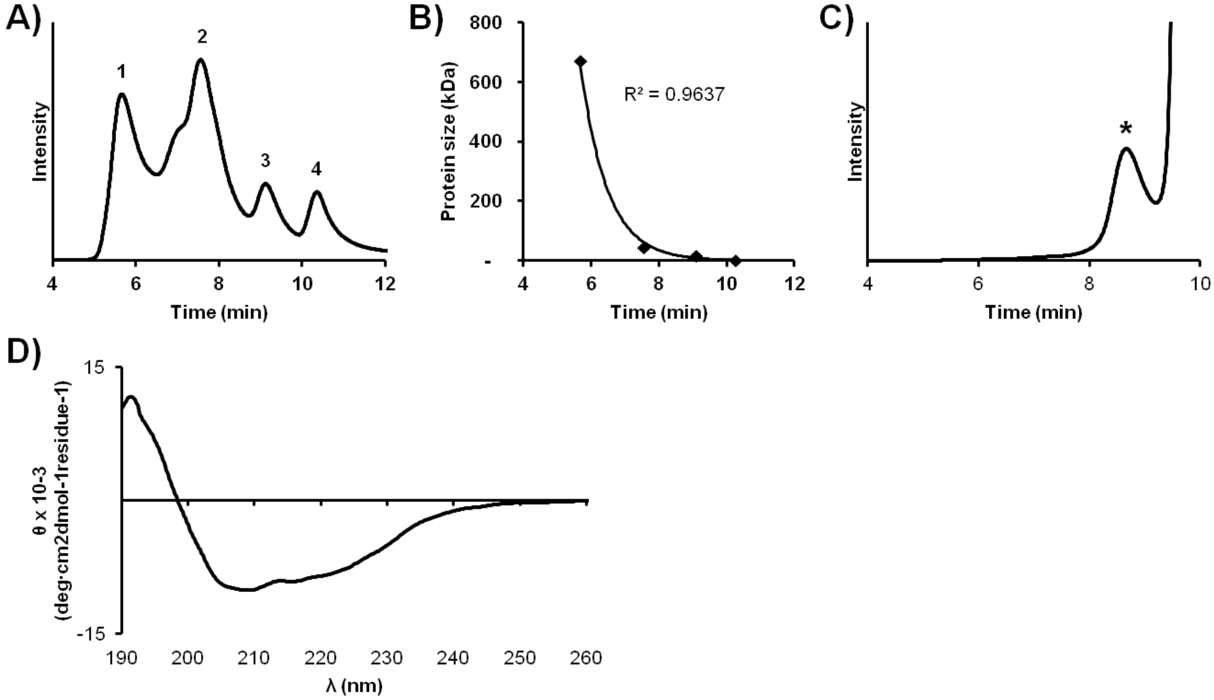

Supplementary Figure 35. Size exclusion chromatography and circular dichroism of H2B(A117C)$^{Su(C47S)}$ (8). (a) Size exclusion UV chromatogram of protein standards in 50 mM Na_2HPO_4, pH 7.5, at 4 °C, run at 1 mL/min. Protein standards: 1) 670,000 Da, R_t = 5.69 min; 2) 44,000 Da, R_t = 7.56 min; 3) 17,000 Da, R_t = 9.11 min; 4) 1,350 Da, R_t = 10.28 min. (b) Protein standard data fit to an exponential equation. (c) Size exclusion UV chromatogram of the crude MPAA-mediated N-O bond cleavage auxiliary removal reaction. Calculated mass of the asterisked peak (R_t = 8.55 min) is 18,000 Da. Large signal beginning at R_t = 9.5 min. attributed to MPAA. (d) Circular Dichroism spectrum of the ~20 kDa SEC peak in 50 mM Na_2HPO_4, pH 7.5.

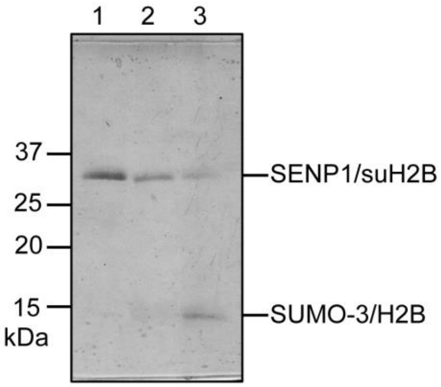

Supplementary Figure 36. SENP1 hydrolysis of H2B(A117C)$^{Su(C47S)}$ (8). Coomassie-stained 18% SDS-PAGE gel, run at 200 V for 1.5 h showing products of the SENP1 hydrolysis assay. Lane 1= SENP1 alone, 2= H2B(A117C)$^{Su(C47S)}$ (**8**) alone, 3= SENP1 and **8** incubated for 24 h.

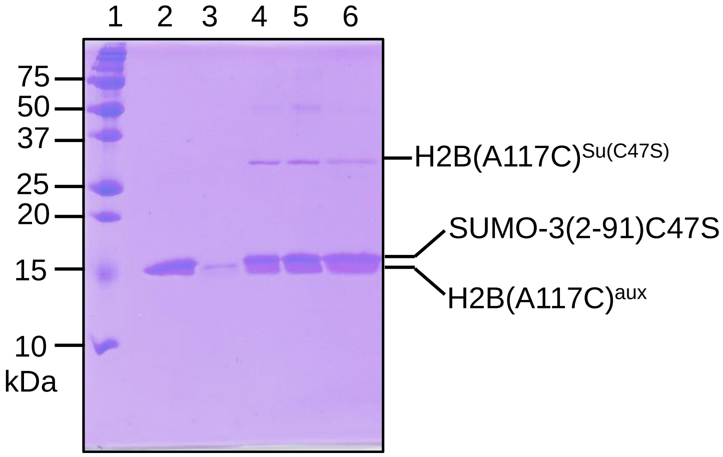

Supplementary Figure 37. Sumoylation of folded histone H2B. Entire coomassie-stained 15% SDS-PAGE gel of ligation between H2B(A117C)^{aux} (**6**) and SUMO3(2-91)C47S-α-thioester under non-denaturing conditions. Lane 1: Bio-Rad Protein Molecular Weight Ladder. Lane 2: SUMO3(2-91)C47S-MES. Lane 3: H2B(A117C)^{aux}. Lane 4: 24 h ligation reaction. Lane 5: 48 h ligation reaction. Lane 6: 24 h MPAA-mediated N-O bond cleavage auxiliary removal reaction.

Supplementary Table 1. Requirements for auxiliary removal under non-denaturing conditions.[a]

		Reaction Conditions	Yield
Buffer	**1**	200 mM MPAA, 10 mM TCEP, 50 mM tris, 150 mM NaCl, pH 7.5	61%
	**2**	200 mM MPAA, 10 mM TCEP, 100 mM Na_2HPO_4, pH 7.5	58%
Additives	**3**	10 mM TCEP, 50 mM tris, 150 mM NaCl, pH 7.5	n.d.
	**4**	200 mM MPAA, 50 mM tris, 150 mM NaCl, pH 7.5	63%
[MPAA]	**5**	100 mM MPAA, 100 mM Na_2HPO_4, pH 7.3	56%
	**6**	50 mM MPAA, 100 mM Na_2HPO_4, pH 7.3	55%
	**7**	5 mM MPAA, 100 mM Na_2HPO_4, pH 7.3	2%
Contaminant	**8**	200 mM HPLC-purified[b] MPAA, 100 mM Na_2HPO_4, pH 7.3	72%
Oxidized MPAA	**9**	100 mM MPAA disulfide, 100 mM Na_2HPO_4, pH 7.3	n.d.
pH	**10**	200 mM MPAA, 100 mM Na_2HPO_4, pH 6.0	2%
	**11**	200 mM MPAA, 100 mM Na_2HPO_4, pH 7.3	73%
	**12**	200 mM MPAA, 100 mM Na_2HPO_4, pH 8.0	58%
Oxygen	**13**	200 mM MPAA, 100 mM Na_2HPO_4, pH 7.3, degassed[c]	2%
	**14**	200 mM MPAA, 100 mM Na_2HPO_4, pH 8.5, degassed[c]	5%

[a]Reaction conditions were evaluated for auxiliary removal from the ligation product $KAK^{Ub(aux)}I$. $KAK^{Ub(aux)}I$ (30 nmol, ~0.3 mg) was dissolved in 300 μL buffer and incubated in a 1.5 mL microcentrifuge tube for 48 h at 25 °C. Following incubation the reactions were treated with 100 mM TCEP at 4 °C for 30 min., then acidified to pH ~3 with formic acid, extracted once with diethyl ether, and analyzed by LC-ESI-MS employing a gradient of 0-100% D over 40 min. Percent yield was determined by the percent auxiliary-free ligation product $KAK^{Ub}I$ relative to other species present, determined by ESI-MS signal intensity for R_t = 14-17 min. [b]MPAA was dissolved in 30% buffer B and purified by C18 preparative HPLC employing a gradient of 30-80% B over 60 min. [c]Buffer was subjected to three cycles of freeze-thaw degassing under Argon and kept under Argon atmosphere. n.d.= no detectable N-O bond cleavage.

Supplementary Table 2. Conditions for auxiliary removal in the presence of hydrogen peroxide[a] or superoxide.

	Reaction Conditions	Time (h)	Yield
**1**	100 mM Na_2HPO_4, pH 7.5, 0.32 mM hypoxanthine, xanthine oxidase[b]	24	n.d.
**2**	200 mM MPAA, 50 mM H_2O_2, 100 mM Na_2HPO_4, pH 7.3	4	79%
**3**	200 mM MPAA, 50 mM EDTA, 100 mM Na_2HPO_4, pH 7.3	24	4%
**4**	200 mM MPAA, 1 mM $FeCl_2$, 100 mM Na_2HPO_4, pH 7.3	24	87%
**5**	50 mM H_2O_2, 100 mM Na_2HPO_4, pH 7.3	24	n.d.
**6**	50 mM H_2O_2, 1 mM $FeCl_2$, 100 mM Na_2HPO_4, pH 7.3	24	n.d.
**7**	200 mM MPAA, 50 mM H_2O_2, 100 mM Na_2HPO_4, pH 7.3, degassed[c]	4	68%

[a]Reaction conditions were evaluated for auxiliary removal from the ligation product $KAK^{Ub(aux)}I$. $KAK^{Ub(aux)}I$ (10 nmol, ~0.1 mg) was dissolved in 100 µL of buffer. To this was added 0.51 µL of a 30% (9.8 M) solution of H_2O_2 for a final concentration of 50 mM H_2O_2. The reaction was incubated in a 1.5 mL microcentrifuge tube at 25 °C. Following incubation the reactions were treated with 100 mM TCEP at 4 °C for 30 min., then acidified to pH ~3 with formic acid, extracted once with diethyl ether, and analyzed by LC-ESI-MS employing a gradient of 0-100% D over 40 min. Percent yield was determined by the percent auxiliary-free ligation product $KAK^{Ub}I$ relative to other species present, determined by ESI-MS signal intensity for R_t = 14-17 min. [b]$KAK^{Ub(aux)}I$ was dissolved at 0.04 mM in 100 µL of 50 mM Na_2HPO_4, pH 7.5 in the presence of 0.32 mM hypoxanthine and 0.14 u/mL xanthine oxidase at 25 °C for 24 h. [c]Buffer was subjected to three cycles of freeze-thaw degassing under Argon and kept under Argon atmosphere. n.d.= no detectable N-O bond cleavage.

Supplementary Table 3. Relative stability of products following electron transfer from MPAA disulfide radical anion to model diglycine compound (1)

Pathway 1		Pathway 2		$\Delta G_{(1\text{-}2)}$ (eV)[a]
$GlyGlyN^{-}$ + xH_2O	$^{\bullet}OSH$ + xH_2O	$GlyGlyN^{\bullet}$ + xH_2O	^{-}OSH + xH_2O	
x = 0	x = 0	x = 0	x = 0	-0.49
0	1	1	0	-0.18
0	2	2	0	0.00
0	3	3	0	0.08
1	1	1	1	-0.25
1	2	2	1	-0.06
1	3	3	1	0.02
2	2	2	2	-0.09
2	3	3	2	-0.01
3	2	3	2	-0.24
3	2	5	0	-0.31
3	3	5	1	-0.14
4	3	5	2	-0.45
4	2	5	1	-0.67
4	1	5	0	-0.78
6	3	7	2	0.00
6	2	7	1	-0.22
7	2	7	2	-0.18
7	0	7	0	-0.35

[a]Relative energy at equilibrium geometry calculated for products of the electron transfer reaction from the MPAA disulfide radical anion to the model diglycine compound GlyGlyN-OCH_2CH_2SH (**1**) to yield a $GlyGlyN^{-}$ anion and $^{\bullet}OSH$ radical (Pathway 1), or $GlyGlyN^{\bullet}$ radical and ^{-}OSH anion (Pathway 2). Various numbers of explicit water molecules surrounding the products were included in the equilibrium geometry energy calculations where allowed. Values for $\Delta G_{(1\text{-}2)}$ reflect the energy at equilibrium geometry for the products of Pathway 1 relative to the products of Pathway 2.

Supplementary Table 4. Conditions for auxiliary removal from model dipeptide 2.[a]

	Reaction Conditions	10 min	24 h
**1**	Small molecule only	N.R.	N.R.
**2**	200 mM MPAA	N.R.	Reaction complete
**3**	50 mM H_2O_2	N.R.	N.R.
**4**	200 mM MPAA, 50 mM H_2O_2	Reaction complete	No change from 10 min.
**5**	200 mM MPAA, Chelex-treated[b]	N.R.	Minor progress
**6**	200 mM MPAA, 50 mM H_2O_2, Chelex-treated[b]	N.R.	~50% complete
**7**	200 mM MPAA (pH 8), degassed[c]	N.R.	Minor progress
**8**	200 mM 4-mercaptophenol	N.R.	N.R.
**9**	200 mM 4-mercaptophenol, 50 mM H_2O_2	N.R.	N.R.
**10**	200 mM 4-mercaptophenol, 200 mM MPAA	N.R.	N.R.

[a]Model dipeptide **2** was dissolved at 20 mM in 50:50 DMF: 100 mM Na_2HPO_4 and incubated at 25 °C. Various conditions were evaluated for auxiliary removal by monitoring starting material disappearance (R_f = 0.5) and 2-(tritylthio)ethanol appearance (R_f = 0.9) by TLC in 90:10 ethyl acetate: methanol. N.R. = no reaction. [b]Chelex-100 resin was swelled in water for 30 min. prior to use. DMF and buffer solutions were passed 2 times through the resin to remove divalent metal cations. [c]Buffer was subjected to three cycles of freeze-thaw degassing under Argon and the reaction kept under Argon atmosphere.

Supplementary Table 5. Summary of calculated and observed masses of proteins and peptides generated in this study.

Protein or peptide	Calculated [M] Da	Observed [M+H$^+$] Da
KAK^{aux}I	590.8	591.8
H4(1-14)^{aux}C(O)NHNH$_2$	1,362.8	1,363.8
H2B(117-125, A117C)^{photoaux}-C(O)OH	1,268.6	1,269.0
Ub(1-75)-MES	8,631.8	8,632.9 ± 1.8
SUMO-3(2-91)C47S-MES	10,443.7	10,445.8 ± 3.6
KAK$^{Ub(aux)}$I	9,080.1	9,082.3 ± 2.1
S-alkylated KAK$^{Ub(aux)}$I	9,151.3	9,154.0 ± 2.3
KAK^{Ub}I	9,004.1	9,004.8 ± 2.7
H4(15-102)A15C	10,070.8	10,075.3 ± 4.8
H4(1-14)$^{Su(C47S)(aux)}$-C(O)NHNH$_2$	11,665.1	11,666.9 ± 2.8
H4(A15C)$^{Su(C47S)(aux)}$	21,704.9	21,711.0 ± 5.9
H4(A15C)$^{Su(C47S)}$	21,627.7	21,634.0 ± 9.1
suH4	21,596.7	21,601.0 ± 6.2
H2B(1-116)-MES	12,933.8	12,936.0 ± 3.3
H2B(A117C)^{photoaux}	14,059.2	14,062.7 ± 2.8
H2B(A117C)^{aux}	13,924.1	13,925.8 ± 2.6
H2B(A117C)$^{Su(C47S)aux}$	24,225.6	24,230.9 ± 4.0
H2B(A117C)$^{Su(C47S)}$	24,149.6	24,153.1 ± 3.2

Supplementary Methods

General Methods

Rink-amide resin (0.46 mmol/g substitution) was purchased from Chem-Impex (Wood Dale, IL). 2-chlorotrityl chloride resin (1.33 mmol/g substitution) and Wang resin (0.4-0.6 mmol/g substitution) were purchased from EMD Millipore. Standard Fmoc-L-amino acids were purchased from AGTC Bioproducts (Wilmington, MA) or AnaSpec (Fremont, CA). All other chemical reagents were purchased from Sigma-Aldrich Chemical Company (St. Louis, MO) or Fisher Scientific (Pittsburgh, PA). DNA synthesis and gene sequencing were performed by Integrated DNA Technologies (Coralville, IA) and Genewiz (South Plainfeld, NJ), respectively. Plasmid mini-prep, PCR purification and gel extraction kits were purchased from Qiagen (Valencia, CA). Restriction enzymes were purchased from New England BioLabs (Ipswitch, MA) or Fermentas (Thermo Fisher Scientific, Philadelphia, PA). Chitin beads for purification of intein-CBD fusion proteins were purchased from New England BioLabs. Ni-NTA resin for purification of His_6-tagged proteins was purchased from Thermo Scientific (Waltham, MA). Solid phase peptide synthesis (SPPS) was performed on a Liberty Blue Automated Microwave Peptide Synthesizer (CEM Corporation, Matthews, NC). Analytical reversed-phase HPLC (RP-HPLC) was performed on a Varian (Palo Alto, CA) ProStar HPLC with a Grace-Vydac (Deerfield, IL) C4 or C18 column (5 micron, 150 x 4.6 mm) employing 0.1% TFA in water (A) and 90% CH_3CN, 0.1% TFA in water (B) as the mobile phases. Typical analytical gradients were 0-73% B over 30 min at a flow rate of 1 mL/min. Preparative scale purifications were conducted on a Grace-Vydac C4 or C18 column (10 micron, 250 x 22 mm) at a flow rate of 9 mL/min. Semi-preparative scale purifications were conducted on a Grace-Vydac C4 or C18 column (5 micron, 250 x 10 mm) at a flow rate of 3.5 mL/min. Mass spectrometric analysis was conducted on a Bruker (Billerica, MA) Esquire ESI-MS instrument. Analytical reversed-phase liquid chromatography-mass spectrometry (LC-ESI-MS) was performed on a Hewlett-Packard (Palo Alto, CA) 1100-series LC linked to the Bruker Esquire ESI-MS with an Agilent (Santa Clara, CA) Zorbax C18 column (3.5 micron, 100 x 2.1 mm) employing 5% CH_3CN, 1% AcOH in water (C) and CH_3CN, 1% AcOH (D) as the mobile phases. Typical analytical gradients were 0-100% D over 40 min at a flow rate of 0.2 mL/min. NMR spectra were recorded on Bruker Avance AV-300, AV-301, or AV-500 instruments. Circular dichroism measurements were performed on a JASCO (Easton, MD) J-720 spectropolarimeter. Size exclusion chromatography (SEC) was performed on a Bio-Rad Bio-Sil SEC 250 column (5 micron, 300 x 7.8 mm) at a flow rate of 1 mL/min.

Synthesis of the ligation auxiliary

The ligation auxiliary *O*-(2-(tritylthio)ethyl)hydroxylamine was prepared over 3 steps from *N*-hydroxyphthalimide as described previously.[1] ^{1}H NMR (300 MHz,CDCl3): δ 7.47-7.15 (15H), 5.29 (s, 2H), 3.54 (t, 2H, *J* = 6.24 Hz), 2.44 (t, 2H, *J* = 6.39 Hz). ^{13}C NMR (500 MHz, CDCl3): δ 144.86, 129.66, 127.93, 126.70, 73.70, 66.61, 30.81.

Synthesis of S-(2-nitrobenzyl)ethanethioate

4.04 g of 2-nitrobenzyl chloride (23.6 mmol) and 3.86 g of potassium carbonate, in separate flasks, were suspended in 24 mL and 48 mL of THF, respectively. To the stirring suspension of potassium carbonate was added 2 mL (30.5 mmol) of thioacetic acid. After 30 minutes the nitrobenzyl chloride solution was cannulated into this mixture. The reaction progress was followed by TLC. After 8 hours the starting material had been consumed and volatiles were stripped. The crude material was dissolved in methylene chloride, washed with bicarbonate solution, then water, and the organic phase dried *in vacuo* to give 5.87 g of solids, which were carried on to the next reaction without further purification.

Synthesis of (2-nitrobenzyl)thiol

Crude *S*-(2-nitrobenzyl)ethanethioate (5.87 g) was dissolved in 100 mL of methanol. To this solution was added 24 mL of concentrated hydrochloric acid (36%, 23.7 mmol), and the reaction allowed to proceed at 60 °C for 6 hours, after which TLC indicated complete hydrolysis of the starting material. The methanol

was removed *in vacuo*, and the resulting material purified by column chromatography with 90:10 hexane: ethyl acetate to give 2.30 g of (2-nitrobenzyl)thiol (75%, 2 steps). ^{1}H NMR (300 MHz,$CDCl_3$): δ 8.03 (d, 1H, *J* = 8.16), 7.61-7.44 (3H), 4.04 (d, 2H, *J* = 8.46), 2.17 (t, 1H, *J* = 8.46). ^{13}C NMR (301 MHz, $CDCl_3$): δ 147.89, 137.00, 133.74, 131.58, 128.26, 125.35, 26.45.

Synthesis of N-(2-((2-nitrobenzyl)thio)ethoxy)phthalimide

2.3 g of (2-nitrobenzyl)thiol (14.2 mmol) and 5.73 g of *N*-(2-bromoethoxy)phthalimide (21.3 mmol, prepared as previously described[1]) were dissolved in 25 mL DMSO. To this stirring solution was added 3 mL (21.3 mmol) of triethylamine, and the reaction allowed to proceed for 4 hours at room temperature. After TLC indicated complete consumption of (2-nitrobenzyl)thiol, 300 mL of water was added and the suspension extracted three times with methylene chloride. The organic layer was dried over anhydrous sodium sulfate and removed *in vacuo* to give crude product, which was recrystallized from a mixture of methylene chloride: hexane to give 3.74 g of *N*-(2-((2-nitrobenzyl)thio)ethoxy)phthalimide as pale yellow crystals (74%). ^{1}H NMR (500 MHz,$CDCl_3$): δ 7.99 (d, 1H, *J* = 8.15), 7.86-7.80 (4H), 7.56-7.44 (3H), 4.32 (t, 2H, *J* = 7.00), 4.21 (s, 2H), 2.87 (t, 2H, *J* = 7.00). ^{13}C NMR (500 MHz, $CDCl_3$): δ 163.45, 148.64, 134.65, 133.82, 133.16, 132.15, 128.85, 128.41, 125.50, 123.65, 77.56, 33.77, 29.39.

Synthesis of O-(2-((2-nitrobenzyl)thio)ethyl)hydroxylamine (3)

2 g (5.58 mmol) of *N*-(2-((2-nitrobenzyl)thio)ethoxy)phthalimide was dissolved in 100 mL chloroform. To this stirring solution was added 1.04 mL hydrazine hydrate (50% solution, 8.4 mmol), and the reaction monitored by TLC. After one hour the reaction was complete. Solids were removed by vacuum filtration and the organic layer washed three times with 200 mL water. Solvent was removed *in vacuo* to give 1.25 g of *O*-(2-((2-nitrobenzyl)thio)ethyl)hydroxylamine as a yellow oil (98%). ^{1}H NMR (300 MHz,$CDCl_3$): δ 7.99 (d, 1H, *J* = 7.91), 7.61-7.41 (3H), 5.43 (s, 2H), 4.13 (s, 2H), 3.80 (t, 2H, *J* = 6.41), 2.69 (t, 2H, *J* = 6.41). ^{13}C NMR (500 MHz, $CDCl_3$): δ 148.84, 134.18, 132.97, 131.98, 128.19, 125.33, 74.56, 33.62, 30.35. ESI-MS calculated *m/z* $[M+H]^+$ 229.1 Da, observed 228.9 Da.

Synthesis of Boc-KAKI-Rink-amide resin

The peptide H_2N-KAKI-$CONH_2$ was synthesized by microwave-assisted SPPS on a 0.1 mmol scale employing standard 9-fluorenylmethoxycarbonyl (Fmoc)-based N^{α}-deprotection chemistry. From Rink-amide resin (0.22 g, 0.46 mmol/g) each amino acid was coupled in 5.25 molar excess based on resin loading. Deprotection of the Fmoc group was achieved by treating resin with 20% piperidine in DMF for 65 s at 90 °C. Coupling reactions were undertaken for 2 min at 90 °C with a mixture of Fmoc-amino acid (0.53 mmol), *O*-(Benzotriazol-1-yl)-*N,N,N',N'*-tetramethyluronium hexafluorophosphate (HBTU, 0.51 mmol) and *N,N*-Diisopropylethylamine (DIEA, 1.1 mmol) in DMF. The Lys targeted for ubiquitylation was orthogonally protected with the 1-(4,4-dimethyl-2,6-dioxocyclohexylidine)-3-methylbutyl (ivDde) protecting group. The peptide was protected at the α-NH_2 position with the *tert*-butoxycarbonyl (Boc) group by reaction with di-*tert*-butyl dicarbonate (0.8 mmol) and DIEA (1.6 mmol) in DMF for 2 hours.

Synthesis of BocHN-H4(1-14)-2-chlorotrityl hydrazine resin

The peptide BocHN-SGRGKGGKGLG<u>K</u>GG-C(O)$NHNH_2$ corresponding to the first 14 N-terminal residues of the human histone H4 protein was synthesized by SPPS on a 0.25 mmol scale employing standard 9-fluorenylmethoxycarbonyl (Fmoc)-based N^{α}-deprotection chemistry. Briefly, 2-chlorotrityl hydrazine resin was prepared by reacting 2-chlorotrityl chloride resin (1.33 mmol/g) in a 10% solution of hydrazine in DMF at 30 °C for 30 min.[2] The reaction was repeated one time with fresh hydrazine solution. The resin was then treated with a 10% methanol in DMF solution for 10 min to cap any unreacted sites on the resin. The first amino acid, Gly, was coupled in 4 molar excess. The coupling reaction containing Fmoc-Glycine (1.0 mmol), *O*-(6-Chlorobenzotriazol-1-yl)-*N,N,N',N'*-tetramethyluronium hexafluorophosphate (HCTU, 0.95 mmol) , and DIEA (2.0 mmol) proceeded for 60 min at 30 °C. From glycinyl 2-chlorotrityl hydrazine resin each remaining amino acid was coupled in 5 molar excess based on resin loading. Deprotection of the Fmoc group was achieved by treating resin with

20% piperidine in DMF for 3 min at 75 °C. Coupling reactions were undertaken for 5 min at 75 °C with a mixture of Fmoc-amino acid (1.31 mmol), HBTU (1.28 mmol) and DIEA (2.75 mmol) in DMF. For Arg, an additional coupling reaction was performed for 25 min at 75 °C. The Lys at position 12 was orthogonally protected with the ivDde protecting group. The peptide was protected at the α-NH_2 position with Boc group by reaction with di-*tert*-butyl dicarbonate (2.0 mmol) and DIEA (4.0 mmol) in DMF for 2 hours.

Synthesis of BocHN-H2B(117-125, A117C)-wang resin

The peptide BocHN-CVTKYTSAK-C(O)OH corresponding to the last 9 C-terminal residues of the human histone H2B protein, with an Ala to Cys mutation at position 117, was synthesized by SPPS on a 0.1 mmol scale employing standard Fmoc-based N^{α}-deprotection chemistry. From Fmoc-Lys(Boc) wang resin (0.21 g, 0.4-0.6 mmol/g) each amino acid was coupled in 6 molar excess based on resin loading. Deprotection of the Fmoc group was achieved by treating resin with 20% piperidine in DMF for 3 min at 75 °C. Coupling reactions were undertaken for 5 min at 75 °C with a mixture of Fmoc-amino acid (0.6 mmol), *N,N′*-Diisopropylcarbodiimide (DIC, 0.6 mmol), Ethyl (hydroxyimino)cyanoacetate (Oxyma, 0.6 mmol) and DIEA (0.06 mmol) in DMF. Cys was coupled for 10 min at 50 °C. The Lys at position 120 was orthogonally protected with the ivDde protecting group. The peptide was Boc protected at the α-NH_2 position by reaction with di-*tert*-butyl dicarbonate (0.8 mmol) and DIEA (1.6 mmol) in DMF for 2 hours.

Attachment of the ligation auxiliary

Deprotection of the ivDde group was achieved by reacting resin bound peptide with a solution of 5% hydrazine in DMF for 5 min. This deprotection was repeated three times. The peptidyl resin was then coupled to bromoacetic acid (8-fold molar excess) with DIC (8-fold molar excess) in DMF for 45 min at room temperature. The coupling was repeated once. Subsequently, dry peptidyl resin was placed in a solution containing 9 equivalents of auxiliary (0.25 M in DMSO). The auxiliary *O*-(2-(tritylthio)ethyl)hydroxylamine (“aux”) was incubated with H_2N-KAKI-$CONH_2$ and BocHN-H4(1-14)-C(O)$NHNH_2$, and the photo-labile auxiliary *O*-(2-((2-nitrobenzyl)thio)ethyl)hydroxylamine (“photoaux”) was incubated with BocHN-H2B(117-125, A117C)-C(O)OH. The reactions were shaken for 24 hours at room temperature. Completion of the displacement was judged by test cleavage and subsequent ESI-MS analysis. Peptide was cleaved and deprotected by reaction of resin at 20 µL/mg with Reagent K (TFA: thioanisole: H_2O: phenol: 1,2-ethanedithiol 82.5:5:5:5:2.5 v/v)[3] for 1.5 hours at room temperature, then precipitated and washed 2 times with cold diethyl ether. Dry peptide was dissolved in RP-HPLC buffer A and purified by C18 preparative and semi-preparative RP-HPLC with a gradient of 0-25% B for $KAK^{aux}I$ and 0-50% B for H4(1-14)^{aux}-C(O)$NHNH_2$ and H2B(117-125, A117C)^{photoaux}-C(O)OH. This yielded 38% of the peptide-auxiliary conjugate $KAK^{aux}I$, 9% of the peptide-auxiliary conjugate H4(1-14)^{aux}-C(O)$NHNH_2$, and 25% of the peptide-auxiliary conjugate H2B(117-125, A117C)^{photoaux}-C(O)OH based on initial resin loading.

ESI-MS of $KAK^{aux}I$. Calculated *m/z* $[M+H]^+$ 591.4 Da, observed 591.8 Da.

ESI-MS of H4(1-14)^{aux}-$CONHNH_2$. Calculated *m/z* $[M+H]^+$ 1,363.6 Da, observed 1,363.8 Da.

ESI-MS of H2B(117-125, A117C)^{photoaux}-C(O)OH (**4**). Calculated *m/z* $[M+H]^+$ 1,268.6 Da, observed 1,269.0 Da.

Molecular cloning of SUMO-3(2-91)C47S, Ub(1-75), and H4(15-102)A15C

The plasmid pTXB1-Ub(1-76)-AvaDNAE-AAFN-His_6 containing the human ubiquitin gene, *ub(1-76)*, was a kind gift from the Muir lab at Princeton University,[4] and was used to generate the plasmid pTXB1-Ub(1-75)-AvaDNAE-AAFN-His_6, which lacks the C-terminal Gly of ubiquitin. The plasmid pTXB1-SUMO3(1-92)C47S, containing the human SUMO-3 gene *Smt3(1-92)* with a C47S mutation,[5] was used to generate the plasmid pTXB1-SUMO3(1-91)C47S, which lacks the C-terminal Gly of SUMO-3. The plasmid pET15b-His_6-[TEV]-H4, containing the human histone H4 gene with a Tobacco Etch Virus (TEV) protease cleavage sequence between the His_6 tag and the N-terminus of H4,[5] was used to generate the plasmid pET15b-His_6-[TEV]-H4(15-102)A15C, which lacks the first 14 residues of histone H4 and

bears the mutation A15C. Modified plasmids were prepared from their respective templates by site-directed mutagenesis (QuikChange kit, Agilent Technologies, Santa Clara, CA) with the following primers:

**Primer**	**DNA Sequence (5'- to -3')**
hSUMO-3(1-91)C47S-FP	ATCGACGTGTTCCAGCAGCAGACGGGATGCATCACGGGAGATGCACTAG TTGCC
hSUMO-3(1-91)C47S-RP	GGCAACTAGTGCATCTCCCGTGATGCATCCCGTCTGCTGCTGGAACACGT CGAT
hUb(1-75)-AvaDnaE-AAFN-H6-FP	CTGCACCTGGTACTCCGTCTCAGAGGTTGCCTGAGCTATGATACCGAAGT GCTG
hUb(1-75)-AvaDnaE-AAFN-H6-RP	CAGCACTTCGGTATCATAGCTCAGGCAACCTCTGAGACGGAGTACCAGG TGCAG
hH4(15-102)A15C-FP	GGGAATTC*CATATG*GAAAACCTGTACTTCCAGTGCAAACGTCACCGTAAA GTTCTG (*NdeI*)
hH4-RP	GCCCGC*GGATCC*TCAACCACCGAAACCGTACAGGGTACGACCC (*BamHI*)

The desired gene sequences were confirmed by sequencing with the T7 forward primer (Genewiz).

Overexpression and purification of Ub(1-75)-MES

E. coli BL21(DE3) cells were transformed with the plasmid pTXB1-Ub(1-75)-AvaDNAE-AAFN-His_6. Cells were grown in 3 L Luria-Bertani medium supplemented with 100 µg/mL of Ampicillin at 37 °C with shaking at 250 rpm until OD_{600} reached ~0.6. Overexpression was induced by the addition of 0.5 mM isopropyl *β*-D-1-thiogalactopyranoside (IPTG) and cells were grown for an additional 18 h at 16 °C. The cells were harvested by centrifugation at 7,000x*g* for 15 min. The cell pellet was resuspended in 45 mL lysis buffer: 50 mM Na_2HPO_4, 300 mM NaCl, 5 mM imidazole, pH 8. Cells were lysed by sonication then centrifuged at 20,000x*g* for 15 min. The lysate supernatant was passed through a 0.45 µm filter then applied to a 6 mL Ni-NTA column pre-equilibrated with lysis buffer. Proteins were bound to the column over a period of 1 h at 4 °C. The column was then washed with lysis buffer containing increasing concentrations of imidazole: 5 mM (5 Column Volumes, CV), 20 mM (5 CV), 50 mM (2.5 CV), 250 mM (5 CV). Pooled fractions containing Ub(1-75)-intein-His_6 were dialyzed into 2 L thiolysis buffer (100 mM Na_2HPO_4, 150 mM NaCl, 1 mM EDTA, 1 mM MESNa, pH 7.2) for 1 h at 4 ºC twice. Thiolysis buffer containing 1 M MESNa was added to the dialyzed fraction pool for a final concentration of 100 mM MESNa. The thiolysis reaction was incubated at 30 ºC for 18 h, after which Ub(1-75)-intein-His_6 was no longer present. The eluted Ub(1-75)-MES was purified by C18 preparative RP-HPLC employing a gradient of 30-60% B over 60 min. Fractions containing Ub(1-75)-MES were identified by ESI-MS. Typical yields were 5-7 mg/L of cell culture.
ESI-MS for Ub(1-75)-MES. Calculated *m/z* $[M+H]^+$ 8,632.8 Da, observed 8,632.9 ± 1.8 Da.

Non-denaturing expressed protein ligation of $KAK^{aux}I$ and Ub(1-75)-MES α-thioester

Ub(1-75)-MES α-thioester (0.038 µmol, 0.4 mg) and $KAK^{aux}I$ (0.38 µmol, 0.23 mg) were dissolved in 200 µL of reaction buffer containing 50 mM tris, 150 mM NaCl, 10 mM TCEP, 200 mM 4-mercaptophenylacetic acid (MPAA), pH 7.3-7.5. The reaction proceeded at 25 °C for a total of 48 h. After incubation the sample was treated with 50 mM TCEP at 4 °C for 30 min, acidified to pH ~3 with formic acid, and extracted once with diethyl ether to remove the majority of MPAA. The sample was analyzed by LC-ESI-MS employing a gradient of 0-100% D over 40 min.

Generation of $KAK^{Ub(aux)}I$ for N-O bond cleavage tests by expressed protein ligation of $KAK^{aux}I$ and Ub(1-75)-MES α-thioester

In a typical reaction, purified $KAK^{aux}I$ (0.35 mg, 0.6 µmol) and Ub(1-75)-MES (0.12 µmol) were dissolved in 300 µL of a buffer consisting of 6 M Gn-HCl, 100 mM Na_2HPO_4, and 10 mM TCEP, pH 7.3.

Ligation proceeded with gentle shaking at 25 °C for 24 h. Ligation product was purified by C18 analytical RP-HPLC employing a gradient of 30-50% B over 30 min. Typical yields were 60-80% of purified product.
ESI-MS of $KAK^{Ub(aux)}I$. Calculated *m/z* $[M+H]^+$ 9,081.1 Da, observed 9,082.3 ± 2.1 Da.

Requirements for auxiliary removal under non-denaturing conditions
$KAK^{Ub(aux)}I$ was subjected to various assay conditions to evaluate the reaction components necessary for auxiliary removal via N-O bond cleavage. $KAK^{Ub(aux)}I$ (30 nmol, ~0.3 mg) was dissolved in 300 μL buffer and incubated in a 1.5 mL microcentrifuge tube for 48 h at 25 °C, after which the sample was combined with an equal volume 200 mM TCEP, 6 M Gn-HCl, 100 mM Na_2HPO_4, pH 7.3 and incubated at 4 °C for 30 min. The sample was then treated with 50 mM TCEP at 4 °C for 30 min, acidified to pH ~3 with formic acid, and extracted once with diethyl ether to remove the majority of MPAA. The sample was analyzed by LC-ESI-MS employing a gradient of 0-100% D over 40 min. Percent yield was determined by the percent auxiliary-free ligation product $KAK^{Ub}I$ relative to other species present, determined by ESI-MS signal intensity for R_t = 14-17 min.

Time course of auxiliary removal under Condition 1
Auxiliary-containing ligation product (30 nmol, ~0.3 mg) was dissolved in 300 μL of 200 mM MPAA, 100 mM Na_2HPO_4, pH 7.3, and incubated in a 1.5 mL microcentrifuge tube at 25 °C for up to 48 h.

Time course of auxiliary removal under Condition 2
Auxiliary-containing ligation product (10 nmol, ~0.1 mg) was dissolved in 100 μL of 200 mM MPAA, 100 mM Na_2HPO_4, pH 7.3, and incubated in a 1.5 mL microcentrifuge tube at 25 °C for up to 24 h.

Time course of auxiliary removal under Condition 3
Auxiliary-containing ligation product (10 nmol, ~0.1 mg) was dissolved in 100 μL of 200 mM MPAA, 100 mM Na_2HPO_4, pH 7.3. To this was added 0.51 μL of a 30% (9.8 M) solution of H_2O_2 for a final concentration of 50 mM H_2O_2. The reaction was incubated in a 1.5 mL microcentrifuge tube at 25 °C for up to 8 h.

Analysis of time-points for auxiliary removal
An equal volume of 200 mM TCEP, 6 M Gn-HCl, 100 mM Na_2HPO_4, pH 7.5 was added to each sample, which was then incubated at 4 °C for 30 min. The solution pH was adjusted to 2-3 with formic acid, and one extraction with 0.5 mL diethyl ether was performed. Reaction progress was analyzed by LC-ESI-MS employing a gradient of 0-100% D over 40 min. Percent yield was determined by the percent auxiliary-free ligation product $KAK^{Ub}I$ relative to other species present, determined by ESI-MS signal intensity for R_t = 14-17 min.

Dependence of auxiliary removal on reduced thiol
The disulfide form of MPAA was generated by dissolving MPAA at 200 mM in 100 mM Na_2HPO_4, pH 8.0, in a tube with headspace filled with air equal to 10 times the liquid volume. The solution was shaken vigorously at room temperature for 48 h, after which time the pH was lowered to 1-2 with HCl causing the MPAA species to precipitate. Acetonitrile was added until the precipitate dissolved (~40% of total volume). The MPAA disulfide, 2,2'-(disulfanediylbis(4,1-phenylene))diacetic acid, was purified by C18 preparative RP-HPLC employing a gradient of 30-80% B over 60 min. ^1H NMR (301 MHz, CD_3CN): δ 7.16 (d, 4H, *J* = 8.34 Hz), 6.92 (d, 4H, *J* = 8.34 Hz), 3.26 (s, 4H). Negative mode ESI-MS calculated *m/z* $[M-H]^-$ 333.4 Da, observed 333.9 Da.

Thiyl radical detection by oxidation of NADH
To investigate the formation of thiyl radicals under auxiliary-removal conditions, reduced nicotinamide adenine dinucleotide (NADH) was dissolved at a final concentration of 40 mM in solutions containing

200 mM thiol and 100 mM Na_2HPO_4, pH 7.3. The solutions were incubated at 25 °C, protected from light. Absorbance at 340 nm was measured at various time points. A decrease in absorbance, due to oxidation of NADH, suggested the presence of thiyl radicals.[6]
To further confirm the ability of aromatic thiols to perform single-electron transfer reactions, solutions were prepared containing 200 mM of each thiol and 100 mM Na_2HPO_4, pH 7.3, and these solutions were added to dry aliquots of the radical indicator, methyl viologen (MV^{2+}), for a final concentration of 20 mM MV^{2+}. Both MV^{2+} and its two-electron reduction product (MV°) have absorbance maxima less than 400 nm. The single-electron reduction product $MV^{\cdot+}$, however, has a strong characteristic absorbance at 605-610 nm.[7] The resulting deep purple color was observed immediately upon mixing methyl viologen with the aromatic thiol solutions, but no color change occurred with aliphatic thiols, even after 24 h.

EPR experiments
A 200 mM MPAA stock solution was prepared in DMF. Aliquots from the stock solution were diluted with DMF and 50 mM Na_2HPO_4 at pH 7.5 to a final 1:1 water-DMF mixture. Then, 100 mM 5,5-Dimethyl-1-pyrroline *N*-oxide (DMPO) was added, the samples were vortexed, transferred to a flat cell and EPR spectra were recorded at room temperature on a Bruker EMX spectrometer equipped with a high sensitivity cavity and operating at 9.65 GHz and 100 KHz field modulation. MPAA alkylation was performed by incubating 50 mM MPAA with 70mM 2-Iodoacetamide in 50 mM Na_2HPO_4 at pH 7.5, in a 1:1 water-DMF for 1.5 h at 25 ^oC before DMPO addition. Parallel controls by pre-incubating 50 mM MPAA alone in 50 mM Na_2HPO_4 at pH 7.5, in a 1:1 water-DMF for 1.5 h at 25 ^oC before DMPO addition were also run. Computer simulation was performed using Winsim program from P.E.S.T.[8]

Detection of DMPO adducts by mass spectrometry
Reactions were prepared identically to the EPR experiments. Aliquots of 5 uL were analyzed by LC-ESI-MS employing a gradient of 30-100% D over 10 min. Adduct spectra were generated by averaging the ESI-MS signal between 6-7 min. R_t = 3-5 min for DMPO; R_t = 6-7 min for the DMPO-OH and DMPO-MPAA adducts; R_t = 9-10 min for MPAA. The DMPO-MPAA or DMPO-OH adduct radical may exist in the radical, oxidized, and reduced states when observed by ESI-MS,[9] and the oxidized form appeared to be most prevalent for reactions of DMPO with H_2O_2 or MPAA.

Expected products of DMPO reaction with hydroxyl radical:

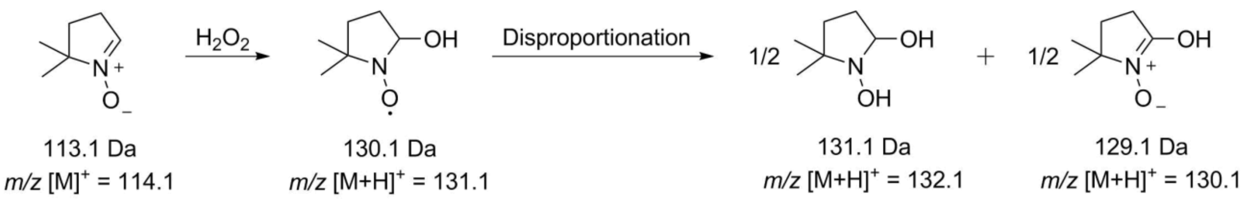

Expected products of DMPO reaction with MPAA thiyl radical:

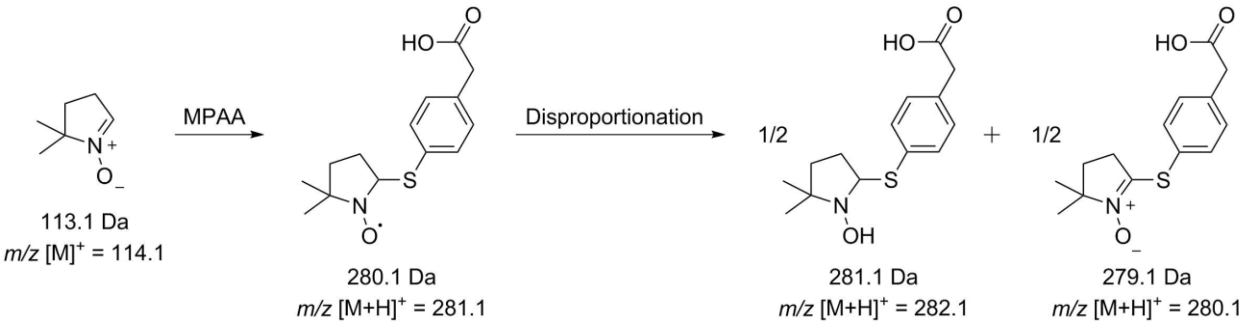

MPAA-mediated auxiliary removal in the presence of a radical quencher

KAK$^{Ub(aux)}$I (50 µg, ~5 nmol) was dissolved in 58 µL of 100 mM MPAA, 100 mM Na_2HPO_4, pH 7.3 containing 1 M of DMPO and incubated at 25 °C for 24 h. The reaction was then treated with TCEP as above, acidified to pH ~3 with formic acid, and analyzed by LC-ESI-MS employing a gradient of 0-100% D over 40 min. Percent yield was determined by the percent auxiliary-free ligation product KAK^{Ub}I relative to other species present, determined by ESI-MS signal intensity for R_t = 14-17 min.

***S*-alkylation of KAK$^{Ub(aux)}$I and MPAA-mediated auxiliary removal from the alkylated species**

Alkylation of the pendant thiol in the ligation product KAK$^{Ub(aux)}$I was performed under conditions established for selectively alkylating protein sulfhydryl groups.[10] Briefly, 1.2 mg of the ligation product was dissolved in 280 µL of alkylation buffer containing 1 M HEPES, 4 M Gn-HCl, and 10 mM Methionine at pH 7.8. To this was added 14 µL of 1 M DTT in alkylation buffer. The resulting mixture was incubated at 25 °C for 20 min after which time 31 µL of a 1 M solution of *N*-(2-chloroethyl)-*N*,*N*-dimethylammonium chloride in alkylation buffer was added. The reaction was incubated at 25 °C for 2 h. Next, an additional 3.4 µL of 1 M DTT solution was added followed by a 30 min incubation at 25 °C to reduce any residual disulfides. Finally, an additional 31 µL of *N*-(2-chloroethyl)-*N*,*N*-dimethylammonium chloride in alkylation buffer was added and the reaction incubated for 2 h to ensure complete alkylation of all thiol groups. The reaction mixture was quenched with 10 µL of β-mercaptoethanol and purified by C18 analytical RP-HPLC employing a gradient of 30-50% B over 30 min. *S*-alkylated KAK$^{Ub(aux)}$I was characterized by ESI-MS. Calculated *m/z* $[M+H]^+$ 9,152.3 Da, observed 9,154.0 ± 2.3 Da.

To evaluate whether MPAA-mediated auxiliary removal is dependent on the pendent thiol of the auxiliary, *S*-alkylated KAK$^{Ub(aux)}$I (0.135 mg) was dissolved in 0.135 mL buffer containing 200 mM MPAA, 100 mM Na_2HPO_4, pH 7.3, and incubated at 25 °C for 48 h. The reaction was reduced with TCEP and analyzed by LC-ESI-MS as above. The expected product KAK^{Ub}I was observed. Calculated *m/z* $[M+H]^+$ 9,005.1 Da, observed 9,004.8 ± 2.7 Da.

Effect of superoxide on N-O bond cleavage in KAK$^{Ub(aux)}$I

To rule out superoxide-mediated N-O bond cleavage, KAK$^{Ub(aux)}$I was subjected to the xanthine oxidase/hypoxanthine reaction. Molecular oxygen is reduced to superoxide as hypoxanthine is oxidized to xanthine, and subsequently to uric acid.[11] KAK$^{Ub(aux)}$I was dissolved at 0.04 mM in 100 µL of 50 mM Na_2HPO_4, pH 7.5 in the presence of 0.08, 0.16, or 0.32 mM hypoxanthine and 0.14 u/mL xanthine oxidase (Sigma-Aldrich) at 25 °C for 24 h. Progress of the enzymatic reaction was monitored by an increase in the absorbance of uric acid at 290 nm.[12] Cleavage of the N-O bond was evaluated by LC-ESI-MS employing a gradient of 0-100% D over 40 min.

Synthesis of 2-((2-(tritylthio)ethoxy)amino)acetamide

To a stirring solution of *O*-(2-(tritylthio)ethyl)hydroxylamine (1.74 g, 5.19 mmol) in anhydrous DMF (15 mL) under a flow of Argon was added a solution containing iodoacetamide (482 mg, 2.60 mmol) in anhydrous DMF (10 mL). To this mixture was added DIEA (0.91 mL, 5.19 mmol) and the reaction stirred at room temperature until TLC revealed that starting material had been consumed (~20 h). Ice-cold, basified H_2O (250 mL, pH ~8) was added to the reaction mixture in DMF, causing a cloudy, white precipitate to form. The DMF/H_2O solution was extracted with ethyl acetate until no further precipitate was visible (~500 mL), then the ethyl acetate layer washed 3 times with an equal volume of basified H_2O. Solvent was removed *in vacuo* to yield crude product, which was purified by silica gel (70-230 mesh) column chromatography (10:90 hexane: ethyl acetate, 0.1% triethylamine) to give compound 2-((2-(tritylthio)ethoxy)amino)acetamide (0.88 g, 86%). ^{1}H NMR (301 MHz, $CDCl_3$): δ 7.43-7.19 (15H), 6.35 (s, 1H), 5.77 (t, 1H, *J* = 5.01 Hz), 5.43 (s, 1H), 3.56 (t, 2H, *J* = 6.46 Hz), 3.46 (d, 2H, *J* = 5.15 Hz), 2.44 (t, 2H, *J* = 6.46 Hz). ^{13}C NMR (500 MHz, $CDCl_3$): δ 172.59, 144.69, 129.61, 127.97, 126.77, 72.30, 66.77, 54.85, 30.90. ESI-MS calculated *m/z* $[M+Na]^+$ 415.5 Da, observed 415.2 Da.

Synthesis of 2-acetamido-N-(2-amino-2-oxoethyl)-N-(2-(tritylthio)ethoxy)acetamide (2)
A solution of 2-((2-(tritylthio)ethoxy)amino)acetamide (900 mg, 2.29 mmol), 1-hydroxybenzotriazole (930 mg, 6.88 mmol), *N*-acetylglycine (806 mg, 6.88 mmol), and DIEA (1.2 mL, 6.88 mmol) in 23 mL DMF was prepared over an ice bath. To this stirring solution was added *N,N'*-dicyclohexylcarbodiimide (1.42 g, 6.88 mmol). The reaction was stirred on ice for 90 min, then allowed to warm up to room temperature overnight. The reaction proceeded for 24 h, at which time TLC revealed that starting material had been consumed. The white precipitate formed over the course of the reaction was removed by vacuum filtration. To the filtrate was added ice-cold H_2O (500 mL) causing a cloudy, white precipitate to form. The DMF/H_2O solution was extracted with ethyl acetate until no further precipitate was visible (~1.5 L), then the ethyl acetate layer washed 3 times with an equal volume of H_2O. Solvent was removed *in vacuo* to yield crude product, which was purified by silica gel (70-230 mesh) column chromatography (90:10 ethyl acetate: methanol, 0.1% triethylamine) to give model dipeptide **2**, 2-acetamido-*N*-(2-amino-2-oxoethyl)-*N*-(2-(tritylthio)ethoxy)acetamide (0.74 g, 66%). ^{1}H NMR (500 MHz, $CDCl_3$): δ 7.43-7.21 (15H), 6.18 (t, 1H), 6.09 (s, 1H), 5.38 (s, 1H), 4.13 (s, 2H), 4.09 (d, 2H, J = 4.75 Hz), 3.53 (t, 2H, J = 6.55 Hz), 2.52 (t, 2H, J = 6.60 Hz), 2.03 (s, 3H). ^{13}C NMR (500 MHz, DMF-D_7): δ 174.12, 171.74, 170.41, 146.67, 131.47, 130.13, 128.89, 81.13, 74.71, 68.87, 52.06, 43.37, 23.90. ESI-MS calculated *m/z* $[M+Na]^+$ 514.6 Da, observed 514.3 Da.

Characterization of the cleaved auxiliary species
Model dipeptide **2** was dissolved at 40 mM in 2.5 mL DMF. To this solution was added 2.5 mL of 100 mM Na_2HPO_4, pH 7.3 containing 400 mM MPAA. The reaction was incubated at 25 °C for a total of 24 h. Reaction progress was monitored by TLC in 90:10 ethyl acetate: methanol and evaluated by disappearance of starting material and appearance of a spot migrating near the solvent front (R_f = 0.9), attributed to the cleaved auxiliary bearing the trityl group. At the end of 24 h, an equal volume of 100 mM Na_2HPO_4, pH 7.3 was added. A white precipitate formed and was collected by centrifugation, then washed 2 times with buffer. The dried precipitate was dissolved in ethyl acetate and passed through a silica plug with 100% ethyl acetate. Solvent was removed *in vacuo* to yield a single spot by TLC, identified as 2-(tritylthio)ethanol. ^{1}H NMR (301 MHz, $CDCl_3$): δ 7.31-7.19 (15H), 3.38 (t, 2H, J = 6.19 Hz), 2.48 (t, 2H, J = 6.19 Hz). ESI-MS calculated *m/z* $[M+Na]^+$ 343.4 Da, observed 343.3 Da.

One-pot ligation and auxiliary removal
Ub(1-75)-α-thioester (0.019 μmol, 0.2 mg) and KAK^{aux}I (0.19 μmol, 0.12 mg) were dissolved in 100 μL of reaction buffer containing 50 mM tris, 150 mM NaCl, 0.5 mM MPAA, pH 7.3, and incubated at 25 °C for 24 h. Then, 200 uL of buffer containing 300 mM MPAA, 50 mM tris, 150 mM NaCl, pH 7.3, was added and the reaction incubated at 25 °C for 24 h, for a total of 48 h reaction time. After incubation the sample was treated with 50 mM TCEP at 4 °C for 30 min, acidified to pH ~3 with formic acid, and extracted one time with diethyl ether to remove the majority of MPAA. The sample was analyzed by LC-ESI-MS employing a gradient of 0-100% D over 40 min.

Overexpression and purification of SUMO-3(2-91)C47S-MES
E. coli BL21(DE3) cells were transformed with the plasmid pTXB1-SUMO-3(2-91)C47S. Cells were grown in 6 L Luria-Bertani medium supplemented with 100 µg/mL of Ampicillin at 37 °C with shaking at 250 rpm until OD_{600} ~0.6-0.8. Overexpression of the desired fusion proteins was induced by the addition of 0.3 mM IPTG and cells were grown for an additional 4 h at 25 °C. The cells were harvested by centrifugation at 7,000x*g* for 15 min. The cell pellet was resuspended in lysis buffer: PBS, pH 7.2 containing 1 mM 2-mercaptoethanesulfonic acid sodium salt (MESNa). Cells were lysed by sonication then centrifuged at 20,000x*g* for 15 min. The lysate supernatant was passed through a 0.45 µm filter then applied to a 30 mL chitin column pre-equilibrated with lysis buffer. Proteins were bound to the column over a period of 12 h at 4 °C. The column was then washed with 20 column volumes (CV) of lysis buffer followed by 2 CV of PBS, pH 7.75. SUMO-3(2-91)C47S-MES was cleaved from its intein-CBD fusion

by incubation with 1.5 CV of PBS, pH 7.75 containing 100 mM MESNa for 72 h at 4 °C. The eluted α-thioester was purified by C18 preparative RP-HPLC employing a gradient of 30-60% B over 60 min. Fractions containing the desired thioester were identified by ESI-MS. We observed that the N-terminal Met of SUMO-3 is consistently processed *in vivo*, leading to the SUMO-3(2-91)-α-thioester product. Typical yield is 4-5 mg/L of cell culture.
ESI-MS for SUMO-3(2-91)C47S-MES. Calculated *m/z* $[M+H]^+$ 10,444.7 Da, observed 10,445.8 ± 3.6 Da.

Expressed protein ligation of H4(1-14)$^{(aux)}$-CONHNH$_2$ and SUMO-3(2-91)C47S-MES
Purified H4(1-14)$^{(aux)}$-CONHNH$_2$ (15.6 mg, 11.6 µmol) and SUMO-3(2-91)C47S-MES (20.1 mg, 1.9 µmol) were dissolved in 7.2 mL of a buffer consisting of 6 M Gn-HCl, 100 mM Na_2HPO_4, and 10 mM TCEP, pH 7.3. Ligation proceeded with gentle shaking at 25 °C for 24 h. Ligation product was purified by C18 preparative RP-HPLC employing a gradient of 25-50% B over 60 min to give 12.6 mg (56%).
ESI-MS of H4(1-14)$^{Su(C47S)(aux)}$-CONHNH$_2$. Calculated *m/z* $[M+H]^+$ 11,666.1 Da, observed 11,666.9 ± 2.8 Da.

Overexpression and purification of TEV protease
E. coli BL21(DE3) cells containing the plasmid pRK793-His$_6$-TEV[5] were grown in 1 L LB supplemented with 100 µg/mL of Ampicillin at 37 °C with shaking at 250 rpm until OD_{600} ~0.6. Overexpression was induced by the addition of 0.3 mM IPTG and cells were grown for an additional 6 h at 25 °C. The cells were harvested by centrifugation at 7,000x*g* for 15 min. The cell pellet was resuspended in 15 mL lysis buffer: 20 mM tris, 50 mM NaCl, pH 7.2. Cells were lysed by sonication then centrifuged at 20,000x*g* for 15 min. The lysate supernatant was passed through a 0.45 µm filter then applied to ~5 mL Ni-NTA column pre-equilibrated with lysis buffer. Proteins were bound to the column over a period of 3 h at 4 °C. The column was then washed thoroughly with lysis buffer containing 200 mM imidazole. His$_6$-TEV was eluted with lysis buffer containing 500 mM imidazole, and dialyzed into 4 L of 20 mM tris, 50 mM NaCl, 1 mM DTT, pH 7.5 for 18 h at 4 °C.

Overexpression and purification of H4(15-102)A15C
E. coli BL21(DE3) cells containing pET15b-His$_6$-[TEV]-H4(15-102)A15C were grown in 3 L Luria-Bertani medium supplemented with 100 µg/mL of Ampicillin at 37 °C with shaking at 250 rpm until OD_{600} reached ~0.6. Overexpression was induced by the addition of 0.3 mM IPTG and cells were grown for an additional 1.5 h at 37 °C. The cells were harvested by centrifugation at 7,000x*g* for 15 min. H4(15-102)A15C was purified using a previously established protocol.[5] Cells were resuspended in wash buffer (20 mM tris, 200 mM NaCl, 1 mM EDTA, 1 mM 2-mercaptoethanol, pH 7.5, 1% triton X-100) and lysed by sonication on ice. Inclusion bodies were pelleted by centrifugation at 20,000x*g* for 20 min and washed twice with wash buffer. Inclusion bodies were then dissolved in extraction buffer (6 M Gn-HCl, 20 mM tris, 1 mM 2-mercaptoethanol, pH 7.5) and applied to Ni-NTA resin. Column binding proceeded overnight at 4 °C, after which the resin was washed with 10 CV extraction buffer containing 25 mM imidazole. The protein was eluted with 3 x 1 CV extraction buffer containing 400 mM imidazole, then dialyzed into water containing 1 mM DTT. After dialysis, 10x cleavage buffer was added for final concentrations of 50 mM tris, 1 mM EDTA, 10 mM DTT, 10 mM L-cysteine, pH 6.9. Purified TEV protease was added to ~20% of the final volume, and the cleavage reaction proceeded overnight at 37 °C. The reaction was then dialyzed back into extraction buffer, incubated overnight at 4 °C with Ni-NTA resin to remove the his-tagged TEV protease and cleaved H4 N-terminal tail, and the column flow-through containing H4(15-102)A15C purified by C4 preparative RP-HPLC employing a gradient of 40-70% B over 60 min. Typical yields were 3-4 mg/L of cell culture.
ESI-MS of H4(15-102)A15C. Calculated *m/z* $[M+H]^+$ 10,071.8 Da, observed 10,075.3 ± 4.8 Da.

Expressed protein ligation of H4(1-14)$^{Su(C47S)(aux)}$-C(O)NHNH$_2$ and H4(15-102)A15C

Ligation was accomplished by first converting the C-terminal hydrazide of H4(1-14)$^{Su(C47S)(aux)}$-C(O)NHNH$_2$ to an acyl azide with $NaNO_2$ via the diazotization reaction, as described previously.[2] Subsequent addition of MPAA served to both quench the remaining $NaNO_2$ and generate a highly reactive C-terminal thioester for the ligation reaction. Purified H4(1-14)$^{Su(C47S)(aux)}$-C(O)NHNH$_2$ (1.7 mg, 0.15 μmol) was dissolved at 1 mM in 200 mM Na_2HPO_4, 6 M Gn-HCl, pH 3, and kept at -20 °C for a minimum of 20 min. To this solution was added 4.5 μL of a 500 mM solution of $NaNO_2$ in water. The reaction was briefly mixed, then kept at -20 °C for 15 min. Then, a solution of H4(15-102)A15C (3 mg, 0.3 μmol) dissolved at 1.25 mM in 200 mM Na_2HPO_4, 6 M Gn-HCl, 200 mM MPAA, pH 6.5, was added to the reaction. The mixture was allowed to come to room temperature, and the pH adjusted with 3 M NaOH to 6.8-7.0 in order to form the MPAA α-thioester. The ligation reaction proceeded with gentle shaking at 25 °C for 24 h. Ligation product was purified by C4 semi-preparative RP-HPLC employing a gradient of 30-70% B over 45 min to give 2.1 mg (66%). ESI-MS of H4(A15C)$^{Su(C47S)(aux)}$. Calculated *m/z* $[M+H]^+$ 21,704.9 Da, observed 21,711.0 ± 5.9 Da.

MPAA-mediated auxiliary removal from H4(A15C)$^{Su(C47S)(aux)}$

Purified H4(A15C)$^{Su(C47S)(aux)}$ (1.4 mg, 64 nmol) was dissolved in 7 mL of 200 mM MPAA, 100 mM Na_2HPO_4, pH 7.3, and incubated in a 15 mL conical tube at 25 °C for 24 h. After incubation, the reaction volume was reduced with a 10,000 MWCO spin concentrator (GE Healthcare Life Sciences, Pittsburgh, PA) and buffered TCEP, pH 7.3 was added to a concentration of 50 mM and the reaction incubated at 4 °C for 30 min. Ligation product was purified by C4 analytical RP-HPLC employing a gradient of 30-70% B over 30 min. ESI-MS of H4(A15C)$^{Su(C47S)}$. Calculated *m/z* $[M+H]^+$ 21,628.7 Da, observed 21,634.0 ± 6.1 Da.

Desulfurization of H4(A15C)$^{Su(C47S)}$

Purified H4(A15C)$^{Su(C47S)}$ (1.4 mg, 65 nmol) was dissolved at 65 μM in 100 mM Na_2HPO_4, 6 M Gn-HCl, 500 mM TCEP, 100 mM MESNa, pH 7.5. To this solution was added 2-methyl-2-propanethiol to a concentration of 280 mM and radical initiator 2,2'-Azobis[2-(2-imidazolin-2-yl)propane]dihydrochloride (VA-044) to a concentration of 10 mM. The reaction was incubated at 37 °C for 24 h, and the product purified by C4 analytical RP-HPLC employing a gradient of 30-70% B over 30 min to give 0.6 mg (41%). ESI-MS of suH4. Calculated *m/z* $[M+H]^+$ 21,596.7 Da, observed 21,601.0 ± 6.2 Da.

Overexpression and purification of H2B(1-116)-MES

E. coli BL21(DE3) cells were transformed with the plasmid pTXB1-H2B(1-116)-GyrA-His$_6$.[13] Cells were grown in 4 L Luria-Bertani medium supplemented with 100 µg/mL of Ampicillin at 37 °C with shaking at 250 rpm until OD_{600} reached ~0.6. Overexpression was induced by the addition of 0.35 mM IPTG and cells were grown for an additional 4 h at 25 °C. The cells were harvested by centrifugation at 7,000x*g* for 15 min. The cell pellet was resuspended in 40 mL of 1X PBS. Cells were lysed by sonication then centrifuged at 20,000x*g* for 15 min. The pellet was washed with 40 mL of 50 mM tris, 200 mM NaCl, 1% triton X-100, pH 7.5. The pellet was then dissolved in 40 mL extraction buffer: 6 M urea, 10 mM tris, 200 mM NaCl, pH 7.5. After centrifugation at 20,000x*g* for 15 min the supernatant was applied to a 5 mL Ni-NTA column pre-equilibrated with extraction buffer. Proteins were bound to the column over a period of 1 h at 4 °C. The column was then washed with extraction buffer containing increasing concentrations of imidazole: 20 mM (10 CV), 50 mM (10 CV), 500 mM (5 CV). Pooled fraction containing H2B(1-116)-GyrA-His$_6$ were dialyzed into 2 L thiolysis buffer (10 mM tris, 200 mM NaCl, 2 M urea, 1 mM MESNa, 1 mM EDTA, pH 7.5) for 1 h at 4 °C twice. Thiolysis buffer containing 1 M MESNa was added to the dialyzed fraction pool for a final concentration of 100 mM MESNa. The thiolysis reaction was incubated at 4 °C for 24 h, after which H2B(1-116)-GyrA-His$_6$ was no longer present. The reaction containing H2B(1-116)-MES was dialyzed into 2 L of 4 M Gn-HCl, 50 mM Na_2HPO_4, pH 7.5, for 1 h at 4 °C twice, then applied to 5 mL Ni-NTA resin pre-equilibrated in the same buffer. The byproduct GyrA-His$_6$ was bound to the column over a period of 30 min at 4 °C. The product H2B(1-116)-MES was eluted and

purified by C18 preparative RP-HPLC employing a gradient of 35-65% B over 60 min. Fractions containing H2B(1-116)-MES were identified by ESI-MS. Typical yields were 1-2 mg/L of cell culture. ESI-MS of H2B(1-116)-MES. Calculated *m/z* $[M+H]^+$ 12,933.8 Da, observed 12,936.0 ± 3.3 Da.

Expressed protein ligation of H2B(117-125, A117C)^{photoaux} (4) and H2B(1-116)-MES α-thioester

H2B(1-116)-MES α-thioester (0.31 μmol, 4 mg) and peptide **4** (6.6 μmol, 8.5 mg) were dissolved in 500 μL of reaction buffer containing 6 M Gn-HCl, 100 mM Na_2HPO_4, 10 mM EDTA, 5 mM TCEP, pH 7.5. The reaction proceeded at 25 °C for 6 h. After incubation the sample was treated with 50 mM TCEP at 4 °C for 30 min. Ligation product was purified by C4 analytical RP-HPLC employing a gradient of 30-65% B over 30 min, and yielded 2.6 mg (60%). ESI-MS of H2B(A117C)^{photoaux} (**5**). Calculated *m/z* $[M+H]^+$ 14,059.2 Da, observed 14,062.7 ± 2.8 Da.

Refolding and photo-deprotection of H2B(A117C)^{photoaux} (5)

5 was dissolved at 0.4 mg/mL in 6 M Gn-HCl, 50 mM Na_2HPO_4, pH 7.5, and 1.5 mL was dialyzed into 1 L of 50 mM Na_2HPO_4, pH 7.5, at 4 °C, protected from light, for incubations of 3, 12, and 3 h, with fresh buffer for each incubation. The presence of secondary structure due to folding was confirmed by circular dichroism. To this solution, 3X photo-deprotection buffer (50 mM Na_2HPO_4, 12 mM semicarbazide, 15 mM ascorbic acid, 1.5 mM DTT, pH 6) was added to a final concentration of 1X. The sample was placed in a quartz cuvette with 1 cm path length and irradiated at 365 nm for 3.5 h with a hand-held 4 W Hg lamp (UVP, Upland, CA; measured irradiance = 1.8 mW cm^{-2}) 2 cm from light source. Irradiation effected complete removal of the *o*-nitrobenzyl group and unmasking of the auxiliary thiol. Deprotection was confirmed by LC-ESI-MS analysis employing a gradient of 0-100% D over 15 min. ESI-MS of H2B(A117C)^{aux} (**6**). Calculated *m/z* $[M+H]^+$ 13,924.1 Da, observed 13,925.8 ± 2.6 Da.

Expressed protein ligation of H2B(A117C)^{aux} (6) and SUMO-3(2-91)C47S-MES-α-thioester

Photo-deprotected **6** was dialyzed into 1 L of 50 mM Na_2HPO_4, 0.5 mM TCEP, pH 7.5, at 4 °C for incubations of 3 and 12 h. Separately, SUMO-3(2-91)C47S-MES was dissolved at 1.45 mg/mL in 50 mM Na_2HPO_4, pH 7.5, 1% DMF. The solution containing **6** (~ 11.6 uM) was combined 3:1 with the SUMO-3(2-91)C47S-MES solution for final concentrations of 8.6 uM H2B(1-117)^{aux} and 26.2 uM SUMO-3(2-91)C47S-MES (3 eq). To this mixture was added 200 mM TCEP, 20 mM MPAA, 50 mM Na_2HPO_4, pH 7.5, to final concentrations of 2 mM TCEP and 0.2 mM MPAA. The reaction was incubated under Ar at 22 °C for 48 h to give H2B(A117C)$^{Su(C47S)aux}$ (**7**) in 30-60% yield. Ligation was visualized by SDS-PAGE and confirmed by LC-ESI-MS analysis employing a gradient of 0-100% D over 15 min. Yield was determined by SDS-PAGE band intensity relative to BSA standards of known concentration. ESI-MS of H2B(A117C)$^{Su(C47S)aux}$ (**7**). Calculated *m/z* $[M+H]^+$ 24,225.6 Da, observed 24,230.9 ± 4.0 Da.

MPAA-mediated auxiliary removal from H2B(A117C)$^{Su(C47S)aux}$ (7)

A solution of 600 mM MPAA, 50 mM Na_2HPO_4, pH 7.5 was passed through a 0.45 μm filter, then added directly to the ligation reaction containing the ligation product, **7**, for a final concentration of 150 mM MPAA. The reaction was placed in a container with headspace filled with air equal to 10 times the liquid volume and incubated at 22 °C for 24 h to give H2B(A117C)$^{Su(C47S)}$ (**8**) in 15-30% yield over 2 steps. Final product was visualized by SDS-PAGE and confirmed by LC-ESI-MS analysis employing a gradient of 0-100% D over 15 min. Yield was determined by SDS-PAGE band intensity relative to BSA standards of known concentration. Folding was confirmed by SEC purification of the final product in 50 mM Na_2HPO_4, pH 7.5, at 4 °C, followed by circular dichroism. ESI-MS of H2B(A117C)$^{Su(C47S)}$ (**8**). Calculated *m/z* $[M+H]^+$ 24,149.6 Da, observed 24,153.1 ± 3.2 Da.

SENP1 hydrolysis assay

SEC-purified H2B(A117C)$^{Su(C47S)}$ (**8**) was assayed with the catalytic domain of sentrin-specific protease 1 (SENP1, Boston Biochem). SENP1 (0.05 nmol) was pre-activated in 10 μL buffer containing 50 mM tris, 150 mM NaCl, 12 mM DTT, pH 8 for 20 min at 25 °C. To the reduced SENP1 was then added 10 μL of a

solution containing 0.5 nmol of **8** in 50 mM tris, 150 mM NaCl, 1 mM DTT, pH 7.5. The resulting mixture was incubated for 24 h at 37 °C. The assay was quenched by the addition of 6X Laemmli buffer containing 300 mM DTT and boiled for 5 min, then run on an 18% SDS-PAGE gel at 200 V for 1.5 h and stained with Coomassie.

Supplementary References

(1) Weller, C. E.; Huang, W.; Chatterjee, C. Facile Synthesis of Native and Protease-Resistant Ubiquitylated Peptides. *ChemBioChem* **15**, 1263–1267 (2014).

(2) Zheng, J.-S.; Tang, S.; Qi, Y.-K.; Wang, Z.-P.; Liu, L. Chemical Synthesis of Proteins Using Peptide Hydrazides as Thioester Surrogates. *Nat. Protoc.* **8,** 2483–2495 (2013).

(3) King, D. S.; Fields, C. G.; Fields, G. B. A Cleavage Method Which Minimizes Side Reactions Following Fmoc Solid Phase Peptide Synthesis. *Int. J. Pept. Protein Res.* **36,** 255–266 (1990).

(4) Shah, N. H.; Dann, G. P.; Vila-Perelló, M.; Liu, Z.; Muir, T. W. Ultrafast Protein Splicing Is Common among Cyanobacterial Split Inteins: Implications for Protein Engineering. *J. Am. Chem. Soc.* **134,** 11338–11341 (2012).

(5) Dhall, A.; Wei, S.; Fierz, B.; Woodcock, C. L.; Lee, T.-H.; Chatterjee, C. Sumoylated Human Histone H4 Prevents Chromatin Compaction by Inhibiting Long-Range Internucleosomal Interactions. *J. Biol. Chem.* **289,** 33827–33837 (2014).

(6) Forni, L. G.; Willson, R. L. Thiyl and Phenoxyl Free Radicals and NADH: Direct Observation of One-Electron Oxidation. *Biochem. J.* **240,** 897–903 (1986).

(7) Watanabe, T.; Honda, K. Measurement of the Extinction Coefficient of the Methyl Viologen Cation Radical and the Efficiency of Its Formation by Semiconductor Photocatalysis. *J. Phys. Chem.* **86,** 2617–2619 (1982).

(8) Duling, D. R. Simulation of Multiple Isotropic Spin-Trap EPR Spectra. *J. Magn. Reson. B* **104,** 105–110 (1994).

(9) Guo, Q.; Qian, S. Y.; Mason, R. P. Separation and Identification of DMPO Adducts of Oxygen-Centered Radicals Formed from Organic Hydroperoxides by HPLC-ESR, ESI-MS and MS/MS. *J. Am. Soc. Mass Spectrom.* **14,** 862–871 (2003).

(10) Simon, M. D.; Chu, F.; Racki, L. R.; de la Cruz, C. C.; Burlingame, A. L.; Panning, B.; Narlikar, G. J.; Shokat, K. M. The Site-Specific Installation of Methyl-Lysine Analogs into Recombinant Histones. *Cell* **128,** 1003–1012 (2007).

(11) Chen, J.; Rogers, S. C.; Kavdia, M. Analysis of Kinetics of Dihydroethidium Fluorescence with Superoxide Using Xanthine Oxidase and Hypoxanthine Assay. *Ann. Biomed. Eng.* **41,** 327–337 (2013).

(12) Jezewska, M. M. Xanthine Accumulation during Hypoxanthine Oxidation by Milk Xanthine Oxidase. *Eur. J. Biochem.* **36,** 385–390 (1973).

(13) McGinty, R. K.; Kim, J.; Chatterjee, C.; Roeder, R. G.; Muir, T. W. Chemically Ubiquitylated Histone H2B Stimulates hDot1L-Mediated Intranucleosomal Methylation. *Nature* **453,** 812–816 (2008).
